# Synthesis and Investigations of the Antitumor Effects of First-Row Transition Metal(II) Complexes Supported by Two Fluorinated and Non-Fluorinated β-Diketonates

**DOI:** 10.3390/ijms25042038

**Published:** 2024-02-07

**Authors:** Maura Pellei, Jo’ Del Gobbo, Miriam Caviglia, Valentina Gandin, Cristina Marzano, Deepika V. Karade, Anurag Noonikara Poyil, H. V. Rasika Dias, Carlo Santini

**Affiliations:** 1 School of Science and Technology—Chemistry Division, University of Camerino, Via Madonna delle Carceri (ChIP), Camerino, 62032 Macerata, Italy; jo.delgobbo@unicam.it (J.D.G.); carlo.santini@unicam.it (C.S.); 2 Department of Pharmaceutical and Pharmacological Sciences, University of Padova, Via Marzolo 5, 35131 Padova, Italy; 3 Department of Chemistry and Biochemistry, The University of Texas at Arlington, P.O. Box 19065, Arlington, TX 76019, USA; dxk2232@mavs.uta.edu (D.V.K.); dias@uta.edu (H.V.R.D.)

**Keywords:** first row transition metals, β-diketones, cytotoxicity, spectroscopy, X-ray

## Abstract

The 3*d* transition metal (Mn(II), Fe(II), Co(II), Ni(II), Cu(II) and Zn(II)) complexes, supported by anions of sterically demanding β-diketones, 1,3-dimesitylpropane-1,3-dione (HL^Mes^) and 1,3-bis(3,5-bis(trifluoromethyl)phenyl)-3-hydroxyprop-2-en-1-one (HL^CF3^), were synthesized and evaluated for their antitumor activity. To assess the biological effects of substituents on phenyl moieties, we also synthesized and investigated the analogous metal(II) complexes of the anion of the less bulky 1,3-diphenylpropane-1,3-dione (HL^Ph^) ligand. The compounds [Cu(L^CF3^)_2_], [Cu(L^Mes^)_2_] and ([Zn(L^Mes^)_2_]) were characterized by X-ray crystallography. The [Cu(L^CF3^)_2_] crystallizes with an apical molecule of solvent (THF) and features a rare square pyramidal geometry at the Cu(II) center. The copper(II) and zinc(II) complexes of diketonate ligands, derived from the deprotonated 1,3-dimesitylpropane-1,3-dione (HL^Mes^), adopt a square planar or a tetrahedral geometry at the metal, respectively. We evaluated the antitumor properties of the newly synthesized (Mn(II), Fe(II), Co(II), Ni(II), Cu(II) and Zn(II)) complexes against a series of human tumor cell lines derived from different solid tumors. Except for iron derivatives, cellular studies revealed noteworthy antitumor properties, even towards cancer cells endowed with poor sensitivity to the reference drug cisplatin.

## 1. Introduction

The coordination chemistry of β-diketones continues to attract much interest, due to the ability of related metal complexes to support unique and important catalytic reactions [1,2,3,4,5,6,7]. Even if hindered β-diketones are of high interest for their improved catalytic activity and selectivity [8,9], β-diketones, characterized by the presence of steric bulk, only recently have been made synthetically accessible [10].

The β-diketone scaffold is not very common in nature, though it is the main feature of curcumin and its derivatives [11,12,13] and, in recent years, many metal complexes of curcumin and curcuminoids have been reported and evaluated as anticancer agents [14,15,16,17,18,19,20,21,22,23,24,25,26]. In fact, β-diketones are known to form complexes with almost every metal, and they have been used as supporting ligands for Ti(IV)- [27,28], Ru(II)- [29,30,31,32,33,34,35], Pd(II)- [36] and Pt-based anticancer agents [37,38,39,40,41,42,43]. The β-diketones were used as supporting ligands for heteroleptic Cu(II) complexes, such as Casiopeinas^®^-like compounds (Cas III) [44,45,46,47,48,49], and analogous heteroleptic complexes with β-diketones and *N*,*N*’-chelating aromatic ligands (2,2-bipyridine, 1,10-phenanthroline, and related substituted derivatives) have been studied for their potential antitumor activities [50,51,52,53,54]. The most studied metal-based β-diketone derivatives are the metal-based curcuminoids derivatives, and many of these have been reported and evaluated as anticancer agents [55,56,57,58,59,60,61,62,63]. However, apart from metal–curcumin complexes, very few studies on the anticancer activity of homoleptic first-row transition metal complexes (Mn(II), Fe(II), Co(II), Ni(II), Cu(II) and Zn(II)) with β-diketonate ligands have been reported in the literature to date [51,64,65,66].

As part of our continuous investigation on the chemical and biological properties of metal-containing coordination compounds [67,68,69,70,71,72,73,74,75], we report here a study on the syntheses, characterization and biological evaluation of new homoleptic first-row transition metal(II) complexes containing β-diketone ligands. In particular, we report the synthesis of Mn(II), Fe(II), Co(II), Ni(II), Cu(II) and Zn(II) complexes of the β-diketonate ligands derived from 1,3-bis(3,5-bis(trifluoromethyl)phenyl)-3-hydroxyprop-2-en-1-one (HL^CF3^), 1,3-dimesitylpropane-1,3-dione (HL^Mes^) and 1,3-diphenylpropane-1,3-dione (HL^Ph^), which were selected to systematically vary the electronic properties and solubility of the resulting metal complexes. In addition, fluorine-containing compounds are of relevant interest in modern medicinal chemistry [76,77,78,79,80,81,82], and the substitution of methyl groups by trifluoromethyl groups in a molecule might be expected to induce great changes in molecular and biological properties [83,84,85]. We carried out a screening of the newly synthesized metal(II) compounds against a panel of human cancer cell lines derived from different solid tumors, to investigate the structure–activity relationships.

## 2. Results and Discussion

### 2.1. Synthesis and Characterization

The β-diketones 1,3-bis(3,5-bis(trifluoromethyl)phenyl)propane-1,3-dione (HL^CF3^) [86] and 1,3-dimesityl-propane-1,3-dione (HL^Mes^) [87] were prepared according to procedures set out in previous literature and fully characterized using several methods. HL^Ph^ was obtained from commercial sources and used as received. The sodium salt of the β-diketonate ligand NaL^Mes^ was prepared according to procedures detailed in previous literature [88].

**Scheme 1:**
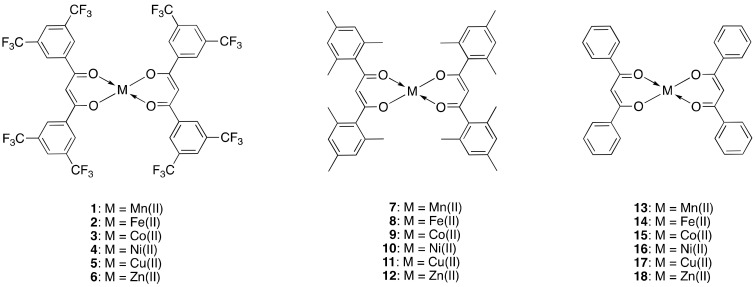
Structure of compounds **1**–**18**.

The complexes [Mn(L^CF3^)_2_(H_2_O)_2_] (**1**), [Fe(L^CF3^)_2_] (**2**), [Co(L^CF3^)_2_(H_2_O)_2_] (**3**) and [Ni(L^CF3^)_2_(H_2_O)_2_] (**4**) (Scheme 1) were synthesized by dissolving the ligand HL^CF3^ in an ethanol solution containing the metal acetate acceptor in a 2:1 stoichiometric ratio. A similar procedure was used for the synthesis in good yield of [Cu(L^CF3^)_2_] (**5**) and [Zn(L^CF3^)_2_] (**6**) by dissolving the corresponding ligand HL^CF3^ in a water/ethanol solution containing the copper(II) acetate monohydrate and the zinc(II) acetate, respectively. The IR spectra were carried out on solid samples of **1**–**6**. They show all the expected absorption bands; in particular, absorptions in the range 2898–3104 cm^−1^ are due to the C-H bonds, medium absorptions at 1564–1629 cm^−1^ are attributable to the asymmetric stretching of the C=O groups and absorptions in the range 1515–1555 cm^−1^ are assigned to the ν(C=C) stretching modes. Bands due to C-F stretching and CF_3_ deformation are at 1169–1173 and 1125–1129 cm^−1^, respectively. The ligand coordination sites involved in bonding with the metal ions were determined by careful comparison of the infrared absorption bands of the complexes with those of the parent ligand HL^CF3^, suggesting the coordination of the ligand as an enolate. The 3226–3374 cm^−1^ regions of the spectra of compounds **1**, **3** and **4** show broad bands, which may be due to lattice and/or coordinated water molecules associated with the complexes. The proton nuclear magnetic resonance (^1^H-NMR) spectrum of [Zn(L^CF3^)_2_] (**6**), recorded in acetone-d_6_ solution at room temperature, show a single set of resonances for the β-diketone moiety, indicating that the protons of the aromatic rings are equivalents, with a shift due to the ligand coordination to the metal center. The ^19^F NMR spectrum of **6** in acetone-d_6_ displayed a singlet at δ -63.35. The electrospray ionization mass spectra (ESI-MS) study was performed by dissolving complexes **1**–**6** in CH_3_CN or CH_3_OH/CH_3_CN and recording the spectrum in positive- and negative-ion mode. The structure of the complexes was confirmed by the presence of peaks attributable to positive fragments of the dissociation of the ligand from the complexes ([Mn(L^CF3^) + CH_3_CN]^+^, [Mn(L^CF3^) + 2CH_3_CN]^+^, [Fe(L^CF3^) + 2CH_3_CN]^+^, [Co(L^CF3^) + 2CH_3_CN]^+^, [Ni(L^CF3^)(H_2_O)_2_ + 2CH_3_CN]^+^, [Cu(L^CF3^) + CH_3_CN]^+^, [Cu(L^CF3^) + 2CH_3_CN]^+^ and [Zn(L^CF3^) + 2H_2_O]^+^) or negative adducts of the complexes such as [Mn(L^CF3^)_2_ + Cl]^−^, [Co(L^CF3^)_2_ + Cl]^−^ and [Ni(L^CF3^)_2_ + Cl]^−^. In addition, in the negative-ion mode spectra, we observed peaks at 495 due to the [L^CF3^]^−^ fragment.

**Figure 1:**
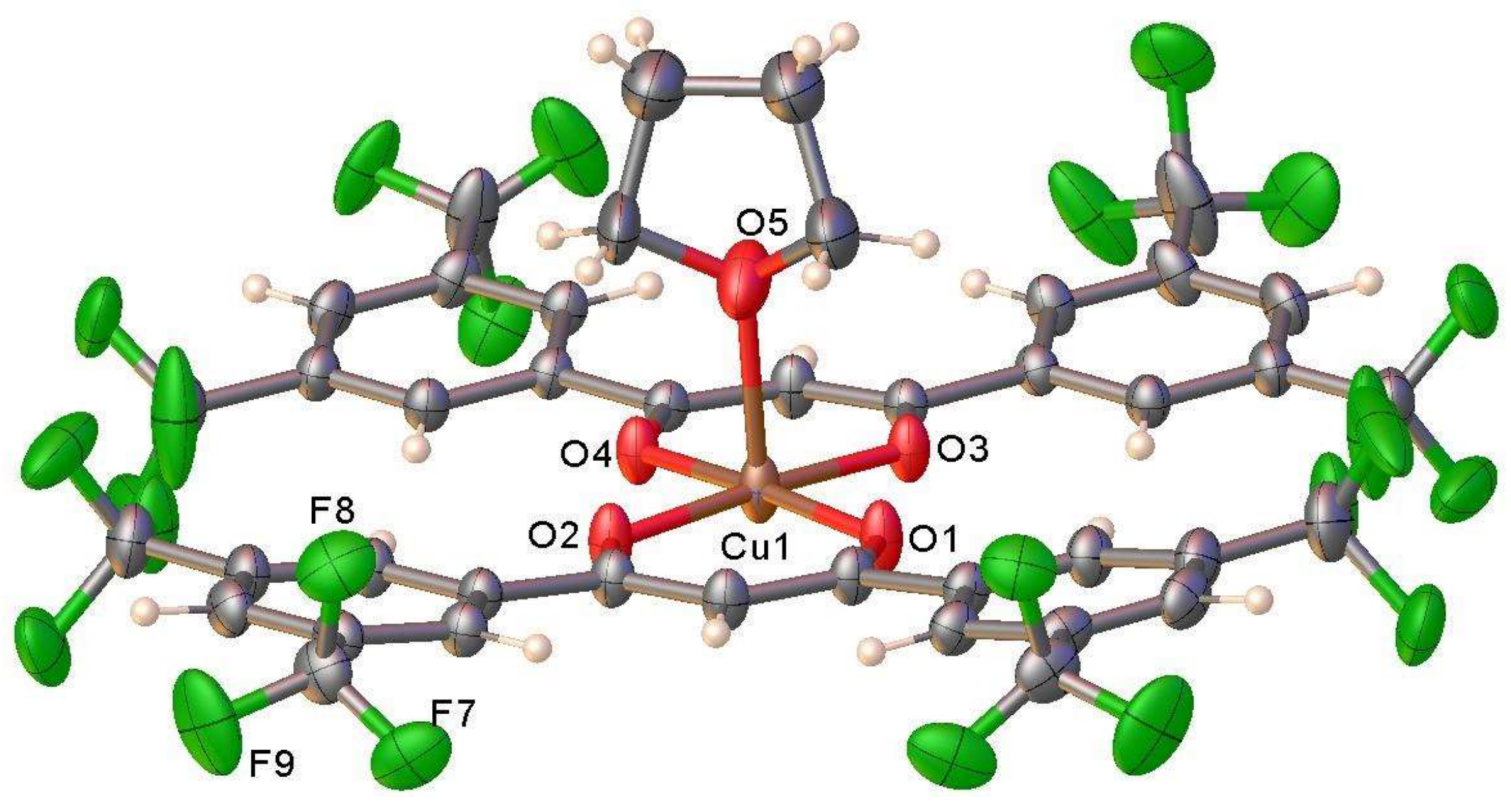
Molecular structure of [Cu(L^CF3^)_2_(THF)].

Compound [Cu(L^CF3^)_2_] (**5**) was characterized by X-ray crystallography. It crystallizes as a THF adduct from acetone/chloroform/tetrahydrofuran mixture. The molecular structure is illustrated in Figure 1 and Figure S3. Detailed bond distances and angles are provided in the Supporting Information (Tables S7–S9). [Cu(L^CF3^)_2_(THF)] is a square pyramidal complex with an apical THF ligand. Such square pyramidal diketonate molecules are very rare in copper(II), and the copper(II) complex [Cu(L^C6F5^)_2_(OH_2_)] derived from the deprotonated 1,3-bis(pentafluorophenyl)propane-1,3-dione (HL^C6F5^) is the only other structurally characterized example in the literature, to our knowledge [89]. The Cu1-O5 distance of 2.232(2) Å is significantly longer than Cu-O distances involving the β-diketone moiety (range from 1.920(1) to 1.924(1) Å). The coordination of the THF molecule does not distort the square planar CuO_4_ base appreciably. The [Cu(L^C6F5^)_2_(OH_2_)] shows similar features, with a significantly longer Cu-O distance to the apical ligand (2.289(3) Å) and a shorter Cu-O distance to the basal diketonates (1.923(2)–1.932(2) Å).

The complexes [Mn(L^Mes^)_2_(H_2_O)_2_] (**7**), [Fe(L^Mes^)_2_] (**8**), [Co(L^Mes^)_2_(H_2_O)_2_] (**9**) and [Ni(L^Mes^)_2_(H_2_O)_2_] (**10**) were synthesized by solubilizing the ligand sodium salt NaL^Mes^ and the corresponding metal acetate in ethanol or methanol solution. The copper(II) complex [Cu(L^Mes^)_2_] (**11**) was synthesized by treating NaL^Mes^ with copper(II) chloride dihydrate in a methanol/ethanol solution. The [Zn(L^Mes^)_2_] (**12**) was synthesized in a water/ethanol solution, using zinc(II) chloride and NaL^Mes^ precursors. The IR spectra were carried out on solid samples of **7**–**12**. Absorptions in the range 2852–3081 cm^−1^ are due to the C-H bonds, medium absorptions in the range 1569–1613 cm^−1^ are attributable to the asymmetric stretching of the C=O groups and absorptions at 1500–1552 cm^−1^ are due to the ν(C=C) stretching modes; all these shifts confirm the coordination of the ligands as enolates. The carbonyl stretching frequencies are comparable with those reported in the literature for analogous Cu(II) complexes supported by bulky β-diketones [90,91]. The 3318–3405 cm^−1^ regions of the spectra of compounds **7**, **9** and **10** show broad bands, which may be due to lattice and/or coordinated water molecules associated with the complexes. The ^1^H-NMR spectrum of [Zn(L^Mes^)_2_] (**12**), recorded in CDCl_3_ solution at room temperature, shows a set of resonances for the β-diketone moiety, with a slight shift due to the coordination to the metal center. The ESI-MS study was performed by dissolving complexes **7**–**12** in CH_3_OH, CH_2_Cl_2_/CH_3_CN or CH_3_OH/CH_3_CN and recording the spectrum in positive- and negative-ion mode. The structure of the complexes was confirmed by the presence of peaks attributable to positive fragments of the adducts of the complexes with H^+^ or Na^+^ ([Mn(L^Mes^)_2_ + H]^+^, [Mn(L^Mes^)_2_ + Na]^+^, [Fe(L^Mes^)_2_ + H]^+^, [Co(L^Mes^)_2_ + H]^+^, [Ni(L^Mes^)_2_ + H]^+^, [Cu(L^Mes^)_2_ + H]^+^, [Cu(L^Mes^)_2_ + Na]^+^, [Zn(L^Mes^)_2_ + H]^+^ and [Zn(L^Mes^)_2_ + Na]^+^), or from the dissociation of the ligand from the complexes ([Mn(L^Mes^)(H_2_O) + H]^+^, [Mn(L^Mes^)(H_2_O) + Na]^+^, [Co(L^Mes^) + 2CH_3_CN]^+^, [Ni(L^Mes^) + CH_3_CN]^+^ and [Ni(L^Mes^) + 2CH_3_CN]^+^). In the negative-ion mode spectra, we observed negative adducts of complexes such as [Co(L^Mes^) + Cl]^−^, [Co(L^Mes^) + 2Cl]^−^, [Co(L^Mes^)_2_ + Cl]^−^ or [Zn(L^Mes^) + 2Cl]^−^, together with peaks at 307 due to the [L^Mes^]^−^ fragment.

**Figure 2:**
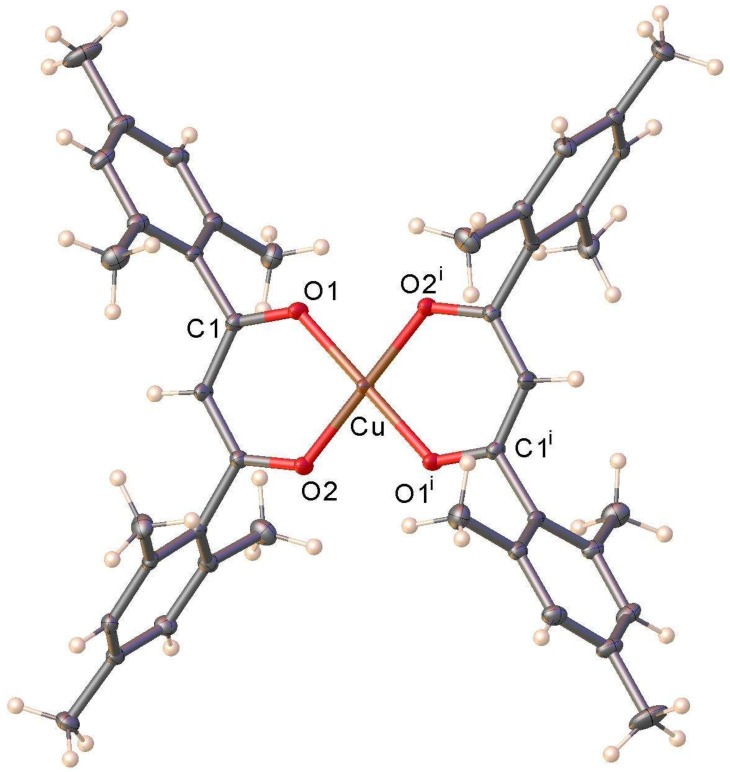
Molecular structure of [Cu(L^Mes^)_2_] (**11**). Selected bond distances (Å) and angles (°): Cu-O1 1.9166(9), Cu-O1^i^ 1.9166(9), Cu-O2 1.9071(9), Cu-O2^i^ 1.9071(9), O1-Cu-O1^i^ 180.0, O2-Cu-O2^i^ 180.0, O1-Cu-O2 93.67(4), O2^i^-Cu-O1^i^ 93.67(4), O2-Cu-O1^i^ 86.33(4).

**Figure 3:**
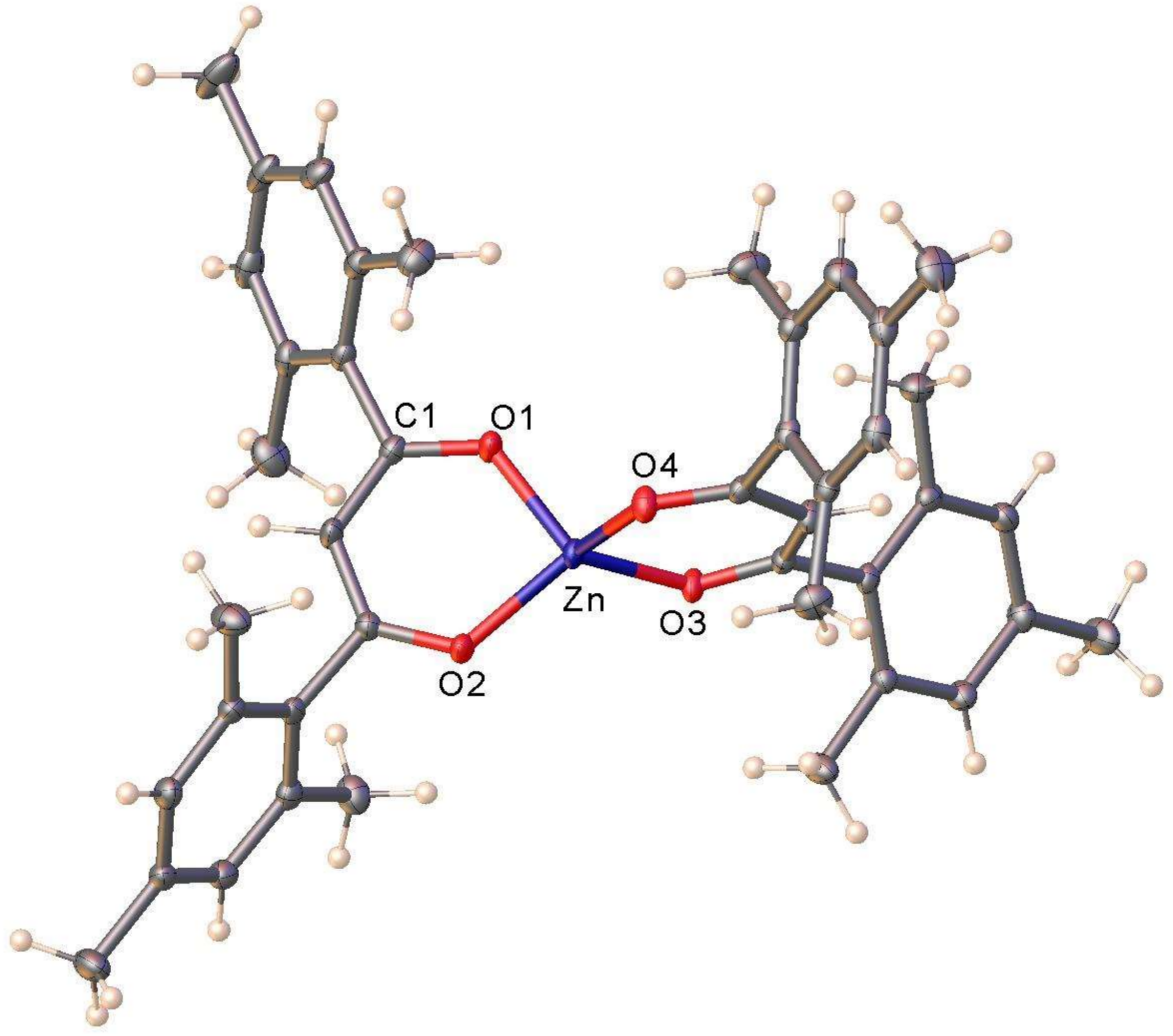
Molecular structure of [Zn(L^Mes^)_2_] (**12**). Selected bond distances (Å) and angles (°): Zn-O1 1.9226(11), Zn-O2 1.9282(11), Zn-O3 1.9384(10), Zn-O4 1.9435(11), O1-Zn-O2 95.78(4), O3-Zn-O4 98.06(4), O1-Zn-O4 111.67(5), O2-Zn-O3 123.15(5), O3-Zn-O1 111.02(5), O2-Zn-O4 117.02(5).

The copper(II) and zinc(II) complexes [Cu(L^Mes^)_2_] (**11**) and [Zn(L^Mes^)_2_] (**12**) have been characterized by X-ray crystallography. Although well authenticated Cu(II) complexes supported by aryl-substituted diketonate complexes are quite common, structural data on the related zinc(II) complexes are very rare [92]. The [Cu(L^Mes^)_2_] is a square planar complex (Figure 2 and Figure S1), while [Zn(L^Mes^)_2_] adopts a distorted tetrahedral geometry at zinc (Figure 3 and Figure S2). Detailed bond distances and angles are provided in the Supporting Information (Tables S1–S6). Interestingly, the Cu-O bond distances of [Cu(L^Mes^)_2_] (average 1.912 Å) are shorter than the Zn-O bond distances of [Zn(L^Mes^)_2_] (average 1.933 Å), despite the less crowded tetrahedral coordination mode of the latter. Structural data on the related [Cu(L^Ph^)_2_] [93] and [Zn(L^Ph^)_2_] [94] are available for a comparison, and display similar basic structures as the [Cu(L^Mes^)_2_] and [Zn(L^Mes^)_2_] analogs with bulker diketonates (i.e., square planar and tetrahedral), respectively. The average Cu-O (1.907 Å) and Zn-O (1.941 Å) distances are also similar. Interestingly, in contrast to [Zn(L^Mes^)_2_], the dimeric version of [Zn(L^Ph^)_2_], resulting from one of the diketonate oxygens bridging the neighboring zinc center, is also known (i.e., [Zn(L^Ph^)_2_]_2_) [94], perhaps as a result of reduced steric demand of the [L^Ph^]^−^ ligand in the latter.

The complexes [Mn(L^Ph^)_2_(H_2_O)_2_] (**13**) [95], [Fe(L^Ph^)_2_] (**14**) [96], [Co(L^Ph^)_2_(H_2_O)_2_] (**15**) [97], [Ni(L^Ph^)_2_(H_2_O)_2_] (**16**) [94], [Cu(L^Ph^)_2_] (**17**) [98,99] and [Zn(L^Ph^)_2_]**^.^**2H_2_O (**18**) [94] (Scheme 1), were synthesized, modifying the procedure reported in the literature by dissolving the ligand HL^Ph^ in an ethanol solution containing the corresponding metal acetate acceptor. The IR spectra were carried out on solid samples of **13**–**18**. They show all the expected absorption bands: absorptions in the range 2970–3061 cm^−1^ due to the C-H bonds, medium absorptions at 1590–1620 cm^−1^ due to the asymmetric stretching of the C=O groups and absorptions in the range 1519–1548 cm^−1^, attributable to the ν(C=C) stretching modes; all these shifts suggest the coordination of the ligands as enolates. The carbonyl stretching frequencies fall in a similar range to compounds **1**–**12**, suggesting little sensitivity to steric or electronic properties. The 3298–3349 cm^−1^ regions of the spectra of compounds **13**, **15**, **16** and **18** show broad bands, which may be due to lattice and/or coordinated water molecules associated with the complexes. The ^1^H-NMR spectrum of [Zn(L^Ph^)_2_]**^.^**2H_2_O (**18**), recorded in CDCl_3_ solution at room temperature, shows a characteristic set of resonances for the β-diketone moiety, with a slight shift due to the coordination to the metal center. The ESI-MS study was performed by dissolving complexes **13**–**18** in CH_3_OH/CH_3_CN, CH_3_CN or CH_3_OH and recording the spectra in positive- and negative-ion mode. The structure of the complexes was confirmed by the presence in the ESI-MS (+) spectra of peaks attributable to positive adducts of the complexes with H^+^ or Na^+^ ([Mn(L^Ph^)_2_ + H]^+^, [Fe(L^Ph^)_2_ + H]^+^ and [Cu(L^Ph^)_2_ + Na]^+^) or to positive fragments of the dissociation of the ligand from the complexes ([Co(L^Ph^) + CH_3_CN]^+^, [Co(L^Ph^) + 2CH_3_CN]^+^, [Ni(L^Ph^) + CH_3_CN]^+^, [Ni(L^Ph^) + 2CH_3_CN]^+^, [Cu(L^Ph^) + H_2_O]^+^, [Zn(L^Ph^)_2_ + H]^+^ and [Zn(L^Ph^)_2_ + Na]^+^). In addition, in the negative-ion mode spectra, we observed peaks at 223 due to the [L^Ph^]^−^ fragment.

### 2.2. Biological Studies

The stability of complexes **1**–**18** in 0.5% dimethyl sulfoxide (DMSO)/physiological solution was evaluated by UV–Vis spectroscopy (Figure S57). Spectra were collected every 24 h in the range of 240–640 nm over 72 h. Exemplificative spectra collected at t = 0 and t = 72 h are reported in the Supplementary Material, showing that all compounds were sufficiently stable under physiological conditions. The newly synthesized metal complexes were evaluated for their cytotoxic activity towards various human cancer cell lines representative of different solid tumors. In particular, the in-house cancer cell panel included examples of human colon (HCT-15), pancreatic (BxPC3), testis (NTERA-2), breast (MCF-7) and small cell lung cancer (SCLC) (U-1285) cancer. The cytotoxicity parameters, expressed in terms of 50% inhibitory concentration (IC_50_) obtained after 72 h of exposure to the colorimetric tetrazolium dye (MTT) assay, are reported in Table 1. Due to its simple structure and well-known pharmacological and toxicological profiles, cisplatin is largely applied as a reference drug for the preliminary in vitro testing of new metallodrug candidates. For comparison reasons, the cytotoxicity of the reference metal-based chemotherapeutic drug cisplatin was assessed under the same experimental conditions. Cytotoxicity data for the corresponding uncoordinated ligands and their salts have been previously reported [88]. The cytotoxicity results showed that, except the iron derivatives **2**, **8** and **14**, all tested complexes demonstrated significant antiproliferative activity towards all cancer cell lines belonging to the in-house cancer cell panel, showing IC_50_ values in the micromolar range. Even if it seems rather difficult to define straightforward structure–activity relationships, as a general consideration, all the metal complexes bearing the 1,3-dimesitylpropane-1,3-dione (HL^Mes^) were on average more effective than the corresponding derivatives, including the 3-bis(3,5-bis(trifluoromethyl)phenyl)-3-hydroxyprop-2-en-1-one (HL^CF3^) and 1,3-diphenylpropane-1,3-dione (HL^Ph^) ligands. In particular, complexes [Mn(L^Mes^)_2_(H_2_O)_2_] (**7**) and [Cu(L^Mes^)_2_] (**11**) were the most effective derivatives of the series, being much more effective than cisplatin towards all tested cancer cell lines. It is noteworthy that, against human colon carcinoma HCT-15 cells, [Mn(L^Mes^)_2_(H_2_O)_2_] (**7**) was up to 15-fold more efficacious than cisplatin in decreasing cell proliferation. Similarly, [Mn(L^CF3^)_2_(H_2_O)_2_] (**1**) proved to be much more effective than the reference metallodrug against colon, testis, pancreatic and breast carcinoma cells whereas compound [Co(L^Mes^)_2_(H_2_O)_2_] (**9**) proved to be much more effective than cisplatin against colon, testis and pancreatic human cancer cells whereas it was slightly less effective on breast carcinoma and SCLC cells.

Cells (3–8 × 10^3^ × well) were treated for 72 h with increasing concentrations (0.5–50 µM range) of tested compounds. Cytotoxicity was assessed by MTT test. The IC_50_ values were calculated by the four-parameter logistic model (*p* < 0.05).

## 3. Materials and Methods

### 3.1. Chemistry

#### 3.1.1. Materials and General Methods

All the reagents were obtained from commercial sources and used as received. Melting point (MP) analysis was performed by an SMP3 Stuart Scientific Instrument (Bibby Sterilin Ltd., London, UK). Elemental analyses (C, H, N, S) (EA) were performed with a Fisons Instruments EA-1108 CHNS-O Elemental Analyzer (Thermo Fisher Scientific Inc., Waltham, MA, USA). Fourier-transform infrared (FT-IR) spectra were recorded from 4000 to 700 cm^−1^ on a PerkinElmer Frontier Instrument (PerkinElmer Inc., Waltham, MA, USA), equipped with an attenuated total reflection (ATR) unit using a universal diamond top-plate as a sample holder. Abbreviations used in the analyses of the FT-IR spectra are as follows: br = broad, m = medium, mbr = medium broad, s = strong, sbr = strong broad, vs = very strong, vsbr = very strong broad, sh = shoulder, w = weak, vw = very weak and wbr = weak broad. Nuclear magnetic resonance (NMR) spectra for the nuclei ^1^H and ^13^C were recorded with a Bruker 500 Ascend Spectrometer (Bruker BioSpin Corporation, Billerica, MA, USA; 500.13 MHz for ^1^H, 125.78 MHz for ^13^C and 470.59 MHz for ^19^F). Tetramethylsilane (SiMe_4_) was used as an external standard for the ^1^H- and ^13^C-NMR spectra. The chemical shifts (δ) are reported in ppm, and the coupling constants (J) are reported in hertz (Hz). Abbreviations used in the analyses of the NMR spectra are as follows: br = broad, d = doublet, m = multiplet, s = singlet, sbr = singlet broad, t = triplet and q = quartet. Electrospray ionization mass spectrometry (ESI-MS) spectra were recorded in positive- (ESI-MS (+)) or negative-ions (ESI-MS (−)) mode on a Waters Micromass ZQ Spectrometer, equipped with a single quadrupole (Waters Corporation, Milford, MA, USA), using a methanol or acetonitrile mobile phase. The compounds were added to reagent grade methanol or acetonitrile to give approximately 0.1 mM solutions. These solutions were injected (1 µL) into the spectrometer fitted with an autosampler. The pump delivered the solutions to the mass spectrometer source at a flow rate of 200 μL/min, and nitrogen was employed both as a drying and nebulizing gas. Capillary voltage was typically 2500 V. The temperature of the source was 100 °C, while the temperature of the desolvation was 400 °C. In the analyses of the ESI-MS spectra, the confirmation of major peaks was supported by the comparison of the observed and predicted isotope distribution patterns, with the latter calculated using the IsoPro 3.1 computer software (T-Tech Inc., Norcross, GA, USA).

The ligand HL^Ph^ was obtained from commercial sources and used as received. The ligands HL^CF3^ [86], HL^Mes^ [87] and the related sodium salt NaL^Mes^ [88] were prepared according to procedures detailed in the literature and were fully characterized. For comparison with the related metal complexes, we reported in the Supplementary Material the spectroscopic characterizations of the sodium salts NaL^CF3^, NaL^Mes^ and NaL^Ph^ (FT-IR (Figures S4, S9 and S13), ^1^H-NMR (Figures S5, S10 and S14), ^13^C{^1^H}-NMR (Figures S6, S11 and S15), ^19^F{^1^H}-NMR (Figure S7) and ESI-MS (+) (Figures S8, S12 and S16) spectra).

#### 3.1.2. Synthesis of [Mn(L^CF3^)_2_(H_2_O)_2_] (**1**)

The ligand HL^CF3^ (1.000 mmol, 0.496 g) was added to a manganese(II) acetate tetrahydrate (0.500 mmol, 0.123 g) solution in ethanol (20 mL), and the reaction was stirred at room temperature for 4 h and refluxed for 3 h. Then, the solution was evaporated at a reduced pressure and the yellow solid product was recovered to give complex **1** with a 76% yield. M.p.: 268 °C. FT-IR (cm^−1^, Figure S17): 3226vbr (O-H); 3100w (C-H); 1622m, 1586m (C=O); 1555m, 1515m (C=C); 1459w, 1446w, 1395m, 1362s, 1277vs, 1217s; 1169s, 1125vs (CF_3_); 1113vs, 959w, 923w, 906s, 894m, 845m, 794m, 730w, 706w. ESI-MS (+) (major positive ions, CH_3_OH/CH_3_CN, Figure S18), *m*/*z* (%): 591 (20) [Mn(L^CF3^) + CH_3_CN]^+^, 632 (100) [Mn(L^CF3^) + 2CH_3_CN]^+^. ESI-MS (−) (major negative ions, CH_3_OH/CH_3_CN), *m/z* (%): 495 (20) [L^CF3^]^−^, 1080 (5) [Mn(L^CF3^)_2_ + Cl]^−^. Elemental analysis calculated for C_38_H_18_F_24_MnO_6_: C 42.20, H 1.68; found: C 42.68, H 1.63.

#### 3.1.3. Synthesis of [Fe(L^CF3^)_2_] (**2**)

Iron(II) acetate (0.500 mmol, 0.087 g) and the ligand HL^CF3^ (1.000 mmol, 0.496 g) were solubilized in ethanol (20 mL). The reaction mixture was stirred at room temperature for 3 h and under reflux for another 3 h. The precipitate formed was filtered and washed in methanol to give the orange complex **2** with a 52% yield. M.p.: 270 °C. FT-IR (cm^−1^, Figure S19): 3096wbr (C-H); 1625m, 1574sh (C=O); 1541s, 1516s (C=C); 1460m, 1446m, 1387m, 1355vs, 1324m, 1276vs, 1216m; 1173s, 1128vs (CF_3_); 1045m, 1003w, 960w, 923s, 907sh, 846m, 793s, 730w, 709m. ESI-MS (+) (major positive ions, CH_3_CN, Figure S20), *m*/*z* (%): 633 (40) [Fe(L^CF3^) + 2CH_3_CN]^+^, 1087 (20) [Fe(L^CF3^)_2_ + H + CH_3_CN]^+^. ESI-MS (−) (major negative ions, CH_3_CN), *m*/*z* (%): 495 (100) [L^CF3^]^−^. Elemental analysis calculated for C_38_H_14_F_24_FeO_4_: C 43.62, H 1.35; found: C 43.17, H 1.40.

#### 3.1.4. Synthesis of [Co(L^CF3^)_2_(H_2_O)_2_] (**3**)

Cobalt acetate tetrahydrate (0.500 mmol, 0.125 g) and the ligand HL^CF3^ (1.000 mmol, 0.496 g) were solubilized in ethanol (30 mL). The reaction was stirred at room temperature for 6 h and refluxed for 3 h. The brick red solution was evaporated under reduced pressure and the reddish solid product formed was dried and recovered to give the orange complex **3** with a 56% yield. M.p.: 266 °C. FT-IR (cm^−1^, Figure S21): 3374wbr (O-H); 3096 (C-H); 1628w, 1569m (C=O); 1538m, 1517s (C=C); 1465m, 1445w, 1409m, 1386m, 1363vs, 1288, 1276vs, 1255s, 1222m, 1202s; 1170s, 1125vs (CF_3_); 1098s, 956m, 923w, 907s, 894w, 847m, 787s, 743w, 710m, 699m. ESI-MS (+) (major positive ions, CH_3_OH/CH_3_CN, Figure S22), *m*/*z* (%): 635 (100) [Co(L^CF3^) + 2CH_3_CN]^+^. ESI-MS (−) (major negative ions, CH_3_OH/CH_3_CN), *m*/*z* (%): 495 (100) [L^CF3^]^−^, 1085 (20) [Co(L^CF3^)_2_ + Cl]^−^. Elemental analysis calculated for C_38_H_18_CoF_24_O_6_: C 43.62, H 1.35; found: C 43.17, H 1.40.

#### 3.1.5. Synthesis of [Ni(L^CF3^)_2_(H_2_O)_2_] (**4**)

Nickel(II) acetate tetrahydrate (0.500 mmol, 0.124 g) and the ligand HL^CF3^ (1.000 mmol, 0.496 g) were solubilized in ethanol (30 mL), forming an opalescent light green solution. The rection was stirred at room temperature for 6 h and then refluxed for 3 h, leading to the formation of a more intense green mixture. The precipitate formed was filtered and dried under reduced pressure to give the green complex **4** with a 61% yield. M.p.: 292 °C. FT-IR (cm^−1^, Figure S23): 3375wbr (O-H); 3096wbr, 3004wbr (C-H); 1628w, 1571m (C=O); 1538m, 1519s (C=C); 1466m, 1445w, 1415m, 1387, 1363vs, 1288, 1276vs, 1256s, 1206s; 1170s, 1125vs (CF_3_); 1098s, 957w, 922vw, 907s, 895w, 847m, 787s, 744w, 731m. ESI-MS (+) (major positive ions, CH_3_OH/CH_3_CN, Figure S24), *m*/*z* (%): 635 (100) [Ni(L^CF3^) + 2CH_3_CN]^+^. ESI-MS (−) (major negative ions, CH_3_OH/CH_3_CN), *m*/*z* (%): 495 (30) [L^CF3^]^−^, 623 (10) [Ni(L^CF3^) + 2Cl]^−^, 1083 (20) [Ni(L^CF3^)_2_ + Cl]^−^. Elemental analysis calculated for C_38_H_18_F_24_NiO_6_: C 42.06, H 1.67; found: C 41.35, H 1.61.

#### 3.1.6. Synthesis of [Cu(L^CF3^)_2_] (**5**)

The ligand HL^CF3^ (1.000 mmol, 0.496 g) and copper(II) acetate monohydrate (0.500 mmol, 0.100 g) were solubilized in a mixture of water (20 mL) and ethanol (20 mL). The solution was left under magnetic stirring at room temperature for 1 h and then refluxed for 3 h. The precipitate was filtered and dried at reduced pressure, to obtain the green complex **5** with a 94% yield. A batch of good quality crystals of [Cu(L^CF3^)_2_(THF)], suitable for X-ray analysis, was obtained by the slow evaporation of an acetone/chloroform/tetrahydrofuran solution of **5**. M.p.: 181 °C. FT-IR (cm^−1^, Figure S25): 3099w (C-H); 1627m, 1564m (C=O); 1539s, 1517s (C=C); 1463m, 1446w, 1395s, 1364s, 1326w, 1279vs, 1187s; 1169s, 1128vs (CF_3_); 962m, 924m, 907s, 846m, 790s, 733w, 713m. ESI-MS (+) (major positive ions, CH_3_OH/CH_3_CN, Figure S26), *m*/*z* (%): 599 (60) [Cu(L^CF3^) + CH_3_CN]^+^, 640 (60) [Cu(L^CF3^) + 2CH_3_CN]^+^. ESI-MS (−) (major negative ions, CH_3_CN), *m*/*z* (%): 495 (100) [L^CF3^]^−^. Elemental analysis calculated for C_38_H_14_CuF_24_O_4_: C 43.30, H 1.34; found: C 44.23, H 1.30.

#### 3.1.7. Synthesis of [Zn(L^CF3^)_2_] (**6**)

Zinc(II) acetate (0.092 g, 0.500 mmol) and HL^CF3^ (1.000 mmol, 0.496 g) were solubilized in ethanol (15 mL). Subsequently, distilled water was added (15 mL), and the mixture was stirred at room temperature overnight. The precipitate was washed with chloroform, filtered and dried under vacuum to give the white complex **6** with a 55% yield. M.p.: 185–188 °C. FT-IR (cm^−1^, Figure S27): 3104w, 2898wbr (C-H); 1629m, 1572m (C=O); 1538m, 1516s (C=C); 1464m, 1444w, 1408m, 1385m, 1361vs, 1291s, 1274vs, 1251s, 1220s, 1198s; 1170s, 1125vs (CF_3_); 954m, 906s, 893m, 847m, 787s, 740w, 730w, 709m. ^1^H-NMR (acetone-d_6_, 293 K, Figure S28): δ 7.45 (s, 2H, COC*H*CO), 8.24 (s, 4H, *p*-C*H*_ar_), 8.76 (s, 8H, *o*-C*H*_ar_). ^1^H-NMR (DMSO-d_6_, 293 K): δ 6.94 (s, 2H, COC*H*CO), 8.20 (s, 4H, *p*-C*H*_ar_), 8.54 (s, 8H, *o*-C*H*_ar_). ^13^C{^1^H}-NMR (acetone-d_6_, 293 K, Figure S29): δ 93.54 (CO*C*HCO); 123.46 (q, ^1^*J*_CF_ = 272 Hz, *C*F_3_); 131.40 (q, ^2^*J*_CF_ = 33 Hz, *C*CF_3_); 124.54, 127.95, 142.21 (*C*H_ar_ and *C*_ar_); 184.11 (*C*O). ^19^F{^1^H}-NMR (acetone-d_6_, 293 K, Figure S30): δ −63.35 (s). ESI-MS (+) (major positive ions, CH_3_CN), *m*/*z* (%): 640 (40) [Zn(L^CF3^) + 2H_2_O]^+^. ESI-MS (−) (major negative ions, CH_3_OH/CH_3_CN), *m*/*z* (%): 495 (100) [L^CF3^]^−^. Elemental analysis (%) calculated for C_38_H_14_F_24_O_4_Zn: C 43.23, H 1.34; found: C 42.43, H 1.34.

#### 3.1.8. Synthesis of [Mn(L^Mes^)_2_(H_2_O)_2_] (**7**)

The ligand NaL^Mes^ (1.000 mmol, 0.330 g) and manganese(II) acetate tetrahydrate (0.500 mmol, 0.123 g) were solubilized in methanol (30 mL), and the reaction was stirred at reflux for 3 h. The brown precipitate was filtered and dried under reduced pressure to obtain complex **7** with a 54% yield. M.p.: 231–234 °C. FT-IR (cm^−1^, Figure S31): 3405br (O-H); 3025vw, 2998vw, 2970vw, 2951w, 2917w, 2858w (C-H); 1611w, 1573sh (C=O); 1552m, 1503s (C=C); 1470sh, 1434s, 1338m, 1371s, 1300m, 1288m, 1165m, 1110w, 1050m, 1029m, 958w, 927w, 850m, 797w, 778m, 725m. ESI-MS (+) (major positive ions, CH_3_OH, Figure S32), *m*/*z* (%): 309 (15) [HL^Mes^ + H]^+^, 331 (25) [HL^Mes^ + Na]^+^, 353 (100) [L^Mes^ + 2Na]^+^, 381 (80) [Mn(L^Mes^)(H_2_O) + H]^+^, 402 (10) [Mn(L^Mes^)(H_2_O) + Na]^+^, 669 (15) [Mn(L^Mes^)_2_ + H]^+^, 692 (20) [Mn(L^Mes^)_2_ + Na]^+^. ESI-MS (−) (major negative ions, CH_3_OH), *m*/*z* (%): 307 (50) [L^Mes^]^−^. Elemental analysis (%) calculated for C_42_H_50_MnO_6_: C 71.47, H 7.14; found: C 71.79, H 7.07.

#### 3.1.9. Synthesis of [Fe(L^Mes^)_2_] (**8**)

To an ethanol solution (20 mL) of iron(II) acetate (0.500 mmol, 0.087 g), the ligand NaL^Mes^ (1.000 mmol, 0.330 g) was added, and the mixture was stirred for 24 h at room temperature. The red precipitate was filtered, dried under reduced pressure and washed in methanol to give the orange complex **8** with an 80% yield. M.p.: 260 °C. FT-IR (cm^−1^, Figure S33): 3028sh, 3008sh, 2951w, 2918w, 2858w (C-H); 1610m, 1574m (C=O); 1529vs, 1507s (C=C); 1472m, 1439m, 1367s, 1341vs, 1306m, 1244w, 1165m, 1109w, 1155m, 1030m, 959w, 926w, 882vw, 848s, 819m, 807m, 780m, 728m. ESI-MS (+) (major positive ions, CH_3_CN, Figure S34), *m*/*z* (%): 670 (80) [Fe(L^Mes^)_2_ + H]^+^, 1001 (30) [Fe(L^Mes^)_3_ + H + Na]^+^. ESI-MS (−) (major negative ions, CH_3_OH), *m*/*z* (%): 307 (50) [L^Mes^]^−^. Elemental analysis calculated for C_42_H_46_FeO_4_: C 75.22, H 6.91; found: C 74.73, H 6.66.

#### 3.1.10. Synthesis of [Co(L^Mes^)_2_(H_2_O)_2_] (**9**)

Cobalt(II) acetate tetrahydrate (0.500 mmol, 0.125 g) and the ligand NaL^Mes^ (1.000 mmol, 0.330 g) were solubilized in ethanol (20 mL) and the reaction was stirred at room temperature for 18 h. The solvent was evaporated at reduced pressure and the solid formed was washed in chloroform and dried under reduced pressure to give the red product **9** with a 51% yield. M.p.: 145 °C. FT-IR (cm^−1^, Figure S35): 3348br (O-H); 3028sh, 3000sh, 2951w, 2918m, 2857w (C-H); 1611m, 1573sh (C=O); 1537s, 1503vs (C=C); 1471s, 1369sbr, 1284m, 1164m, 1110m, 1048m, 1030m, 957w, 928w, 883w, 848s, 782m, 726m. ESI-MS (+) (major positive ions, CH_3_CN/CH_3_OH, Figure S36), *m*/*z* (%): 448 (100) [Co(L^Mes^) + 2CH_3_CN]^+^, 674 (5) [Co(L^Mes^)_2_ + H]^+^. ESI-MS (−) (major negative ions, CH_3_CN/CH_3_OH), *m*/*z* (%): 403 (30) [Co(L^Mes^) + Cl]^−^, 436 (30) [Co(L^Mes^) + 2Cl]^−^, 708 (5) [Co(L^Mes^)_2_ + Cl]^−^. Elemental analysis calculated for C_42_H_50_CoO_6_: C 71.07, H 7.10; found: C 69.92, H 6.94.

#### 3.1.11. Synthesis of [Ni(L^Mes^)_2_(H_2_O)_2_] (**10**)

Nickel(II) acetate tetrahydrate (0.500 mmol, 0.124 g) and the ligand NaL^Mes^ (1.000 mmol, 0.330 g) were solubilized in ethanol (30 mL). The reaction was stirred at room temperature for 6 h and refluxed for 3 h. The precipitate was filtered and dried under reduced pressure to give the green complex **10** with a 51% yield. M.p.: 292 °C. FT-IR (cm^−1^, Figure S37): 3318br (O-H); 3007sh, 2951w, 2917w, 2857w (C-H); 1611m, 1572m (C=O); 1552m, 1500s (C=C); 1473w, 1396vs, 1371s, 1299m, 1285m, 1165m, 1110w, 1049m, 1030m, 957w, 929w, 882w, 848m, 784m, 726m. ESI-MS (+) (major positive ions, CH_3_CN, Figure S38), *m*/*z* (%): 406 (100) [Ni(L^Mes^) + CH_3_CN]^+^, 447 (80) [Ni(L^Mes^) + 2CH_3_CN]^+^, 673 (20) [Ni(L^Mes^)_2_ + H]^+^, 1039 (80) [Ni_2_(L^Mes^)_3_]^+^. Elemental analysis calculated for C_42_H_50_NiO_6_: C 71.10, H 7.10; found: C 69.29, H 7.18.

#### 3.1.12. Synthesis of [Cu(L^Mes^)_2_] (**11**)

The ligand NaL^Mes^ (1.000 mmol, 0.330 g) was solubilized in a mixture of methanol (15 mL) and ethanol (15 mL). Copper(II) chloride dihydrate (0.500 mmol, 0.085 g) was added and the reaction was stirred at room temperature for 24 h. The precipitate was filtered and dried under reduced pressure, giving the grey–green complex **11** with a 68% yield. A batch of good quality crystals of [Cu(L^Mes^)_2_], suitable for X-ray analysis, was obtained by the slow evaporation of an n-hexane solution of **11**. M.p.: 297–301 °C. FT-IR (cm^−1^, Figure S39): 3081w, 2954sh, 2917m, 2852m (C-H); 1613m, 1573w (C=O); 1532s, 1512vs (C=C); 1471s, 1386vs, 1365vs, 1261m, 1165m, 1110mb, 1045m, 1029m, 958m, 931w, 879w, 843s, 808s, 786s, 725s. ESI-MS (+) (major positive ions, CH_3_OH, Figure S40), *m*/*z* (%): 678 (10) [Cu(L^Mes^)_2_ + H]^+^, 700 (100) [Cu(L^Mes^)_2_ + Na]^+^. Elemental analysis (%) calculated for C_42_H_46_CuO_4_: C 74.36, H 6.84; found: C 73.88, H 7.00.

#### 3.1.13. Synthesis of [Zn(L^Mes^)_2_] (**12**)

Zinc(II) chloride (0.500 mmol, 0.068 g) and the ligand NaL^Mes^ (1.00 mmol, 0.330 g) were solubilized in a mixture of distilled water (10 mL) and ethanol (5 mL) and stirred at room temperature for 24 h. The white precipitate was filtered and dried under reduced pressure, obtaining the whitish complex **12** with an 84% yield. A batch of good quality crystals of [Zn(L^Mes^)_2_], suitable for X-ray analysis, was obtained by the slow evaporation of an n-hexane solution of **12**. M.p.: 241–244 °C. FT-IR (cm^−1^, Figure S41): 3019sh, 3003w, 2951w, 2919w, 2859wbr (C-H); 1613s, 1570sh (C=O); 1551vs, 1507vs (C=C); 1472s, 1409vs, 1379s, 1360s, 1302m, 1290m, 1248m, 1166m, 1113w, 1078w, 1050m, 1032m, 957w, 928w, 852s, 817w, 800m, 782m, 726s, 645wbr, 612m. ^1^H-NMR (CDCl_3_, 293 K, Figure S42): δ 2.34 (sbr, 36H, C*H*_3_), 5.71 (s, 2H, COC*H*CO), 6.85 (s, 8H, C*H*_ar_). ^13^C{^1^H}-NMR (CDCl_3_, 293 K, Figure S43): δ 19.51 (*o*-*C*CH_3_); 21.02 (*p*-*C*CH_3_); 105.47 (CO*C*HCO); 128.16, 133.58, 134.52, 138.92 (*C*H_ar_); 191.19 (*C*O). ESI-MS (+) (major positive ions, CH_2_Cl_2_/CH_3_CN) *m*/*z* (%): 309 (10) [HL^Mes^ + H]^+^, 331 (35) [HL^Mes^ + Na]^+^, 679 (40) [Zn(L^Mes^)_2_ + H]^+^, 702 (30) [Zn(L^Mes^)_2_ + Na]^+^. ESI-MS (major negative ions, CH_2_Cl_2_/CH_3_CN) *m*/*z* (%): 170 (30) [ZnCl_3_]^−^, 443 (100) [Zn(L^Mes^) + 2Cl]^−^. Elemental analysis (%) calculated for C_42_H_46_O_4_Zn: C 74.16, H 6.82; found: C 73.42, H 7.03.

#### 3.1.14. Synthesis of [Mn(L^Ph^)_2_(H_2_O)_2_] (**13**)

Manganese(II) acetate tetrahydrate (0.500 mmol, 0.123 g) was added to an ethanol solution (40 mL) of HL^Ph^ (1.000 mmol, 0.224 g). The reaction was stirred at room temperature for 24 h and filtered, and the precipitate was dried under reduced pressure to give the orange complex **13** with a 62% yield. M.p.: 235–238 °C. FT-IR (cm^−1^, Figure S44): 3298br (O-H); 3058w, 3026w (C-H); 1619w, 1593s (C=O); 1545s, 1520vs (C=C); 1478s, 1453s, 1382vsbr, 1301s, 1276s, 1323s, 1184m, 1163m, 1130m, 1071m, 1058m, 1022m, 1000m, 938m, 811w, 787m, 746s, 710s. ESI-MS (+) (major positive ions, CH_3_OH/CH_3_CN, Figure S45), *m*/*z* (%): 225 (5) [HL^Ph^ + H]^+^, 319 (100) [Mn(L^Ph^) + CH_3_CN]^+^, 360 (30) [Mn(L^Ph^) + 2CH_3_CN]^+^, 502 (50) [Mn(L^Ph^)_2_ + H]^+^. ESI-MS (−) (major negative ions, CH_3_OH/CH_3_CN) *m*/*z* (%): 223 (30) [L^Ph^]^−^. Elemental analysis calculated for C_30_H_26_MnO_6_: C 67.04, H 4.88; found: C 67.80, H 4.86.

#### 3.1.15. Synthesis of [Fe(L^Ph^)_2_] (**14**)

Iron(II) acetate (0.500 mmol, 0.087 g) and HL^Ph^ (1.000 mmol, 0.224 g) were solubilized in ethanol (30 mL), and the reaction mixture was stirred at room temperature for 24 h. The precipitate was filtered and washed with methanol to give the brownish-orange complex **14** with a 45% yield. M.p.: 269–273 °C. FT-IR (cm^−1^, Figure S46): 3061wbr, 3029vw, 2970vw (C-H); 1620w, 1590m (C=O); 1519vs (C=C); 1476s, 1452s, 1440m, 1374s, 1355vs, 1311sbr, 1255s, 1180m, 1157w, 1127w, 1067m, 1025m, 1000w, 940m, 841w, 788m, 768msh, 754s, 726s, 712s, 885s, 621s, 549s. ESI-MS (+) (major positive ions, CH_3_OH/CH_3_CN, Figure S47), *m*/*z* (%): 502 (100) [Fe(L^Ph^)_2_ + H]^+^, 783 (20) [Fe_2_(L^Ph^)_3_]^+^. ESI-MS (−) (major negative ions, CH_3_OH/CH_3_CN), *m*/*z* (%): 223 (60) [L^Ph^]^−^. Elemental analysis calculated for C_30_H_22_FeO_4_: C 71.73, H 4.41; found: C 73.43, H 4.49.

#### 3.1.16. Synthesis of [Co(L^Ph^)_2_(H_2_O)_2_] (**15**)

Cobalt(II) acetate tetrahydrate (0.500 mmol, 0.125 g) was added to the ligand HL^Ph^ (1.000 mmol, 0.224 g), solubilized in ethanol (30 mL). The reaction mixture was stirred at room temperature for 16 h and filtered, and the precipitate was dried under reduced pressure. The orange complex **15** was obtained with a 90% yield. M.p.: 221–225 °C. FT-IR (cm^−1^, Figure S48): 3349mbr (O-H); 3060w (C-H); 1620wbr, 1593m, (C=O); 1545s, 1522s (C=C); 1479s, 1454s, 1358sbr, 1303sbr, 1278s, 1233m, 1184m, 1165m, 1153m, 1131m, 1071m, 1061m, 1022m, 1000m, 940m, 926w, 810w, 789m, 745s, 710s. ESI-MS (+) (major positive ions, CH_3_CN, Figure S49), *m*/*z* (%): 323 (55) [Co(L^Ph^) + CH_3_CN]^+^, 364 (100) [Co(L^Ph^) + 2CH_3_CN]^+^. ESI-MS (−) (major negative ions, CH_3_OH/CH_3_CN), *m*/*z* (%): 223 (60) [L^Ph^]^−^. Elemental analysis calculated for C_30_H_26_CoO_6_: C 66.55, H 4.84; found: C 66.75, H 4.75.

#### 3.1.17. Synthesis of [Ni(L^Ph^)_2_(H_2_O)_2_] (**16**)

The ligand HL^Ph^ (1.000 mmol, 0.224 g) was added to a solution of nickel(II) acetate tetrahydrate (0.500 mmol, 0.124 g) in ethanol (30 mL) and the green mixture was stirred at room temperature for 18 h. The precipitate formed was filtered and washed with ethanol to give the light green complex **16** with an 81% yield. M.p.: 298–302 °C. FT-IR (cm^−1^, Figure S50): 3325mbr (O-H); 3060w (C-H); 1620w, 1594s (C=O); 1548s, 1521vs (C=C); 1479s, 1454vs, 1385shvs, 1303sbr, 1279s, 1234s, 1185m, 1163m, 1132m, 1072m, 1063m, 1023m, 1000m, 942m, 926w, 810w, 790m, 744s, 719shs, 711vs. ESI-MS (+) (major positive ions, CH_3_CN, Figure S51), *m*/*z* (%): 322 (50) [Ni(L^Ph^) + CH_3_CN]^+^, 363 (100) [Ni(L^Ph^) + 2CH_3_CN]^+^. ESI-MS (−) (major negative ions, CH_3_OH/CH_3_CN), *m*/*z* (%): 223 (60) [L^Ph^]^−^. Elemental analysis calculated for C_30_H_26_NiO_6_: C 66.58, H 4.84; found: C 65.57, H 4.91.

#### 3.1.18. Synthesis of [Cu(L^Ph^)_2_] (**17**)

Copper(II) acetate monohydrate (0.500 mmol, 0.100 g) was added to an ethanol solution (30 mL) of the ligand HL^Ph^ (1.000 mmol, 0.224 g). The reaction mixture was stirred at room temperature for 16 h and, after filtration, the precipitate was dried under reduced pressure to give the dark green complex **17** with a 95% yield. M.p.: 318–322 °C. FT-IR (cm^−1^, Figure S52): 3063m, 3026m (C-H); 1619w, 1592s (C=O); 1538vs, 1523vs (C=C); 1483s, 1473s, 1454vs, 1395vs, 1313vs, 1320s, 1182s, 1166s, 1149s, 1128s, 1094s, 1066s, 1022s, 997s, 972s, 944s, 933s, 841m, 809m, 790s, 740vs, 706vs. ESI-MS (+) (major positive ions, CH_3_OH, Figure S53), *m*/*z* (%): 225 (20) [HL^Ph^ + H]^+^, 304 (100) [Cu(L^Ph^) + H_2_O]^+^, 532 (40) [Cu(L^Ph^)_2_ + Na]^+^, 567 (40) [Cu(L^Ph^)_2_ + 2H_2_O + Na]^+^. ESI-MS (−) (major negative ions, CH_3_OH), *m*/*z* (%): 223 (30) [L^Ph^]^−^, 255 (30) [L^Ph^ + CH_3_OH]^−^. Elemental analysis calculated for C_30_H_22_CuO_4_: C 70.65, H 4.35; found: C 70.88, H 4.28.

#### 3.1.19. Synthesis of [Zn(L^Ph^)_2_]^.^2H_2_O (**18**)

To the ligand HL^Ph^ (1.000 mmol, 0.224 g) solubilized in ethanol (50 mL), zinc(II) acetate (0.092 g, 0.500 mmol) was added, and the reaction was stirred at room temperature for 24 h. The mixture was filtered, and the precipitate was dried at reduced pressure. The light green complex **18** was obtained with an 88% yield. M.p.: 221–225 °C. FT-IR (cm^−1^, Figure S54): 3307br (O-H); 3059w, 3026w (C-H); 1620w, 1594s (C=O); 1547s, 1521vs (C=C); 1479s, 1454s, 1386vs, 1302s, 1276s, 1233sm, 1184m, 1164m, 1131m, 1071m, 1060m, 1022m, 1000m, 939m, 926m, 811w, 788m, 746s, 711s. ^1^H-NMR (CDCl_3_, 293 K, Figure S55): δ 6.89 (s, 1H, COC*H*CO); 7.48–8.05 (m, 20H, C*H*_ar_). ^13^C{^1^H}-NMR (CDCl_3_, 293 K, Figure S56): δ 95.00 (CO*C*HCO); 127.65, 128.39, 131.66, 139.70 (*C*H_ar_ and *C*_ar_); 188.46 (*C*O). ESI-MS (+) (major positive ions, EtOH), *m*/*z* (%): 247 (30) [HL^Ph^ + Na]^+^, 511 (70) [Zn(L^Ph^)_2_ + H]^+^, 533 (20) [Zn(L^Ph^)_2_ + Na]^+^. ESI-MS (−) (major negative ions, EtOH), *m*/*z* (%): 223 (60) [L^Ph^]^−^, 359 (40) [Zn(L^Ph^) + 2Cl]^−^, 545 (10) [Zn(L^Ph^)_2_ + Cl]^−^. Elemental analysis calculated for C_30_H_26_O_6_Zn: C 65.76, H 4.78; found: C 65.94, H 4.71.

### 3.2. X-ray Data Collection and Structure Determination

A suitable crystal covered with a layer of hydrocarbon/Paratone-N oil was selected and mounted on a Cryo-loop, then immediately placed in the low temperature nitrogen stream. The X-ray intensity data of [Cu(L^Mes^)_2_] (**11**) and [Zn(L^Mes^)_2_] (**12**) were measured at 100 K while the data of [Cu(L^CF3^)_2_(THF)] were measured at 200 K (due to crystal cracking issues at 100 K) on a Bruker SMART APEX II CCD area detector system, equipped with an Oxford Cryosystems 700 series cooler, a graphite monochromator, and a Mo Kα fine-focus sealed tube (λ = 0.71073 Å). Intensity data were processed using the Bruker APEX3, Version 2016.5-0 program suite. Absorption corrections were applied using SADABS 2016/2 [100]. Initial atomic positions were located by SHELXT Version 2018/2 [101], and the structures of the compounds were refined by the least-squares method using SHELXL Version 2019/3 [102] within Olex2-1.5 GUI [103]. All the non-hydrogen atoms were refined anisotropically. Hydrogen atoms were included at calculated positions and refined using appropriate riding models. The copper center of [Cu(L^Mes^)_2_] molecule sits on a center of inversion. [Cu(L^CF3^)_2_**(**THF)] crystallizes in the P-1 space group, with two additional molecules of THF in the asymmetric unit. All three THF molecules and fluorine atoms of six CF_3_ groups show positional disorder, which was resolved satisfactorily. The X-ray structural figures were generated using Olex2. CCDC 2279303–2279305 files contain the supplementary crystallographic data. These data files have been deposited at Cambridge Crystallographic Data Centre (CCDC), 12 Union Road, Cambridge, CB2 1EZ, UK). Additional details are provided in the Supporting Information section.

### 3.3. Biology

Metal complexes were dissolved in DMSO just before the experiment, and a calculated volume of the drug solution was added to the cell growth medium to a final solvent concentration of 0.5%, which had no detectable effects on cell viability. Cisplatin was dissolved in 0.9% sodium chloride solution. MTT (3-(4,5-dimethylthiazol-2-yl)-2,5-diphenyltetrazolium bromide) and cisplatin were obtained from Sigma Chemical Co, St. Louis, MO, USA.

### 3.4. Cell Cultures

Human SCLC (U1285), testicular (NTERA-2), colon (HCT-15), breast (MCF-7) and pancreatic (BxPC3) carcinoma cell lines were obtained from American Type Culture Collection (ATCC, Rockville, MD, USA). Cell lines were maintained in the logarithmic phase at 37 °C under a 5% carbon dioxide atmosphere using the Roswell Park Memorial Institute (RPMI)-1640 or Dulbecco’s Modified Eagle Medium (DMEM) medium (EuroClone, Milan, Italy) containing 10% fetal calf serum (EuroClone, Milan, Italy), antibiotics (50 units per mL penicillin and 50 μg mL^−1^ streptomycin) and 2 mM L-glutamine.

### 3.5. MTT Assay

The growth inhibitory effect toward tumor cells was evaluated by means of the MTT assay, as previously described [104]. Briefly, 3–8 × 10^3^ cells/well, dependent upon the growth characteristics of the cell line, were seeded in 96-well microplates in a growth medium (100 μL). After 24 h, the medium was removed and replaced with a fresh one containing the compound to be studied at the appropriate concentration (0.5–50 μM concentration range). Triplicate cultures were established for each treatment. IC_50_ values, the drug concentrations that reduce the mean absorbance at 570 nm to 50% of those in the untreated control wells, were calculated using the four-parameter logistic (4-PL) model. The evaluation was based on means from at least three independent experiments.

## 4. Conclusions

In this study, the β-diketone ligands 1,3-dimesitylpropane-1,3-dione (HL^Mes^), 1,3-bis(3,5-bis(trifluoromethyl)phenyl)-3-hydroxyprop-2-en-1-one (HL^CF3^) and 1,3-diphenylpropane-1,3-dione (HL^Ph^) were used to synthesize novel homoleptic complexes with 3*d* transition metals (Mn(II), Fe(II), Co(II), Ni(II), Cu(II) and Zn(II)). The compounds were fully characterized both in the solid state and in solution. The compound [Cu(L^CF3^)_2_] crystallizes as a square pyramidal complex [Cu(L^CF3^)_2_(THF)] with an apical THF ligand. The non-fluorinated complex [Cu(L^Mes^)_2_] features a square planar geometry at copper, while [Zn(L^Mes^)_2_] adopts a distorted tetrahedral geometry at zinc.

In general, apart from Fe(II) complexes, the newly synthetized metal(II) complexes showed a marked cytotoxic activity, even against human colon cancer cells with poor sensitivity to cisplatin. The preliminary screening allowed the identification of the complexes [Mn(L^Mes^)_2_(H_2_O)_2_] (**7**) and [Cu(L^Mes^)_2_] (**11**) as the most effective derivatives of the series, being much more effective than cisplatin towards all tested cancer cell lines. In particular, against HCT-15 cancer cells, [Mn(L^Mes^)_2_(H_2_O)_2_] (**7**) was up to 15-fold more efficacious than cisplatin in decreasing cell proliferation, thus providing a foundation for also developing β-diketonate Mn(II) complexes, other than Cu(II) ones, as anticancer agents.

## Figures and Tables

**Table 1:** Cytotoxicity profiles.

	IC_50_ (µM) ± S.D.				
	HCT-15	U-1285	NTERA-2	BxPC-3	MCF-7
[Mn(L^CF3^)_2_(H_2_O)_2_] (**1**)	2.9 ± 0.4	17.2 ± 3.0	2.8 ± 1.2	3.7 ± 0.7	2.7 ± 0.6
[Fe(L^CF3^)_2_] (**2**)	>50	>50	>50	>50	>50
[Co(L^CF3^)_2_(H_2_O)_2_] (**3**)	26.4 ± 3.8	39.5 ± 4.6	18.5 ± 3.8	10.5 ± 2.3	14.2 ± 4.2
[Ni(L^CF3^)_2_(H_2_O)_2_] (**4**)	26.1 ± 5.2	37.2 ± 3.3	16.4 ± 3.2	19.5 ± 3.0	21.3 ± 3.2
[Cu(L^CF3^)_2_] (**5**)	15.8 ± 3.2	12.1 ± 2.3	12.2 ± 1.5	15.5 ± 2.4	25.3 ± 4.1
[Zn(L^CF3^)_2_] (**6**)	11.2 ± 3.2	16.5 ± 2.7	10.0 ± 1.2	11.2 ± 0.9	8.2 ± 2.5
[Mn(L^Mes^)_2_(H_2_O)_2_] (**7**)	1.2 ± 0.7	5.3 ± 1.2	1.3 ± 0.6	2.9 ± 0.6	2.3 ± 0.4
[Fe(L^Mes^)_2_] (**8**)	>50	>50	>50	>50	>50
[Co(L^Mes^)_2_(H_2_O)_2_] (**9**)	6.8 ± 2.1	9.2 ± 2.2	3.1 ± 0.7	8.4 ± 2.5	11.8 ± 2.6
[Ni(L^Mes^)_2_(H_2_O)_2_] (**10**)	31.7 ± 4.3	33.3 ± 6.4	15.5 ± 3.7	11.2 ± 1.1	23.2 ± 2.8
[Cu(L^Mes^)_2_] (**11**)	2.5 ± 1.0	4.1 ± 1.2	3.0 ± 0.5	1.2 ± 0.4	3.2 ± 0.6
[Zn(L^Mes^)_2_] (**12**)	8.9 ± 2.1	16.1 ± 3.5	2.5 ± 0.5	2.0 ± 0.6	3.8 ± 1.0
[Mn(L^Ph^)_2_(H_2_O)_2_] (**13**)	13.3 ± 2.0	8.7 ± 1.6	4.8 ± 1.4	6.0 ± 1.3	5.6 ± 1.2
[Fe(L^Ph^)_2_] (**14**)	>50	>50	41.3 ± 6.5	>50	49.9 ± 5.2
[Co(L^Ph^)_2_(H_2_O)_2_] (**15**)	20.4 ± 6.1	44.8 ± 5.2	25.5 ± 3.9	10.8 ± 3.1	16.5 ± 3.3
[Ni(L^Ph^)_2_(H_2_O)_2_] (**16**)	18.7 ± 3.9	15.4 ± 2.1	6.9 ± 1.3	12.4 ± 3.2	7.2 ± 2.7
[Cu(L^Ph^)_2_] (**17**)	4.9 ± 1.0	9.6 ± 1.3	4.8 ± 0.9	6.7 ± 0.6	5.4 ± 0.5
[Zn(L^Ph^)_2_]^.^2H_2_O (**18**)	7.2 ± 2.1	10.6 ± 1.8	11.2 ± 2.2	10.9 ± 2.2	13.2 ± 2.5
Cisplatin	18.5 ± 2.2	8.3 ± 1.4	14.6 ± 3.0	11.9 ± 1.3	11.0 ± 0.8

## Data Availability

CCDC 2279303-2279305 contain the supplementary crystallographic data for this paper. These data have been deposited at The Cambridge Crystallographic Data Centre, 12 Union Road, Cambridge CB2 1EZ, UK; Fax: +44-1223-336033.

## Supplementary Materials

The following supporting information can be downloaded at: https://www.mdpi.com/article/10.3390/ijms25042038/s1.

## References

[1] Crossman A.S., Marshak M.P., Constable E.C., Parkin G., Que L. (2021). 1.11—β-Diketones: Coordination and Application. Comprehensive Coordination Chemistry III.

[2] Stalpaert M., De Vos D. (2018). Stabilizing Effect of Bulky β-Diketones on Homogeneous Mo Catalysts for Deoxydehydration. ACS Sustain. Chem. Eng..

[3] Lo J.C., Gui J., Yabe Y., Pan C.M., Baran P.S. (2014). Functionalized olefin cross-coupling to construct carbon-carbon bonds. Nature.

[4] Shafir A., Buchwald S.L. (2006). Highly selective room-temperature copper-catalyzed C-N coupling reactions. J. Am. Chem. Soc..

[5] Baik T.G., Luis A.L., Wang L.C., Krische M.J. (2001). Diastereoselective cobalt-catalyzed Aldol and Michael cycloreductions. J. Am. Chem. Soc..

[6] Kang S.K., Lee S.H., Lee D. (2000). Copper-catalyzed N-arylation of amines with hypervalent iodonium salts. Synlett.

[7] Isayama S., Mukaiyama T. (1989). Hydration of Olefins with Molecular Oxygen and Triethylsilane Catalyzed by Bis(trifluoroacetylacetonato)cobalt(II). Chem. Lett..

[8] Krajewski S.M., Crossman A.S., Akturk E.S., Suhrbier T., Scappaticci S.J., Staab M.W., Marshak M.P. (2019). Sterically encumbered beta-diketonates and base metal catalysis. Dalton Trans..

[9] Akturk E.S., Scappaticci S.J., Seals R.N., Marshak M.P. (2017). Bulky beta-Diketones Enabling New Lewis Acidic Ligand Platforms. Inorg. Chem..

[10] Crossman A.S., Larson A.T., Shi J.X., Krajewski S.M., Akturk E.S., Marshak M.P. (2019). Synthesis of Sterically Hindered beta-Diketones via Condensation of Acid Chlorides with Enolates. J. Org. Chem..

[11] Chen C.Y., Lien J.C., Chen C.Y., Hung C.C., Lin H.C. (2021). Design, synthesis and evaluation of novel derivatives of curcuminoids with cytotoxicity. Int. J. Mol. Sci..

[12] Goel A., Kunnumakkara A.B., Aggarwal B.B. (2008). Curcumin as Curecumin: From kitchen to clinic. Biochem. Pharmacol..

[13] Krishnankutty K., Venugopalan P. (1998). Metal chelates of curcuminoids. Synth. React. Inorg. Met. Org. Chem..

[14] Meza-Morales W., Alejo-Osorio Y., Alvarez-Ricardo Y., Obregon-Mendoza M.A., Machado-Rodriguez J.C., Arenaza-Corona A., Toscano R.A., Ramirez-Apan M.T., Enriquez R.G. (2023). Homoleptic Complexes of Heterocyclic Curcuminoids with Mg(II) and Cu(II): First Conformationally Heteroleptic Case, Crystal Structures, and Biological Properties. Molecules.

[15] Prasad S., DuBourdieu D., Srivastava A., Kumar P., Lall R. (2021). Metal-Curcumin Complexes in Therapeutics: An Approach to Enhance Pharmacological Effects of Curcumin. Int. J. Mol. Sci..

[16] Meza-Morales W., Estevez-Carmona M.M., Alvarez-Ricardo Y., Obregon-Mendoza M.A., Cassani J., Ramirez-Apan M.T., Escobedo-Martinez C., Soriano-Garcia M., Reynolds W.F., Enriquez R.G. (2019). Full Structural Characterization of Homoleptic Complexes of Diacetylcurcumin with Mg, Zn, Cu, and Mn: Cisplatin-level Cytotoxicity in Vitro with Minimal Acute Toxicity in Vivo. Molecules.

[17] Meza-Morales W., Machado-Rodriguez J.C., Alvarez-Ricardo Y., Obregon-Mendoza M.A., Nieto-Camacho A., Toscano R.A., Soriano-Garcia M., Cassani J., Enriquez R.G. (2019). A New Family of Homoleptic Copper Complexes of Curcuminoids: Synthesis, Characterization and Biological Properties. Molecules.

[18] Zhang W., Chen C.M., Shi H.F., Yang M.Y., Liu Y., Ji P., Chen H.J., Tan R.X., Li E.G. (2016). Curcumin is a biologically active copper chelator with antitumor activity. Phytomedicine.

[19] Mendiguchia B.S., Aiello I., Crispini A. (2015). Zn(ii) and Cu(ii) complexes containing bioactive O,O-chelated ligands: Homoleptic and heteroleptic metal-based biomolecules. Dalton Trans..

[20] Wanninger S., Lorenz V., Subhan A., Edelmann F.T. (2015). Metal complexes of curcumin—Synthetic strategies, structures and medicinal applications. Chem. Soc. Rev..

[21] Leung M.H.M., Harada T., Kee T.W. (2013). Delivery of curcumin and medicinal effects of the copper(II)-curcumin complexes. Curr. Pharm. Des..

[22] Zhou S., Xue X., Jiang B., Tian Y. (2012). Metal complexes of a novel bis-β-diketone-type ligand and its copper(II) complexes of two-photon biological imaging. Sci. China Chem..

[23] Aliaga-Alcalde N., Marques-Gallego P., Kraaijkamp M., Herranz-Lancho C., den Dulk H., Gorner H., Roubeau O., Teat S.J., Weyhermuller T., Reedijk J. (2010). Copper Curcuminoids Containing Anthracene Groups: Fluorescent Molecules with Cytotoxic Activity. Inorg. Chem..

[24] Barik A., Mishra B., Kunwar A., Kadam R.M., Shen L., Dutta S., Padhye S., Satpati A.K., Zhang H.Y., Priyadarsini K.I. (2007). Comparative study of copper(II)-curcumin complexes as superoxide dismutase mimics and free radical scavengers. Eur. J. Med. Chem..

[25] Thompson K.H., Bohmerle K., Polishchuk E., Martins C., Toleikis P., Tse J., Yuen V., McNeill J.H., Orvig C. (2004). Complementary inhibition of synoviocyte, smooth muscle cell or mouse lymphoma cell proliferation by a vanadyl curcumin complex compared to curcumin alone. J. Inorg. Biochem..

[26] Krishnankutty K., John V.D. (2003). Synthesis, characterization, and antitumour studies of metal chelates of some synthetic curcuminoids. Synt. React. Inorg. Met. Org. Chem..

[27] Schilling T., Keppler K.B., Heim M.E., Niebch G., Dietzfelbinger H., Rastetter J., Hanauske A.R. (1995). Clinical phase I and pharmacokinetic trial of the new titanium complex budotitane. Investig. New Drugs.

[28] Bischoff H., Berger M.R., Keppler B.K., Schmähl D. (1987). Efficacy of β-diketonato complexes of titanium, zirconium, and hafnium against chemically induced autochthonous colonic tumors in rats. J. Cancer Res. Clin. Oncol..

[29] Kljun J., Turel I. (2017). β-Diketones as Scaffolds for Anticancer Drug Design—From Organic Building Blocks to Natural Products and Metallodrug Components. Eur. J. Inorg. Chem..

[30] Seršen S., Kljun J., Kryeziu K., Panchuk R., Alte B., Körner W., Heffeter P., Berger W., Turel I. (2015). Structure-related mode-of-action differences of anticancer organoruthenium complexes with β-diketonates. J. Med. Chem..

[31] Aliende C., Pérez-Manrique M., Jalón F.A., Manzano B.R., Rodríguez A.M., Cuevas J.V., Espino G., Martínez M.Á., Massaguer A., González-Bártulos M. (2012). Preparation of new half sandwich ruthenium arene complexes with aminophosphines as potential chemotherapeutics. J. Inorg. Biochem..

[32] Vock C.A., Renfrew A.K., Scopelliti R., Juillerat-Jeanneret L., Dyson P.J. (2008). Influence of the diketonato ligand on the cytotoxicities of [Ru(η6-p-cymene)-(R2acac)(PTA)]+ complexes (PTA = 1,3,5-triaza-7-phosphaadamantane). Eur. J. Inorg. Chem..

[33] Melchart M., Habtemariam A., Parsons S., Sadler P.J. (2007). Chlorido-, aqua-, 9-ethylguanine- and 9-ethyladenine-adducts of cytotoxic ruthenium arene complexes containing O,O-chelating ligands. J. Inorg. Biochem..

[34] Fernández R., Melchart M., Habtemariam A., Parsons S., Sadler P.J. (2004). Use of chelating ligands to tune the reactive site of half-sandwich ruthenium(II)-arene anticancer complexes. Chem. Eur. J..

[35] Wu A., Kennedy D.C., Patrick B.O., James B.R. (2003). Ruthenium(II) acetylacetonato-sulfoxide complexes. Inorg. Chem. Commun..

[36] Do Couto Almeida J., Marzano I.M., De Paula F.C.S., Pivatto M., Lopes N.P., De Souza P.C., Pavan F.R., Formiga A.L.B., Pereira-Maia E.C., Guerra W. (2014). Complexes of platinum and palladium with β-diketones and DMSO: Synthesis, characterization, molecular modeling, and biological studies. J. Mol. Struct..

[37] Wilson J.J., Lippard S.J. (2012). In vitro anticancer activity of cis-diammineplatinum(II) complexes with β-diketonate leaving group ligands. J. Med. Chem..

[38] Muscella A., Calabriso N., Vetrugno C., Fanizzi F.P., De Pascali S.A., Marsigliante S. (2011). The signalling axis mediating neuronal apoptosis in response to [Pt(O,O′-acac)(γ-acac)(DMS)]. Biochem. Pharmacol..

[39] Muscella A., Calabriso N., Vetrugno C., Fanizzi F.P., De Pascali S.A., Storelli C., Marsigliante S. (2011). The platinum (II) complex [Pt(O,O’-acac)(γ-acac)(DMS)] alters the intracellular calcium homeostasis in MCF-7 breast cancer cells. Biochem. Pharmacol..

[40] Muscella A., Calabriso N., Vetrugno C., Urso L., Fanizzi F.P., De Pascali S.A., Marsigliante S. (2010). Sublethal concentrations of the platinum(II) complex [Pt(O,O′-acac) (γ-acac)(DMS)] alter the motility and induce anoikis in MCF-7 cells. Br. J. Pharmacol..

[41] Muscella A., Calabriso N., Fanizzi F.P., De Pascali S.A., Urso L., Ciccarese A., Migoni D., Marsigliante S. (2008). [Pt(O,O′-acac)(γ-acac)(DMS)], a new Pt compound exerting fast cytotoxicity in MCF-7 breast cancer cells via the mitochondrial apoptotic pathway. Br. J. Pharmacol..

[42] Schwartz P., Meischen S.J., Gale G.R., Atkins L.M., Smith A.B., Walker E.M. (1977). Preparation and antitumor evaluation of water-soluble derivatives of dichloro(1,2-diaminocyclohexane)platinum(II). Cancer Treat. Rep..

[43] Cleare M.J., Hoeschele J.D. (1973). Anti-tumour platinum compounds. Relationship between structure and activity. Platin. Met. Rev..

[44] Figueroa-DePaz Y., Resendiz-Acevedo K., Dávila-Manzanilla S.G., García-Ramos J.C., Ortiz-Frade L., Serment-Guerrero J., Ruiz-Azuara L. (2022). DNA, a target of mixed chelate copper(II) compounds (Casiopeinas®) studied by electrophoresis, UV–vis and circular dichroism techniques. J. Inorg. Biochem..

[45] Serment-Guerrero J., Bravo-Gomez M.E., Lara-Rivera E., Ruiz-Azuara L. (2017). Genotoxic assessment of the copper chelated compounds Casiopeinas: Clues about their mechanisms of action. J. Inorg. Biochem..

[46] Correia I., Borovic S., Cavaco I., Matos C.P., Roy S., Santos H.M., Fernandes L., Capelo J.L., Ruiz-Azuara L., Pessoa J.C. (2017). Evaluation of the binding of four anti-tumor Casiopeínas® to human serum albumin. J. Inorg. Biochem..

[47] Bravo-Gómez M.E., de la Paz A.L.H., Gracia-Mora I. (2013). Antineoplastic evaluation of two mixed chelate copper complexes (casiopeínas®) in HCT-15 xenograft model. J. Mex. Chem. Soc..

[48] García-Ramos J.C., Tovar-Tovar A., Hernández-Lima J., Cortés-Guzmán F., Moreno-Esparza R., Ruiz-Azuara L. (2011). A new kind of intermolecular stacking interaction between copper (II) mixed chelate complex (Casiopeína III-ia) and adenine. Polyhedron.

[49] Ruiz-Azuara L., Bravo-Gomez M.E. (2010). Copper Compounds in Cancer Chemotherapy. Curr. Med. Chem..

[50] Aguilar-Jiménez Z., Espinoza-Guillén A., Resendiz-Acevedo K., Fuentes-Noriega I., Mejía C., Ruiz-Azuara L. (2023). The Importance of Being Casiopeina as Polypharmacologycal Profile (Mixed Chelate&ndash; Copper (II) Complexes and Their In Vitro and In Vivo Activities). Inorganics.

[51] Paixao D.A., de Oliveira B.C.A., Almeida J.D., Sousa L.M., Lopes C.D., Carneiro Z.A., Tezuka D.Y., Clavijo J.C.T., Ellena J., Polloni L. (2020). Crystal structure, anti-Trypanosoma cruzi and cytotoxic activities of Cu(II) complexes bearing beta-diketone and alpha-diimine ligands. Inorg. Chim. Acta.

[52] Polloni L., Silva A.C.D., Teixeira S.C., Azevedo F.V.D., Zoia M.A.P., da Silva M.S., Lima P., Correia L.I.V., Almeida J.D., da Silva C.V. (2019). Action of copper(II) complex with beta-diketone and 1,10-phenanthroline (CBP-01) on sarcoma cells and biological effects under cell. Biomed. Pharmacother..

[53] Malekshah R.E., Salehi M., Kubicki M., Khaleghian A. (2019). Biological studies and computational modeling of two new copper complexes derived from beta-diketones and their nano-complexes. J. Coord. Chem..

[54] Malekshah R.E., Salehi M., Kubicki M., Khaleghian A. (2018). New mononuclear copper(II) complexes from β-diketone and β-keto ester N-donor heterocyclic ligands: Structure, bioactivity, and molecular simulation studies. J. Coord. Chem..

[55] Maghool F., Emami M.H., Alipour R., Mohammadzadeh S., Sereshki N., Dehkordi S.A.E., Fahim A., Tayarani-Najaran Z., Sheikh A., Kesharwani P. (2023). Rescue effect of curcumin against copper toxicity. J. Trace Elem. Med. Biol..

[56] Meza-Morales W., Alvarez-Ricardo Y., Obregón-Mendoza M.A., Arenaza-Corona A., Ramírez-Apan M.T., Toscano R.A., Poveda-Jaramillo J.C., Enríquez R.G. (2023). Three new coordination geometries of homoleptic Zn complexes of curcuminoids and their high antiproliferative potential. RSC Adv..

[57] Zhou S., Li J.B., Yu J., Wang Y.Q., Liu H.Z., Lin G.M., He Z.G., Wang Y.J. (2021). Unique flower-like Cur-metal complexes loaded liposomes for primary and metastatic breast cancer therapy. Mater. Sci. Eng. C.

[58] Qin Q.P., Wei Z.Z., Wang Z.F., Huang X.L., Tan M.X., Zou H.H., Liang H. (2020). Imaging and therapeutic applications of Zn(ii)-cryptolepine-curcumin molecular probes in cell apoptosis detection and photodynamic therapy. Chem. Commun..

[59] Wu R.H., Mei X.T., Ye Y.B., Xue T., Wang J.S., Sun W.J., Lin C.X., Xue R.X., Zhang J.B., Xu D.H. (2019). Zn(II)-curcumin solid dispersion impairs hepatocellular carcinoma growth and enhances chemotherapy by modulating gut microbiota-mediated zinc homeostasis. Pharmacol. Res..

[60] Greish K., Pittala V., Taurin S., Taha S., Bahman F., Mathur A., Jasim A., Mohammed F., El-Deeb I.M., Fredericks S. (2018). Curcumin-Copper Complex Nanoparticles for the Management of Triple-Negative Breast Cancer. Nanomaterials.

[61] Banerjee S., Chakravarty A.R. (2015). Metal Complexes of Curcumin for Cellular Imaging, Targeting, and Photoinduced Anticancer Activity. Acc. Chem. Res..

[62] Goswami T.K., Gadadhar S., Gole B., Karande A.A., Chakravarty A.R. (2013). Photocytotoxicity of copper(II) complexes of curcumin and N-ferrocenylmethyl-L-amino acids. Eur. J. Med. Chem..

[63] Vajragupta O., Boonchoong P., Watanabe H., Tohda M., Kummasud N., Sumanont Y. (2003). Manganese complexes of curcumin and its derivatives: Evaluation for the radical scavenging ability and neuroprotective activity. Free Radic. Biol. Med..

[64] Hema M.K., Karthik C.S., Warad I., Lokanath N.K., Zarrouk A., Kumara K., Pampa K.J., Mallu P. (2018). Regular square planer bis-(4,4,4-trifluoro-1-(thiophen-2-yl)butane-1,3-dione)/copper(II) complex: Trans/cis-DFT isomerization, crystal structure, thermal, solvatochromism, hirshfeld surface and DNA-binding analysis. J. Mol. Struct..

[65] Pramanik A., Laha D., Pramanik P., Karmakar P. (2014). A novel drug copper acetylacetonate loaded in folic acid-tagged chitosan nanoparticle for efficient cancer cell targeting. J. Drug Target..

[66] Xu D.F., Shen Z.H., Shi Y., He Q., Xia Q.C. (2010). Synthesis, characterization, crystal structure, and biological activity of the copper complex. Russ. J. Coord. Chem..

[67] Pellei M., Bagnarelli L., Gabrielli S., Lupidi G., Cimarelli C., Stella F., Dolmella A., Santini C. (2023). Copper(II) complexes based on isopropyl ester derivatives of bis(pyrazol-1-yl)acetate ligands with catalytic potency in organic macro(molecules) synthesis. Inorg. Chim. Acta.

[68] Del Bello F., Pellei M., Bagnarelli L., Santini C., Giorgioni G., Piergentili A., Quaglia W., Battocchio C., Iucci G., Schiesaro I. (2022). Cu(I) and Cu(II) Complexes Based on Lonidamine-Conjugated Ligands Designed to Promote Synergistic Antitumor Effects. Inorg. Chem..

[69] Pellei M., Bagnarelli L., Luciani L., Del Bello F., Giorgioni G., Piergentili A., Quaglia W., De Franco M., Gandin V., Marzano C. (2020). Synthesis and Cytotoxic Activity Evaluation of New Cu(I) Complexes of Bis(pyrazol-1-yl) Acetate Ligands Functionalized with an NMDA Receptor Antagonist. Int. J. Mol. Sci..

[70] Gabrielli S., Pellei M., Venditti I., Fratoddi I., Battocchio C., Iucci G., Schiesaro I., Meneghini C., Palmieri A., Marcantoni E. (2020). Development of new and efficient copper(II) complexes of hexyl bis(pyrazolyl)acetate ligands as catalysts for allylic oxidation. Dalton Trans..

[71] Morelli M.B., Amantini C., Santoni G., Pellei M., Santini C., Cimarelli C., Marcantoni E., Petrini M., Del Bello F., Giorgioni G. (2018). Novel antitumor copper(ii) complexes designed to act through synergistic mechanisms of action, due to the presence of an NMDA receptor ligand and copper in the same chemical entity. New J. Chem..

[72] Gandin V., Ceresa C., Esposito G., Indraccolo S., Porchia M., Tisato F., Santini C., Pellei M., Marzano C. (2017). Therapeutic potential of the phosphino Cu(I) complex (HydroCuP) in the treatment of solid tumors. Sci. Rep..

[73] Tisato F., Marzano C., Peruzzo V., Tegoni M., Giorgetti M., Damjanovic M., Trapananti A., Bagno A., Santini C., Pellei M. (2016). Insights into the cytotoxic activity of the phosphane copper(I) complex Cu(thp)(4) PF6. J. Inorg. Biochem..

[74] Pellei M., Papini G., Trasatti A., Giorgetti M., Tonelli D., Minicucci M., Marzano C., Gandin V., Aquilanti G., Dolmella A. (2011). Nitroimidazole and glucosamine conjugated heteroscorpionate ligands and related copper(II) complexes. Syntheses, biological activity and XAS studies. Dalton Trans..

[75] Papini G., Bandoli G., Dolmella A., Gioia Lobbia G., Pellei M., Santini C. (2008). New homoleptic carbene transfer ligands and related coinage metal complexes. Inorg. Chem. Commun..

[76] Inoue M., Sumii Y., Shibata N. (2020). Contribution of Organofluorine Compounds to Pharmaceuticals. ACS Omega.

[77] Gillis E.P., Eastman K.J., Hill M.D., Donnelly D.J., Meanwell N.A. (2015). Applications of Fluorine in Medicinal Chemistry. J. Med. Chem..

[78] Swallow S., Lawton G., Witty D.R. (2015). Chapter Two—Fluorine in Medicinal Chemistry. Progress in Medicinal Chemistry.

[79] Wang J., Sanchez-Rosello M., Acena J.L., del Pozo C., Sorochinsky A.E., Fustero S., Soloshonok V.A., Liu H. (2014). Fluorine in Pharmaceutical Industry: Fluorine-Containing Drugs Introduced to the Market in the Last Decade (2001–2011). Chem. Rev..

[80] Purser S., Moore P.R., Swallow S., Gouverneur V. (2008). Fluorine in medicinal chemistry. Chem. Soc. Rev..

[81] Shah P., Westwell A.D. (2007). The role of fluorine in medicinal chemistry. J. Enzym. Inhib. Med. Chem..

[82] Isanbor C., O’Hagan D. (2006). Fluorine in medicinal chemistry: A review of anti-cancer agents. J. Fluor. Chem..

[83] Doerrer L.H., Dias H.V.R. (2023). Fluorinated ligands and their effects on physical properties and chemical reactivity. Dalton Trans..

[84] Abdou I.M., Saleh A.M., Zohdi H.F. (2004). Synthesis and antitumor activity of 5-trifluoromethyl-2,4-dihydropyrazol-3- one nucleosides. Molecules.

[85] Edwards P.N., Banks R.E., Smart B.E., Tatlow J.C. (1994). Uses of Fluorine in Chemotherapy. Organofluorine Chemistry: Principles and Commercial Applications.

[86] Lakhi J.S., Patterson M.R., Dias H.V.R. (2020). Coinage metal metallacycles involving a fluorinated 3,5-diarylpyrazolate. New J. Chem..

[87] Zhang C., Yang P., Yang Y., Huang X., Yang X.-J., Wu B. (2008). High-Yield Synthesis of 1,3-Dimesityl-propane-1,3-dione: Isolation of Its Aluminum Complex as a Stable Intermediate. Synth. Commun..

[88] Pellei M., Del Gobbo J., Caviglia M., Karade D.V., Gandin V., Marzano C., Noonikara Poyil A., Dias H.V.R., Santini C. (2023). Synthesis and cytotoxicity studies of Cu(I) and Ag(I) complexes based on sterically hindered β-diketonates with different degrees of fluorination. Dalton Trans..

[89] Crowder J.M., Han H.X., Wei Z., Dikarev E.V., Petrukhina M.A. (2019). Unsolvated homo- and heterometallic highly fluorinated β-diketonate complexes of copper(II). Polyhedron.

[90] Larson A.T., Crossman A.S., Krajewski S.M., Marshak M.P. (2020). Copper(II) as a Platform for Probing the Steric Demand of Bulky beta-Diketonates. Inorg. Chem..

[91] He C., Zhang G., Ke J., Zhang H., Miller J.T., Kropf A.J., Lei A. (2013). Labile Cu(I) Catalyst/Spectator Cu(II) Species in Copper-Catalyzed C–C Coupling Reaction: Operando IR, in Situ XANES/EXAFS Evidence and Kinetic Investigations. J. Am. Chem. Soc..

[92] Groom C.R., Bruno I.J., Lightfoot M.P., Ward S.C. (2016). The Cambridge Structural Database. Acta Crystallogr. Sect. B Struct. Sci. Cryst. Eng. Mater..

[93] Layek S., Kumari S., Anuradha, Agrahari B., Ganguly R., Pathak D.D. (2016). Synthesis, characterization and crystal structure of a diketone based Cu(II) complex and its catalytic activity for the synthesis of 1,2,3-triazoles. Inorg. Chim. Acta.

[94] Soldatov D.V., Henegouwen A.T., Enright G.D., Ratcliffe C.I., Ripmeester J.A. (2001). Nickel(II) and zinc(II) dibenzoylmethanates: Molecular and crystal structure, polymorphism, and guest- or temperature-induced oligomerization. Inorg. Chem..

[95] Abbati G.L., Cornia A., Fabretti A.C., Caneschi A., Gatteschi D. (1998). Structure and Magnetic Properties of a Mixed-Valence Heptanuclear Manganese Cluster. Inorg. Chem..

[96] Takashima Y., Hanamura T., Maeda Y. (1970). The Mössbauer spectra of iron-dibenzoylmethane complexes. J. Inorg. Nucl. Chem..

[97] Lu H.J., Gao J., Du C.X., Fan Y.T., Hou H.W., Ding D.G., Zhai J.L. (2003). Cobalt (II) complexes of dibenzoylmethane (Hdbm): Crystal structures and axial metathetical reaction of the complexes with pyridine or its derivatives. Chin. J. Inorg. Chem..

[98] Ma B.Q., Gao S., Wang Z.M., Liao C.S., Yan C.H., Xu G.X. (1999). Synthesis and structure of bis(dibenzoylmethane) copper(II). J. Chem. Crystallogr..

[99] Knyazeva A.N., Shugam E.A., Shkol’nikova L.M. (1969). Crystal chemical data on inner complexes of β-diketones—IV. Crystal and molecular structure of copper dibenzoylmethanate. J. Mol. Struct..

[100] Krause L., Herbst-Irmer R., Sheldrick G.M., Stalke D. (2015). Comparison of silver and molybdenum microfocus X-ray sources for single-crystal structure determination. J. Appl. Cryst..

[101] Sheldrick G.M. (2015). SHELXT—Integrated space-group and crystal-structure determination. Acta Crystallogr. Sect. A Found. Adv..

[102] Sheldrick G.M. (2015). Crystal structure refinement with SHELXL. Acta Crystallogr. Sect. C Struct. Chem..

[103] Dolomanov O.V., Bourhis L.J., Gildea R.J., Howard J.A.K., Puschmann H. (2009). OLEX2: A complete structure solution, refinement and analysis program. J. Appl. Crystallogr..

[104] Pellei M., Santini C., Bagnarelli L., Caviglia M., Sgarbossa P., De Franco M., Zancato M., Marzano C., Gandin V. (2023). Novel Silver Complexes Based on Phosphanes and Ester Derivatives of Bis(pyrazol-1-yl)acetate Ligands Targeting TrxR: New Promising Chemotherapeutic Tools Relevant to SCLC Management. Int. J. Mol. Sci..

