# Supplementary material for: Synthesis and Investigations of the Antitumor Effects of First-Row Transition Metal(II) Complexes Supported by Two Fluorinated and Non-Fluorinated β-Diketonates
Source: Int J Mol Sci. 2024 Feb 7;25(4):2038. doi: 10.3390/ijms25042038 (PMC10889438; doi:10.3390/ijms25042038)
Supplement: Supplementary file 1 [file ijms-25-02038-s001.zip › ijms-2854998-supplementary.pdf]

Supporting Information

Synthesis and Investigations of the Antitumor Effects of First-Row Transition Metal(II) Complexes Supported by Two Fluorinated and Non-Fluorinated β-Diketonates

Maura Pellei [1,*], Jo' Del Gobbo [1], Miriam Caviglia [1], Valentina Gandin [2], Cristina Marzano [2,*], Deepika V. Karade [3], Anurag Noonikara Poyil [3], H. V. Rasika Dias [3] and Carlo Santini [1]

1 *School of Science and Technology—Chemistry Division, University of Camerino, Via Madonna delle Carceri (ChIP), Camerino, 62032 Macerata, Italy*
2 *Department of Pharmaceutical and Pharmacological Sciences, University of Padova, Via Marzolo 5, 35131 Padova, Italy*
3 *Department of Chemistry and Biochemistry, The University of Texas at Arlington, P.O. Box 19065, Arlington, TX 76019, USA*
* *Correspondence: maura.pellei@unicam.it (M.P.); cristina.marzano@unipd.it (C.M.)*

Table of Contents:

Figure S1. Two views of [Cu(L^{Mes})$_2$] (**11**).

Figure S2. Two views of [Zn(L^{Mes})$_2$] (**12**).

Figure S3. A view of [Cu(L^{CF3})$_2$(THF)]·2(THF) showing THF and CF_3 moiety disorder.

Figure S4. FT-IR spectrum of NaL^{CF3}.

Figure S5. ^{1}H-NMR spectrum of NaL^{CF3} in DMSO-d$_6$.

Figure S6. ^{13}C{^{1}H}-NMR spectrum of NaL^{CF3} in DMSO-d$_6$.

Figure S7. ^{19}F{^{1}H}-NMR spectrum of NaL^{CF3} in DMSO-d$_6$.

Figure S8. ESI-MS(+) spectrum of NaL^{CF3} in CH_3CN.

Figure S9. FT-IR spectrum of NaL^{Mes}.

Figure S10. ^{1}H-NMR spectrum of NaL^{Mes} in $CDCl_3$.

Figure S11. ^{13}C{^{1}H}-NMR of NaL^{Mes} in $CDCl_3$.

Figure S12. ESI-MS(+) spectrum of NaL^{Mes} in CH_3OH.

Figure S13. FT-IR spectrum of NaL^{Ph}.

Figure S14. ^{1}H-NMR of NaL^{Ph} in Acetone-d$_6$.

Figure S15. ^{13}C{^{1}H}-NMR of NaL^{Ph} in Acetone-d$_6$.

Figure S16. ESI-MS(+) spectrum of NaL^{Ph} in CH_3OH.

Figure S17. FT-IR spectrum of [Mn(L^{CF3})$_2$(H_2O)$_2$] (**1**).

Figure S18. ESI-MS(+) spectrum of [Mn(L^{CF3})$_2$(H_2O)$_2$] (**1**) in CH_3OH/CH_3CN.

Figure S19. FT-IR spectrum of [Fe(L^{CF3})$_2$] (**2**).

Figure S20. ESI-MS(+) spectrum of [Fe(L^{CF3})$_2$] (**2**) in CH_3CN.

Figure S21. FT-IR spectrum of [Co(L^{CF3})$_2$(H_2O)$_2$] (**3**).

Figure S22. ESI-MS(+) spectrum of [Co(L^{CF3})$_2$(H_2O)$_2$] (**3**) in CH_3OH/CH_3CN.

Figure S23. FT-IR spectrum of [Ni(L^{CF3})$_2$(H_2O)$_2$] (**4**).

Figure S24. ESI-MS(+) spectrum of [Ni(L^{CF3})$_2$(H_2O)$_2$] (**4**) in CH_3OH/CH_3CN.
Figure S25. FT-IR spectrum of [Cu(L^{CF3})$_2$] (**5**).
Figure S26. ESI-MS(+) spectrum of [Cu(L^{CF3})$_2$] (**5**) in CH_3OH/CH_3CN.
Figure S27. FT-IR spectrum of [Zn(L^{CF3})$_2$] (**6**).
Figure S28. ^{1}H-NMR spectrum of [Zn(L^{CF3})$_2$] (**6**) in Acetone-d_6.
Figure S29. ^{13}C{^{1}H}-NMR spectrum of [Zn(L^{CF3})$_2$] (**6**) in Acetone-d_6.
Figure S30. ^{19}F{^{1}H}-NMR spectrum of [Zn(L^{CF3})$_2$] (**6**) in Acetone-d_6.
Figure S31. FT-IR spectrum of [Mn(L^{Mes})$_2$(H_2O)$_2$] (**7**).
Figure S32. ESI-MS(+) spectrum of [Mn(L^{Mes})$_2$(H_2O)$_2$] (**7**) in CH_3OH.
Figure S33. FT-IR spectrum of [Fe(L^{Mes})$_2$] (**8**).
Figure S34. ESI-MS(+) spectrum of [Fe(L^{Mes})$_2$] (**8**) in CH_3CN.
Figure S35. FT-IR spectrum of [Co(L^{Mes})$_2$(H_2O)$_2$] (**9**).
Figure S36. ESI-MS(+) spectrum of [Co(L^{Mes})$_2$(H_2O)$_2$] (**9**) in CH_3OH/CH_3CN.
Figure S37. FT-IR spectrum of [Ni(L^{Mes})$_2$(H_2O)$_2$] (**10**).
Figure S38. ESI-MS(+) spectrum of [Ni(L^{Mes})$_2$(H_2O)$_2$] (**10**) in CH_3CN.
Figure S39. FT-IR spectrum of [Cu(L^{Mes})$_2$] (**11**).
Figure S40. ESI-MS(+) spectrum of [Cu(L^{Mes})$_2$] (**11**) in CH_3OH.
Figure S41. FT-IR spectrum of [Zn(L^{Mes})$_2$] (**12**).
Figure S42. ^{1}H-NMR spectrum of [Zn(L^{Mes})$_2$] (**12**) in $CDCl_3$.
Figure S43. ^{13}C{^{1}H}-NMR spectrum of [Zn(L^{Mes})$_2$] (**12**) in $CDCl_3$.
Figure S44. FT-IR spectrum of [Mn(L^{Ph})$_2$(H_2O)$_2$] (**13**).
Figure S45. ESI-MS(+) spectrum of [Mn(L^{Ph})$_2$(H_2O)$_2$] (**13**) in CH_3OH.
Figure S46. FT-IR spectrum of [Fe(L^{Ph})$_2$] (**14**).
Figure S47. ESI-MS(+) spectrum of [Fe(L^{Ph})$_2$] (**14**) in CH_3OH/CH_3CN.
Figure S48. FT-IR spectrum of [Co(L^{Ph})$_2$(H_2O)$_2$] (**15**).
Figure S49. ESI-MS(+) spectrum of [Co(L^{Ph})$_2$(H_2O)$_2$] (**15**) in CH_3CN.
Figure S50. FT-IR spectrum of [Ni(L^{Ph})$_2$(H_2O)$_2$] (**16**).
Figure S51. ESI-MS(+) spectrum of [Ni(L^{Ph})$_2$(H_2O)$_2$] (**16**) in CH_3CN.
Figure S52. FT-IR spectrum of [Cu(L^{Ph})$_2$] (**17**).
Figure S53. ESI-MS(+) spectrum of [Cu(L^{Ph})$_2$] (**17**) in CH_3OH.
Figure S54. FT-IR spectrum of [Zn(L^{Ph})$_2$]·2H_2O (**18**).
Figure S55. ^{1}H-NMR spectrum of [Zn(L^{Ph})$_2$]·2H_2O (**18**) in $CDCl_3$.
Figure S56. ^{13}C{^{1}H}-NMR spectrum of [Zn(L^{Ph})$_2$]·2H_2O (**18**) in $CDCl_3$.
Figure S57. Stability studies. All complexes were dissolved at 50 μM in 0.5% DMSO/ physiological solution. UV-Visible spectra were recorded at t = 0 h and t = 72 h.

Table S1. Crystal data and structure refinement for [Cu(L^{Mes})$_2$] (**11**).

Table S2. Bond Lengths for [Cu(L^{Mes})$_2$] (**11**).

Table S3. Bond Angles for [Cu(L^{Mes})$_2$] (**11**).

Table S4. Crystal data and structure refinement for [Zn(L^{Mes})$_2$] (**12**).

Table S5. Bond Lengths for [Zn(L^{Mes})$_2$] (**12**).

Table S6. Bond Angles for [Zn(L^{Mes})$_2$] (**12**).

Table S7. Crystal data and structure refinement for [Cu(L^{CF3})$_2$(THF)]·2(THF).

Table S8. Bond Lengths for [Cu(L^{CF3})$_2$(THF)]·2(THF).

Table S9. Bond Angles for [Cu(L^{CF3})$_2$(THF)]·2(THF).

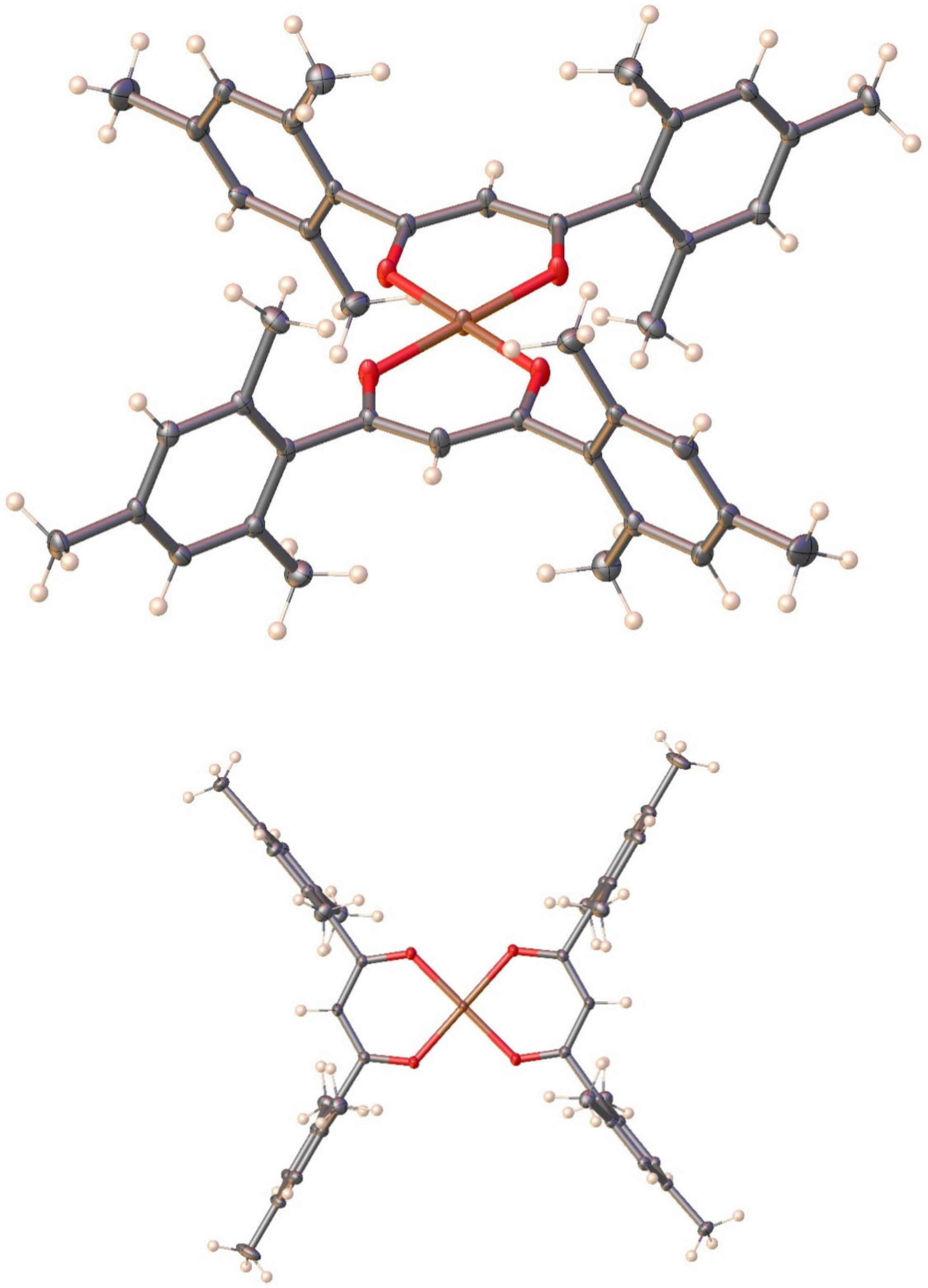

Figure S1. Two views of [Cu(L^{Mes})$_2$] (**11**).

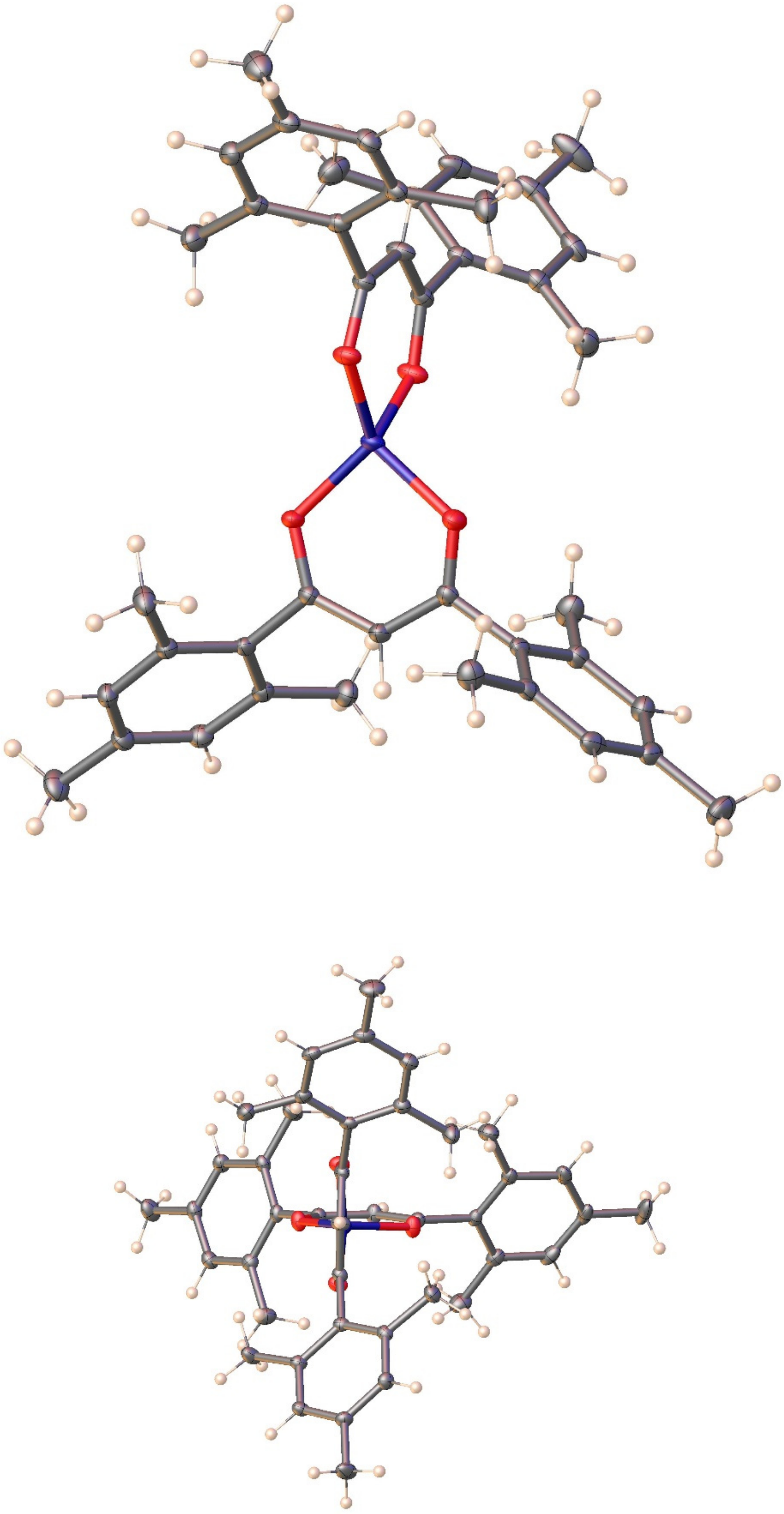

Figure S2. Two views of $[Zn(L^{Mes})_2]$ (**12**).

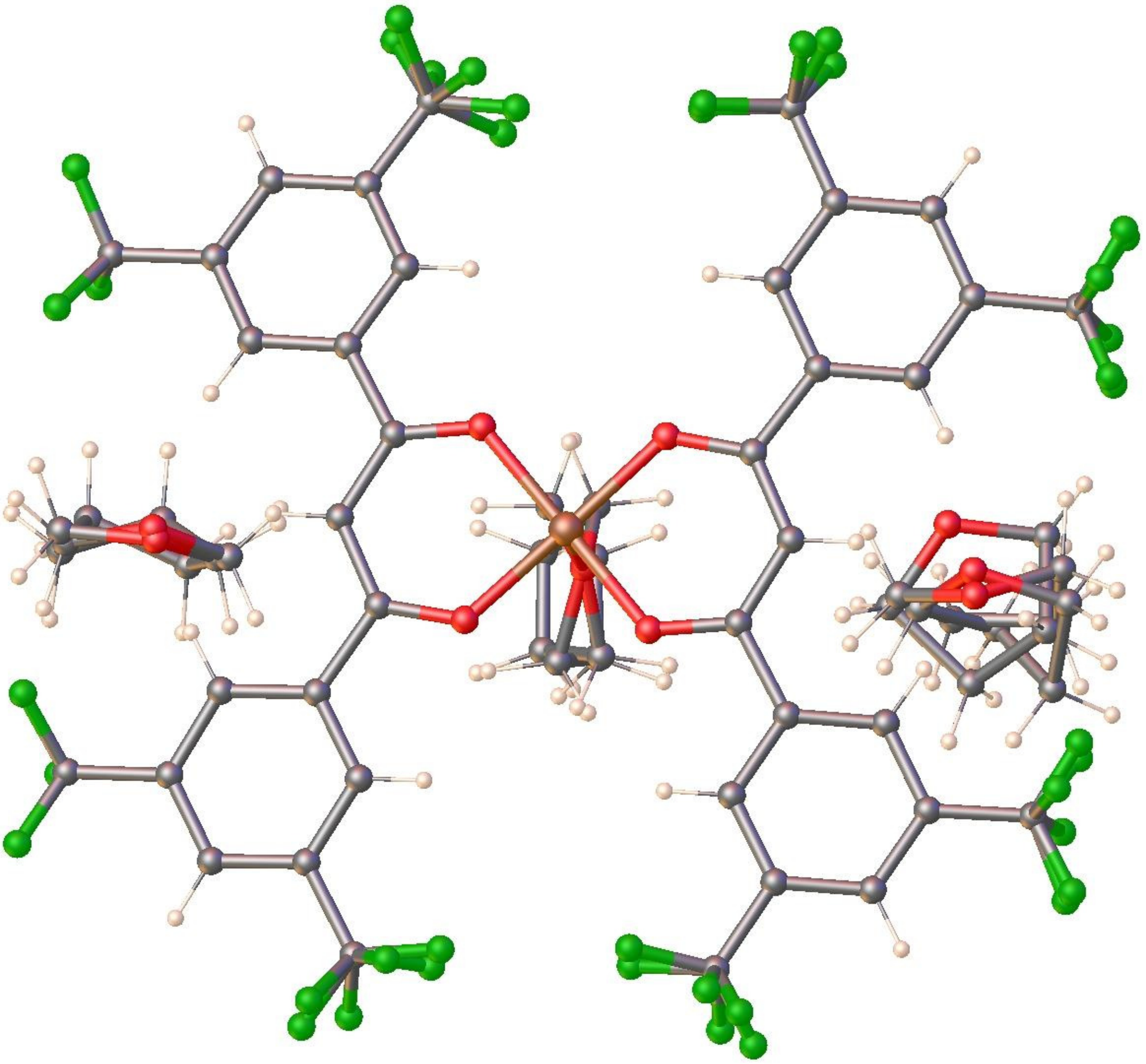

Figure S3. A view of $[Cu(L^{CF3})_2(THF)]{\cdot}2(THF)$ showing THF and CF_3 moiety disorder.

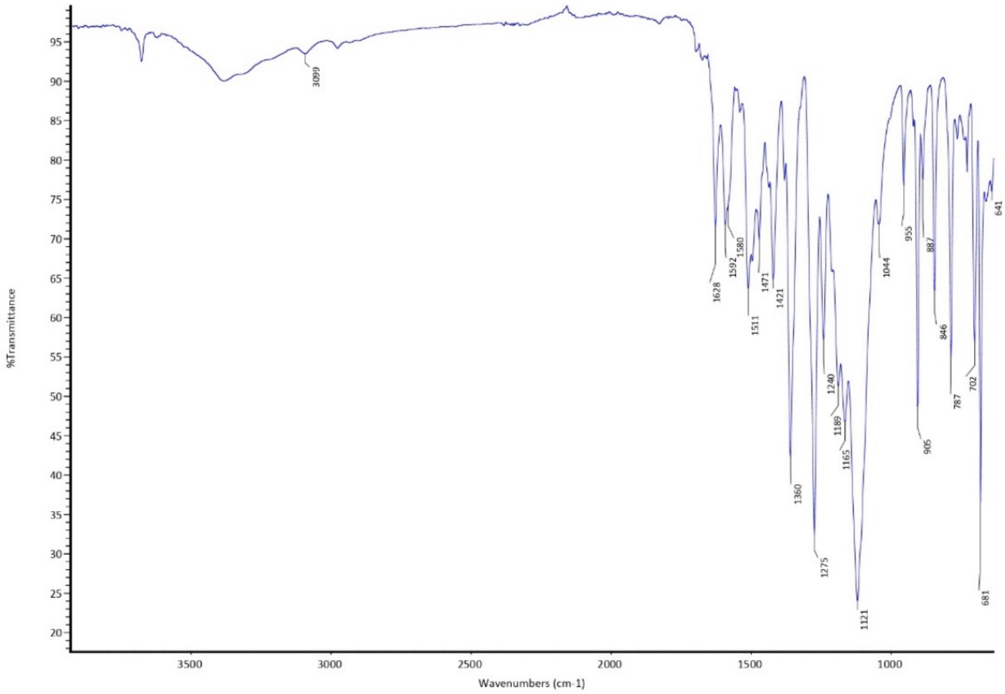

Figure S4. FT-IR spectrum of NaL^{CF3}.

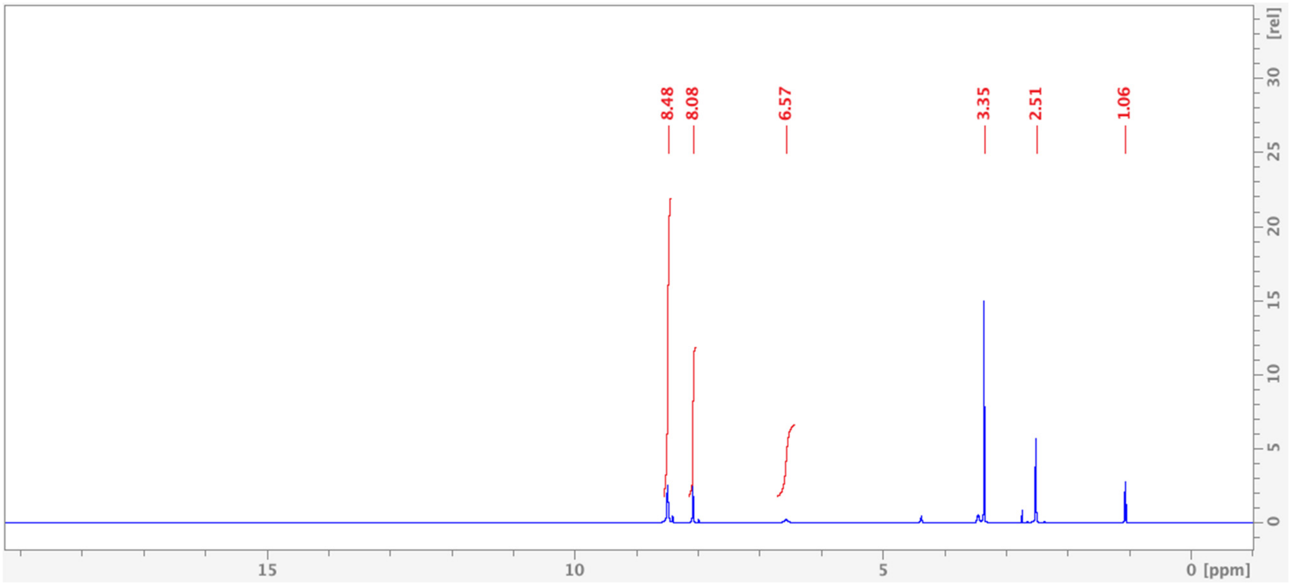

Figure S5. ^{1}H-NMR spectrum of NaL^{CF3} in DMSO-d_6.

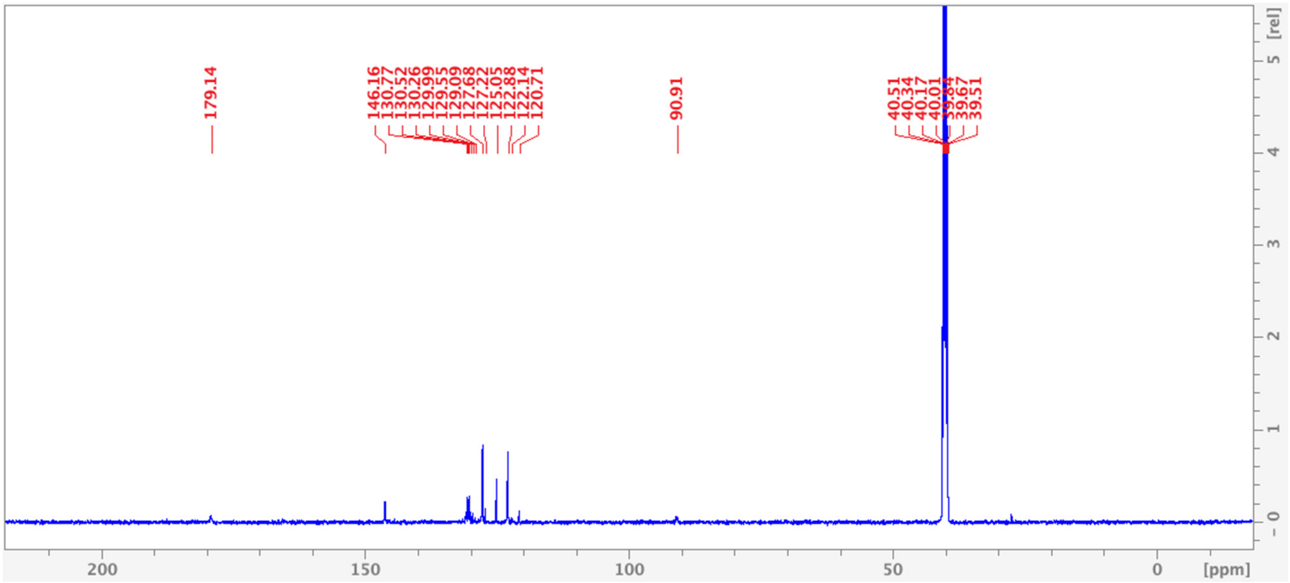

Figure S6. $^{13}C\{^1H\}$-NMR spectrum of NaL^{CF3} in DMSO-d_6.

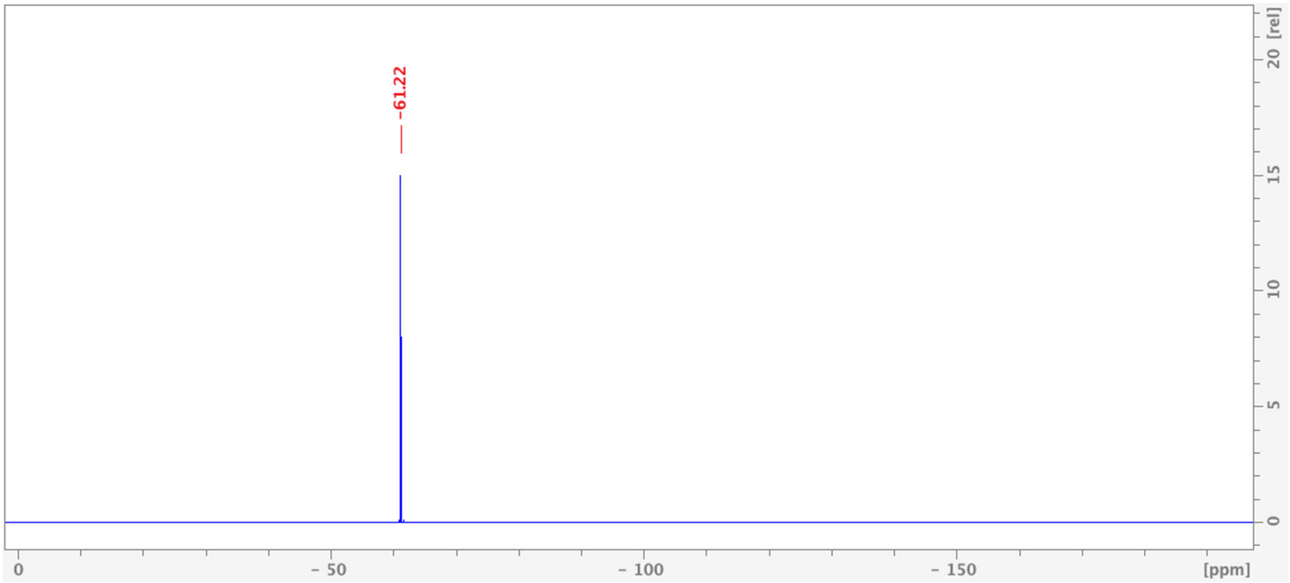

Figure S7. $^{19}F\{^1H\}$-NMR spectrum of NaL^{CF3} in DMSO-d_6.

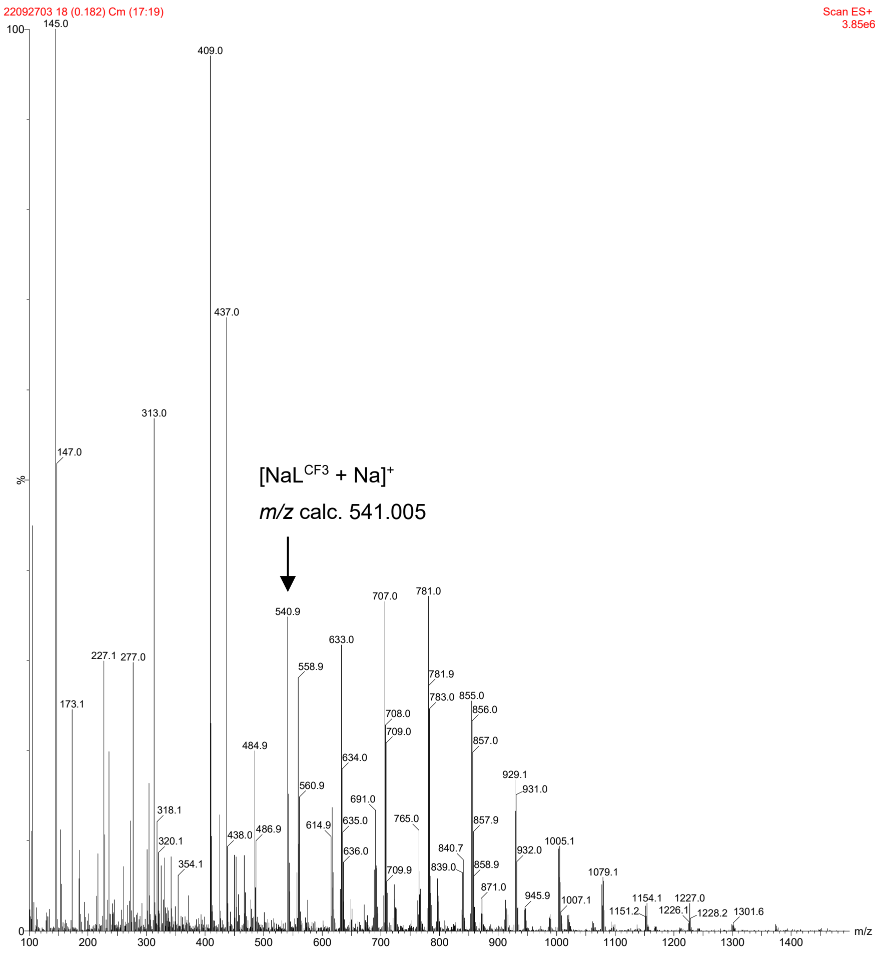

Figure S8. ESI-MS(+) spectrum of NaL^{CF3} in CH_3CN.

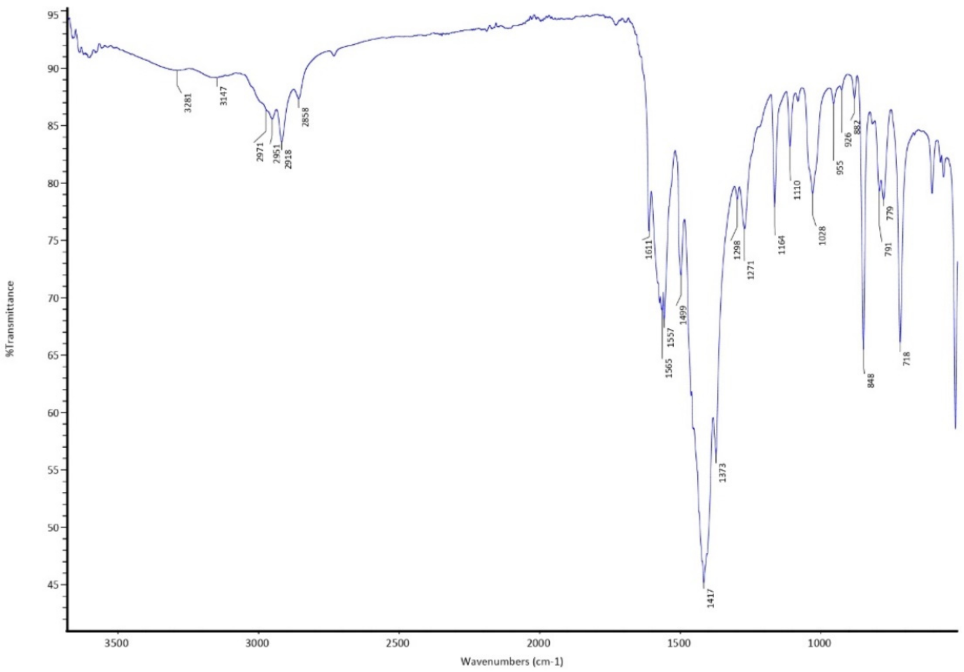

Figure S9. FT-IR spectrum of NaL^{Mes}.

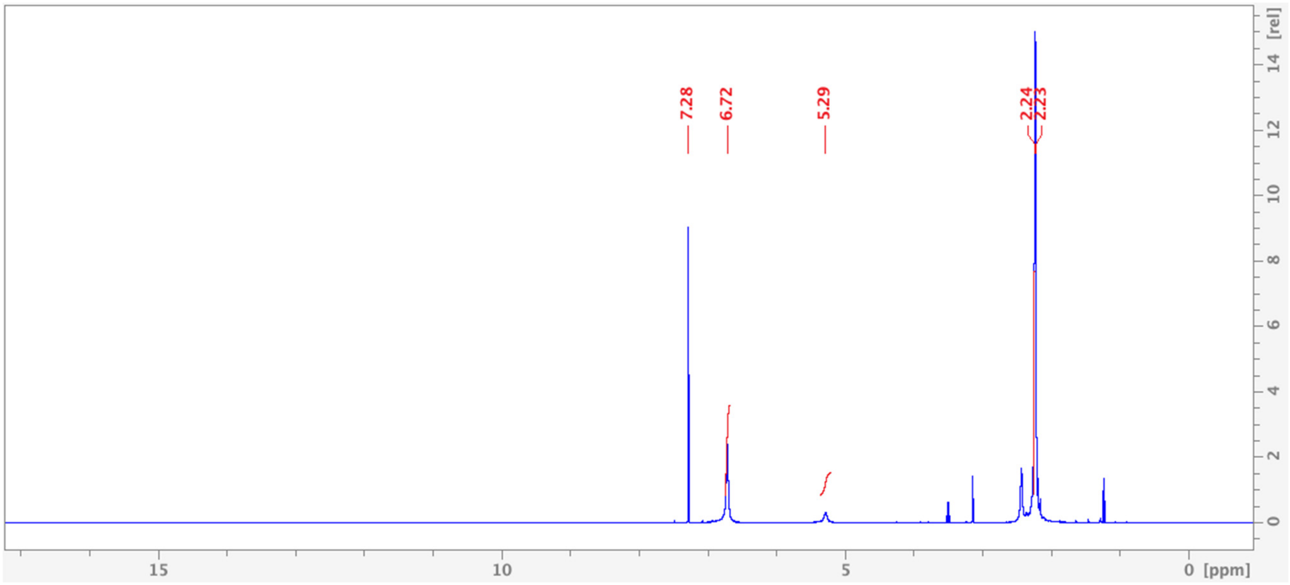

Figure S10. ^{1}H-NMR spectrum of NaL^{Mes} in $CDCl_3$.

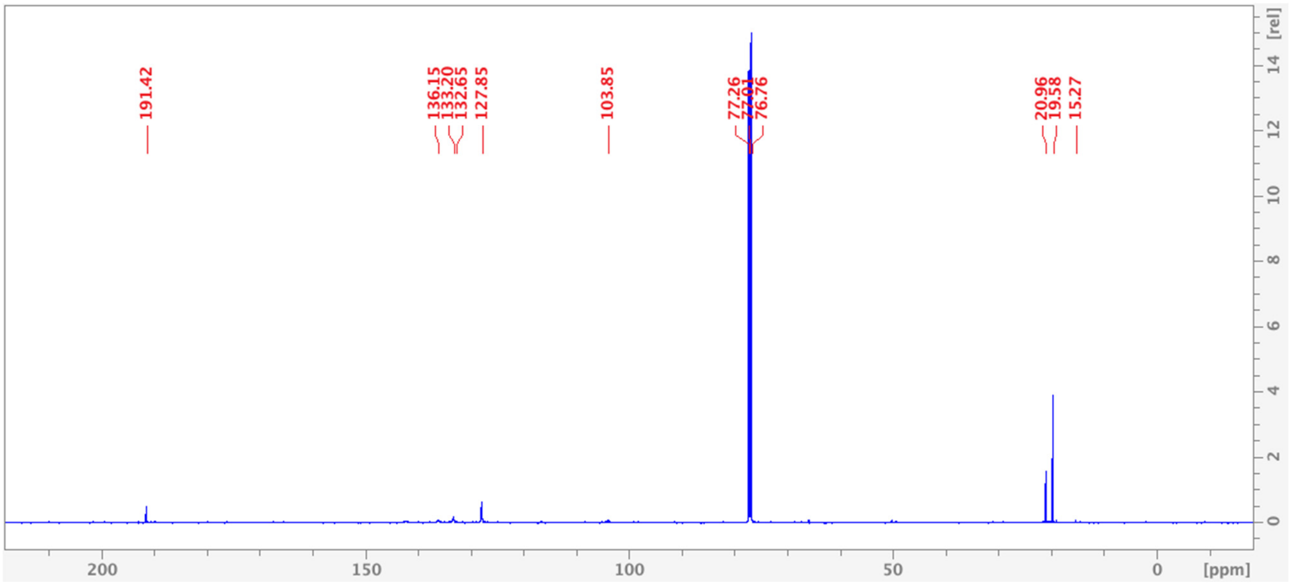

Figure S11. $^{13}C\{^{1}H\}$-NMR of NaL^{Mes} in $CDCl_3$.

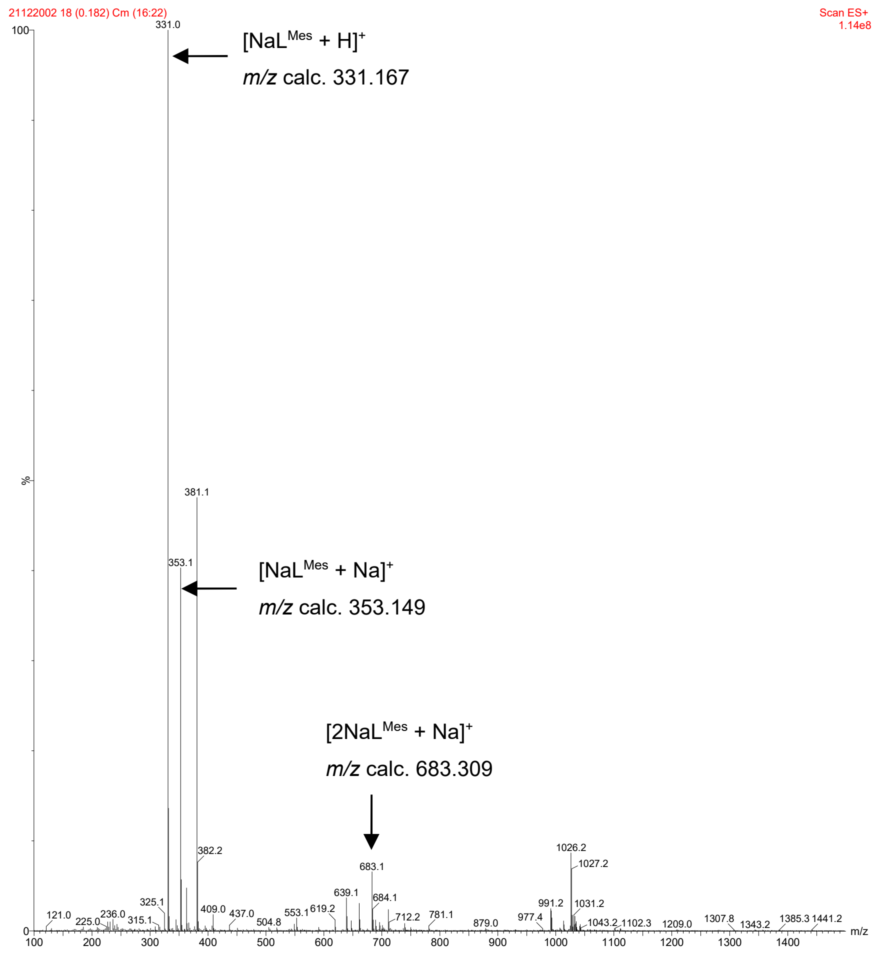

Figure S12. ESI-MS(+) spectrum of NaL^{Mes} in CH_3OH.

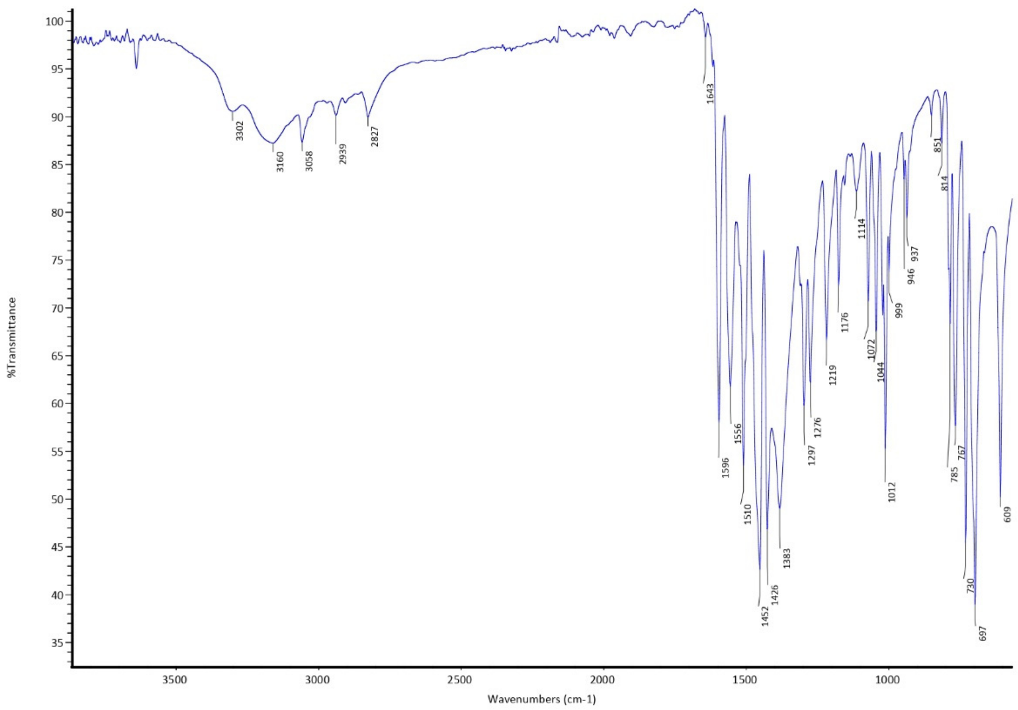

Figure S13. FT-IR spectrum of NaL^{Ph}.

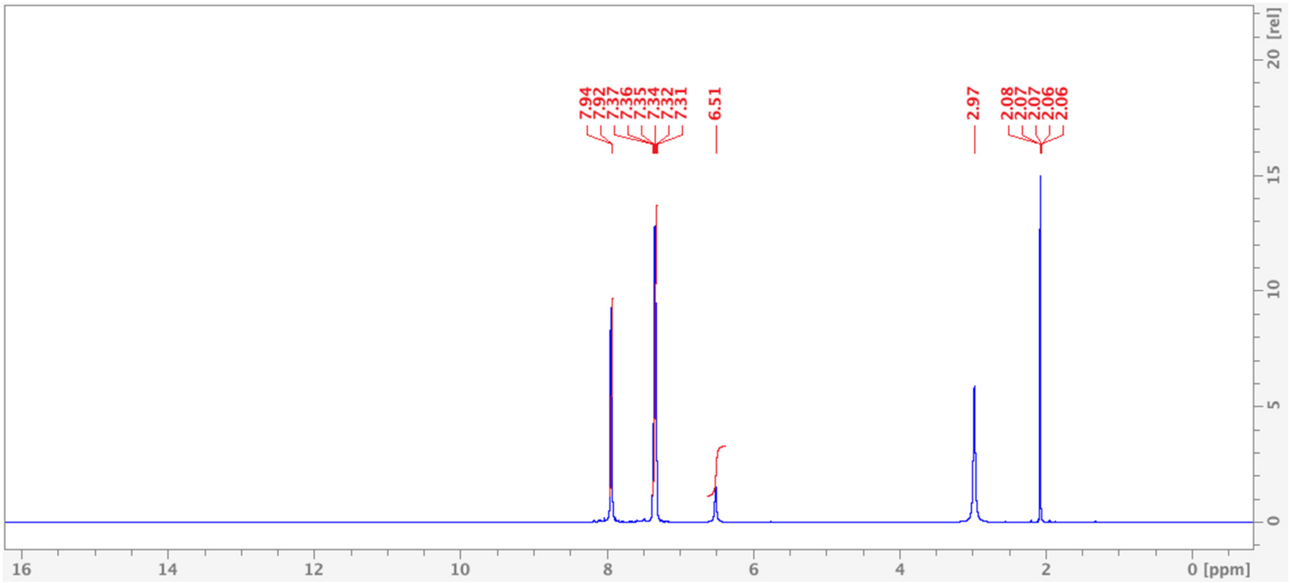

Figure S14. ^{1}H-NMR of NaL^{Ph} in Acetone-d_6.

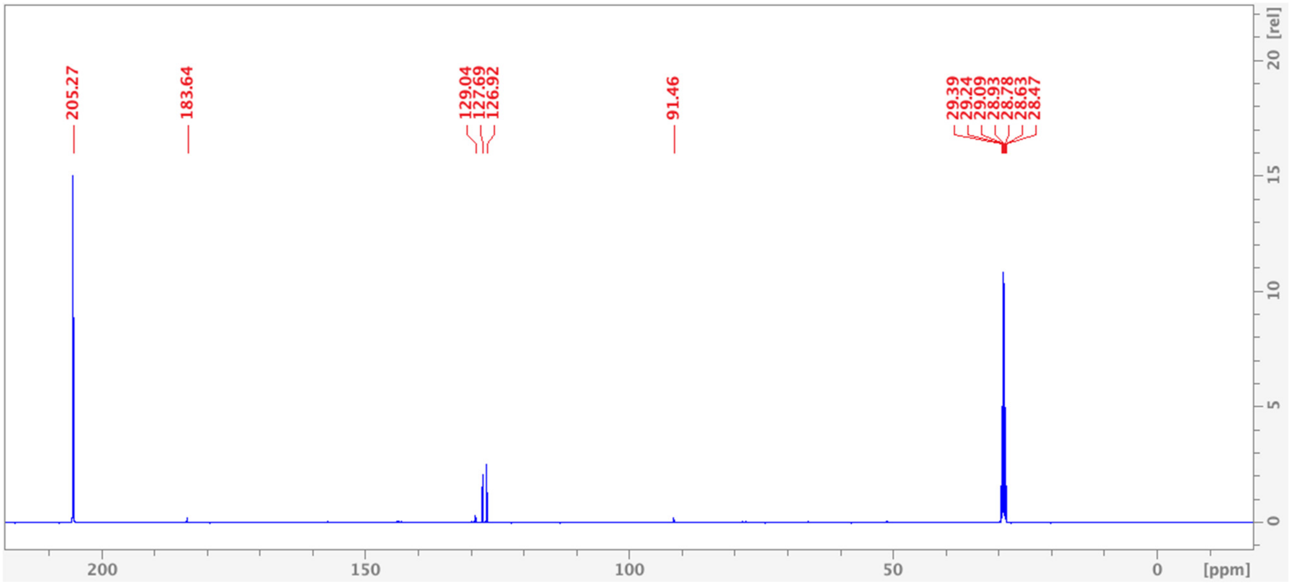

Figure S15. $^{13}C\{^{1}H\}$-NMR of NaL^{Ph} in Acetone-d_6.

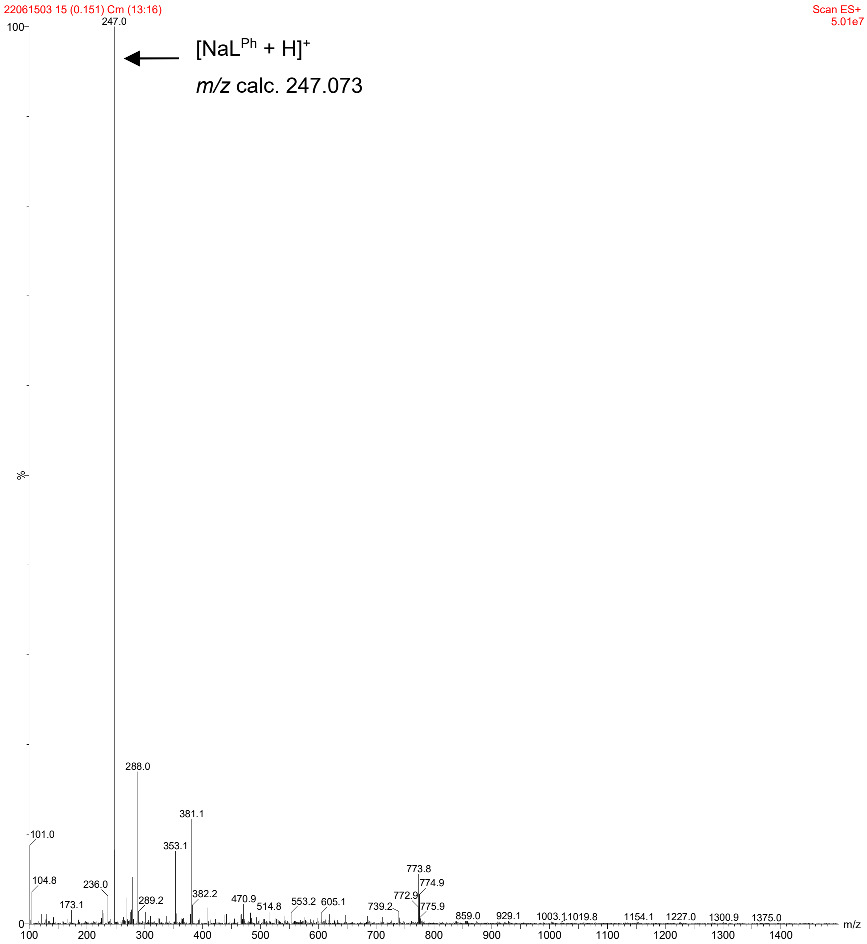

Figure S16. ESI-MS(+) spectrum of NaL^{Ph} in CH_3OH.

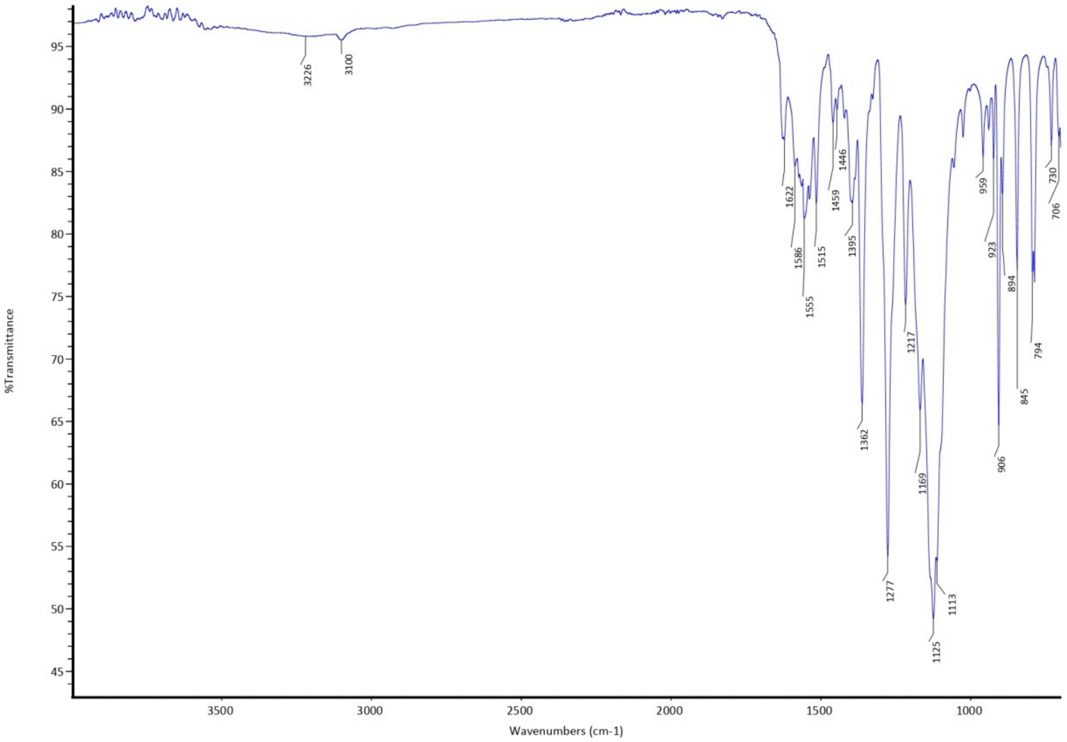

Figure S17. FT-IR spectrum of [Mn(L^{CF3})$_2$(H_2O)$_2$] (**1**).

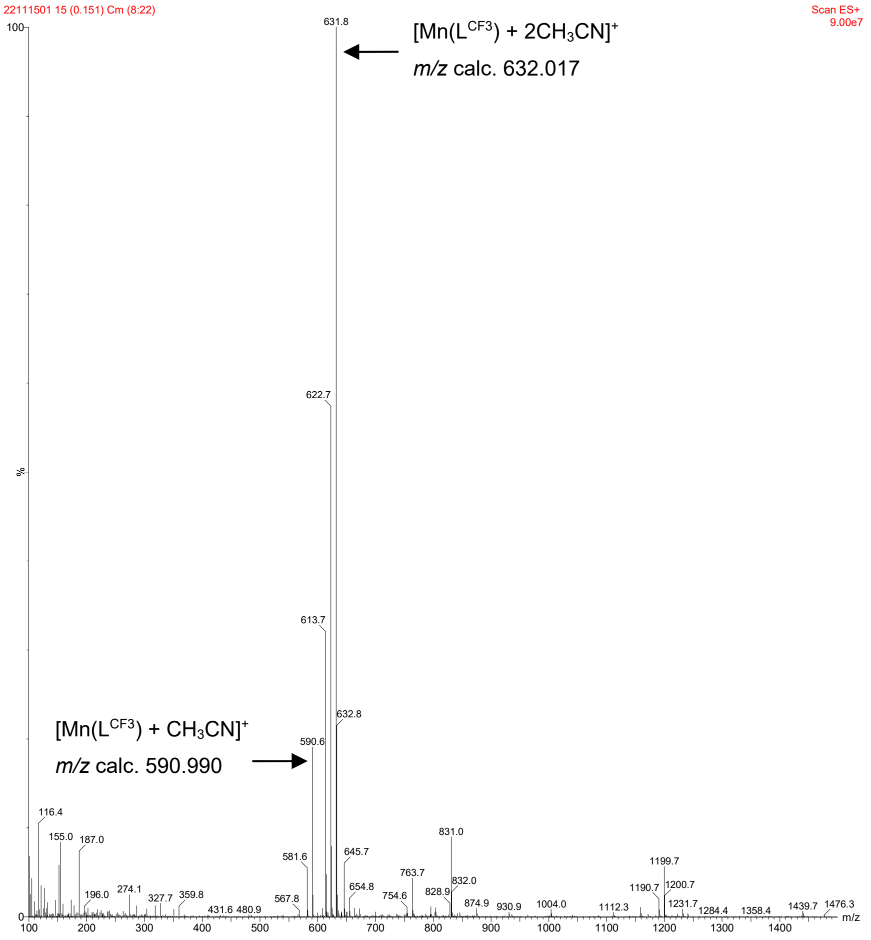

Figure S18. ESI-MS(+) spectrum of $[Mn(L^{CF3})_2(H_2O)_2]$ (**1**) in CH_3OH/CH_3CN.

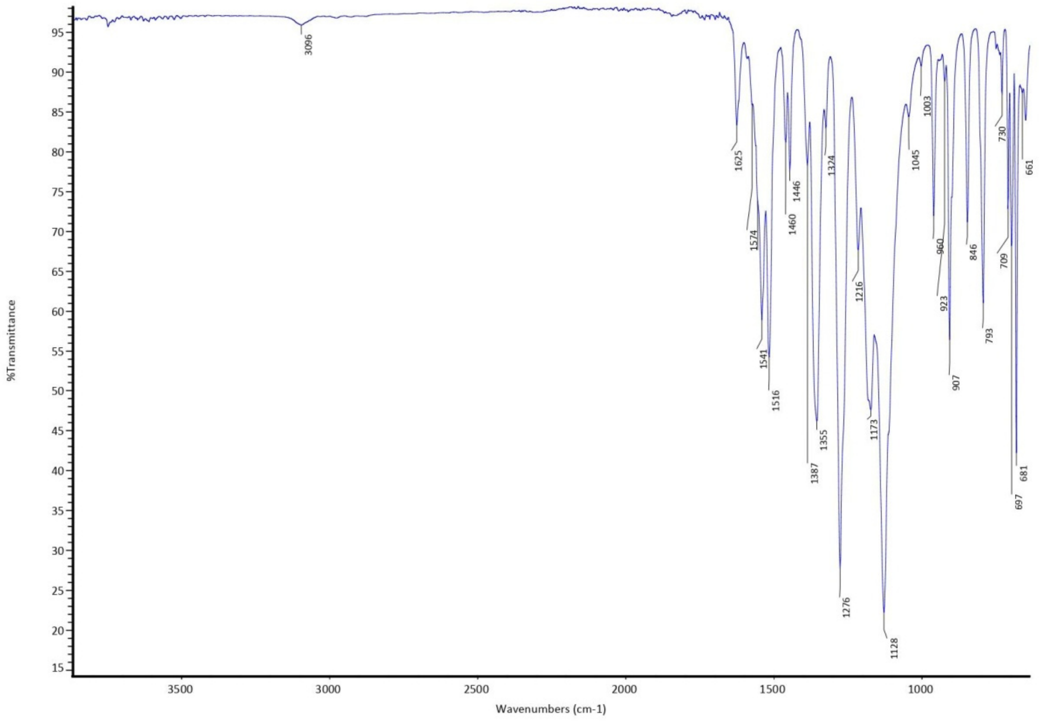

Figure S19. FT-IR spectrum of $[Fe(L^{CF3})_2]$ (**2**).

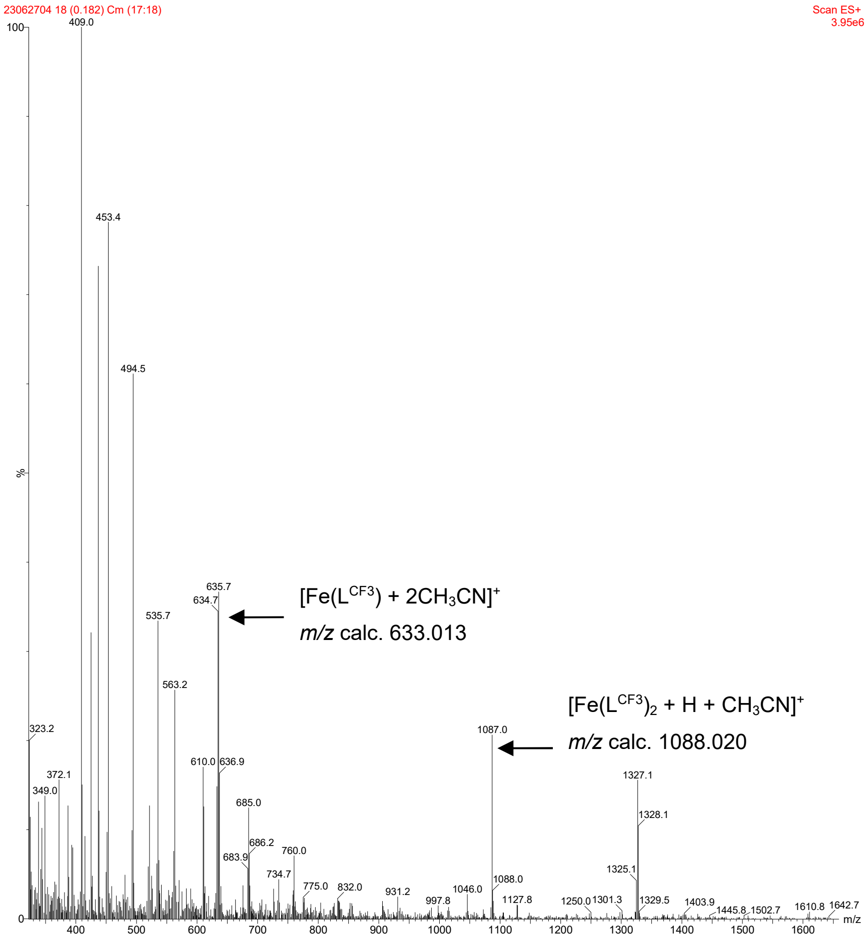

Figure S20. ESI-MS(+) spectrum of $[Fe(L^{CF3})_2]$ (**2**) in CH_3CN.

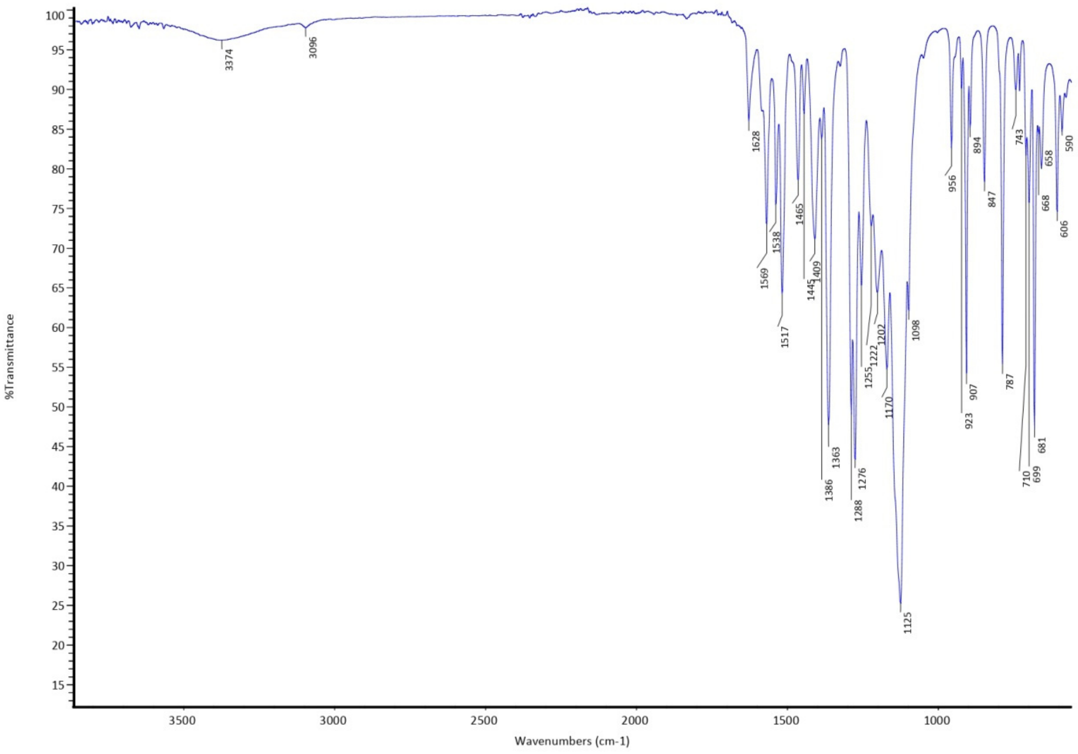

Figure S21. FT-IR spectrum of $[Co(L^{CF3})_2(H_2O)_2]$ (**3**).

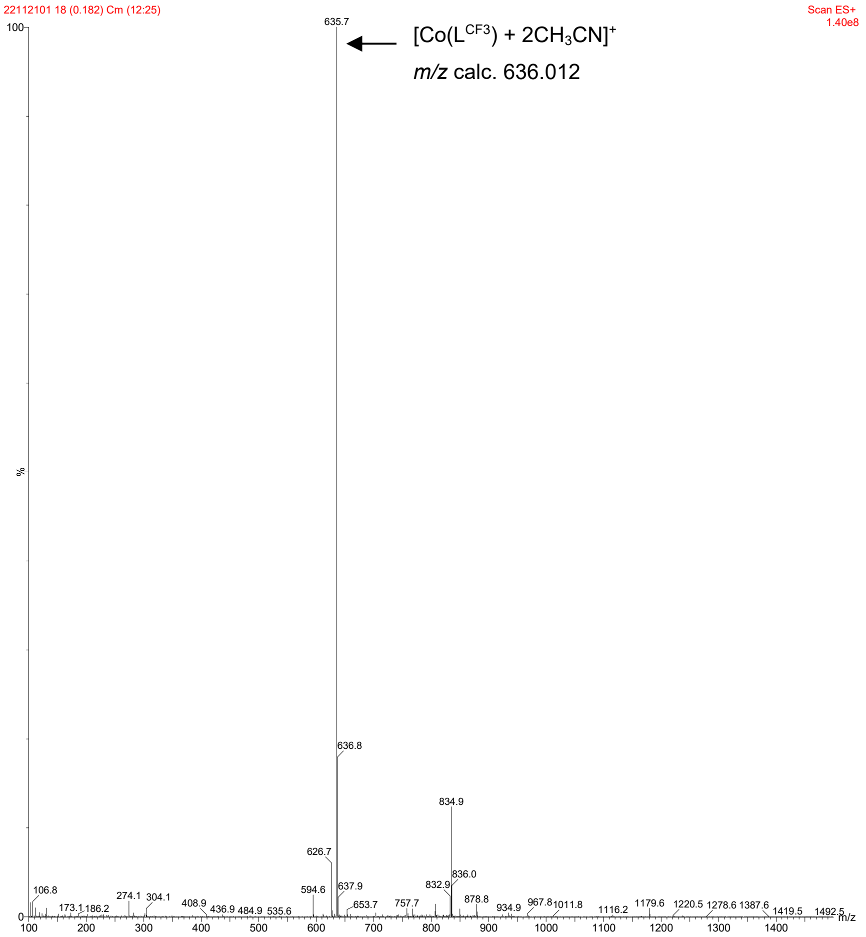

Figure S22. ESI-MS(+) spectrum of [Co(L^{CF3})$_2$(H_2O)$_2$] (**3**) in CH_3OH/CH_3CN**.**

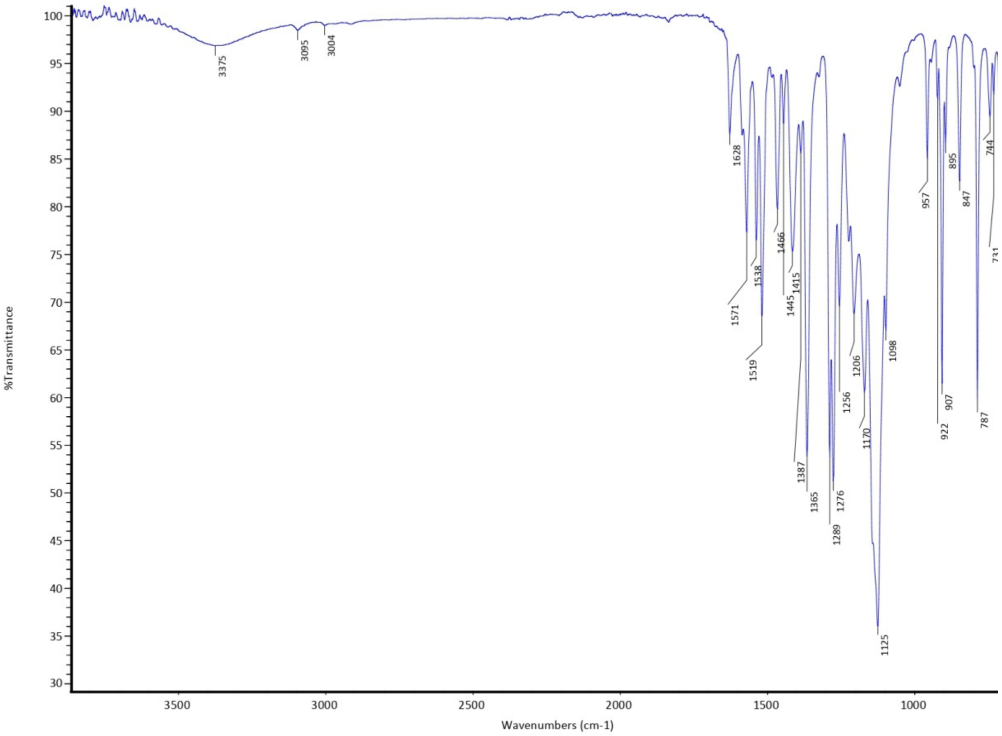

Figure S23. FT-IR spectrum of [Ni(L^{CF3})$_2$(H_2O)$_2$] (**4**).

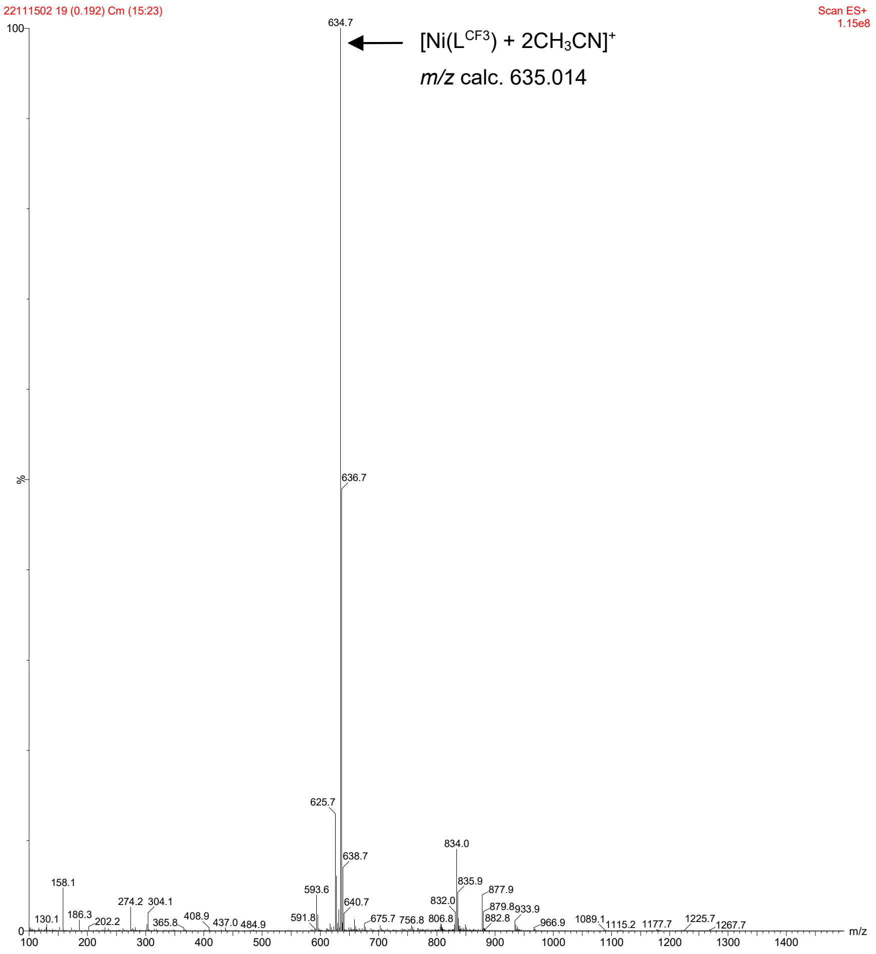

Figure S24. ESI-MS(+) spectrum of $[Ni(L^{CF3})_2(H_2O)_2]$ (**4**) in CH_3OH/CH_3CN.

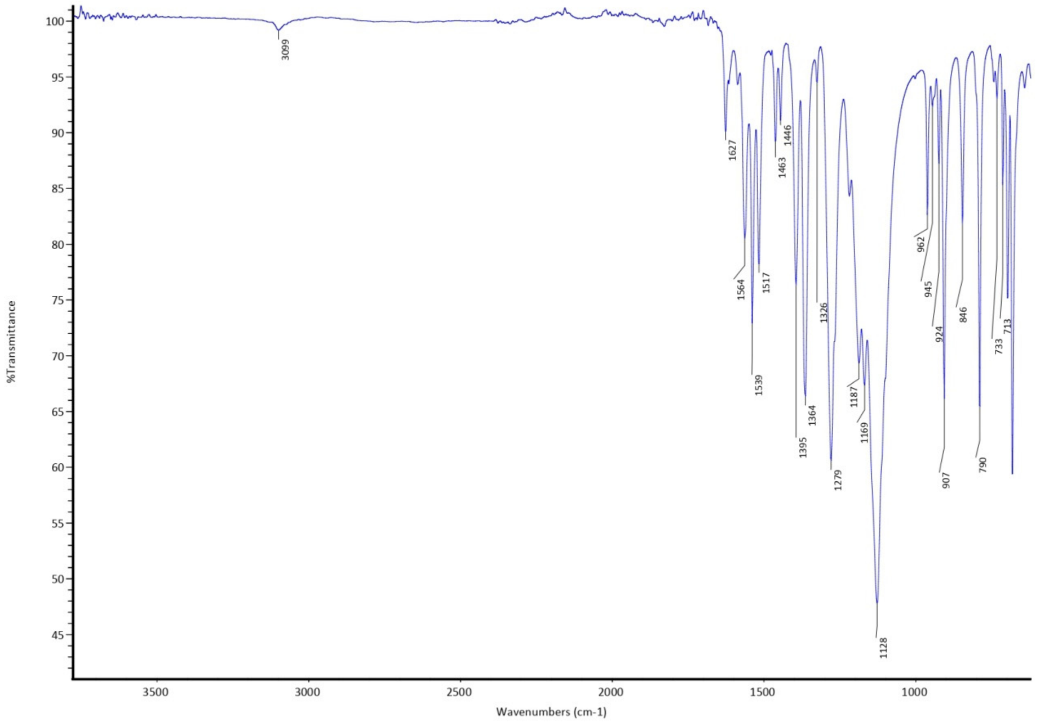

Figure S25. FT-IR spectrum of $[Cu(L^{CF3})_2]$ (**5**).

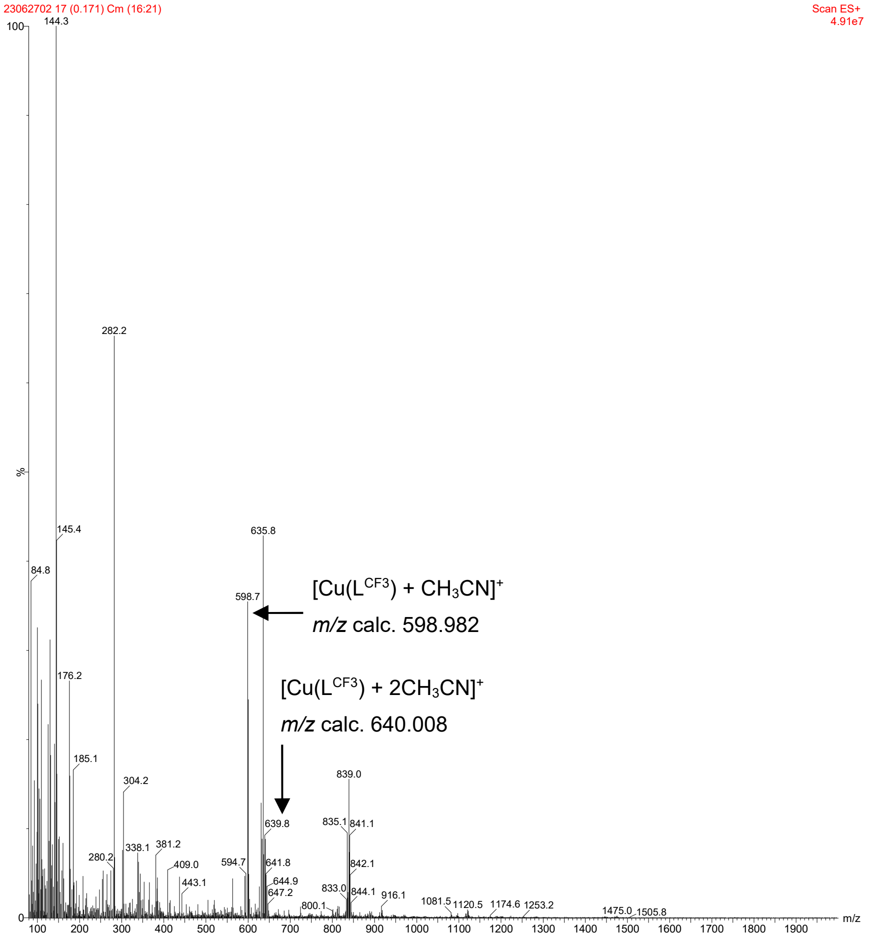

Figure S26. ESI-MS(+) spectrum of $[Cu(L^{CF3})_2]$ (**5**) in CH_3OH/CH_3CN.

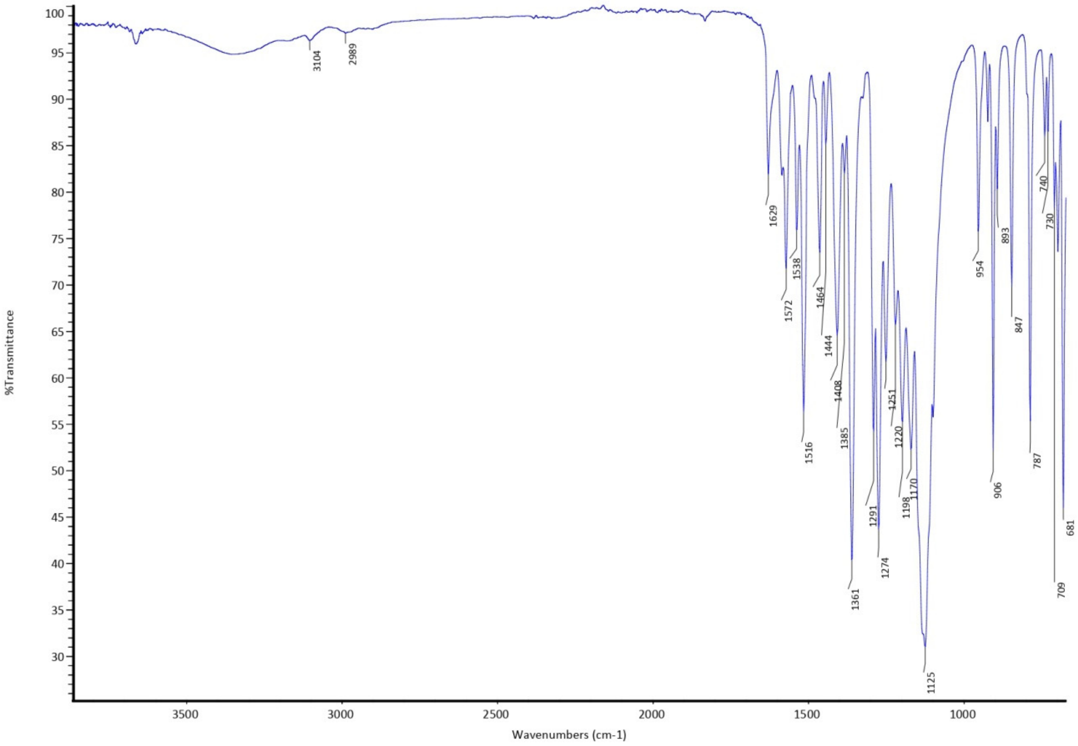

Figure S27. FT-IR spectrum of $[Zn(L^{CF3})_2]$ (**6**).

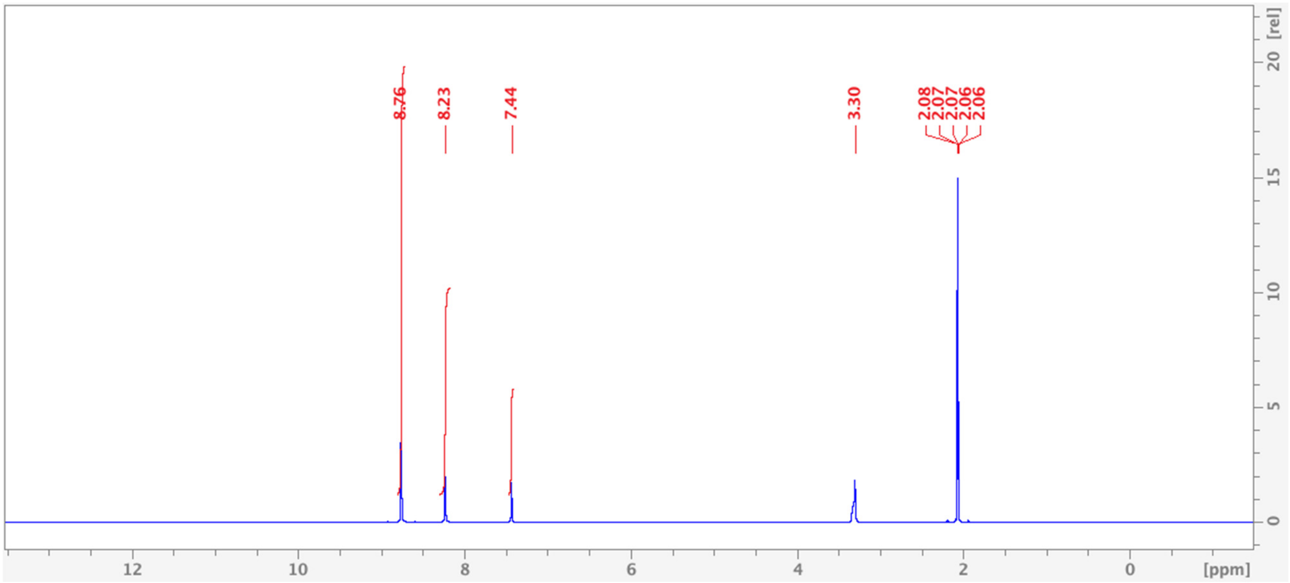

Figure S28. ^{1}H-NMR spectrum of $[Zn(L^{CF3})_2]$ (**6**) in Acetone-d_6.

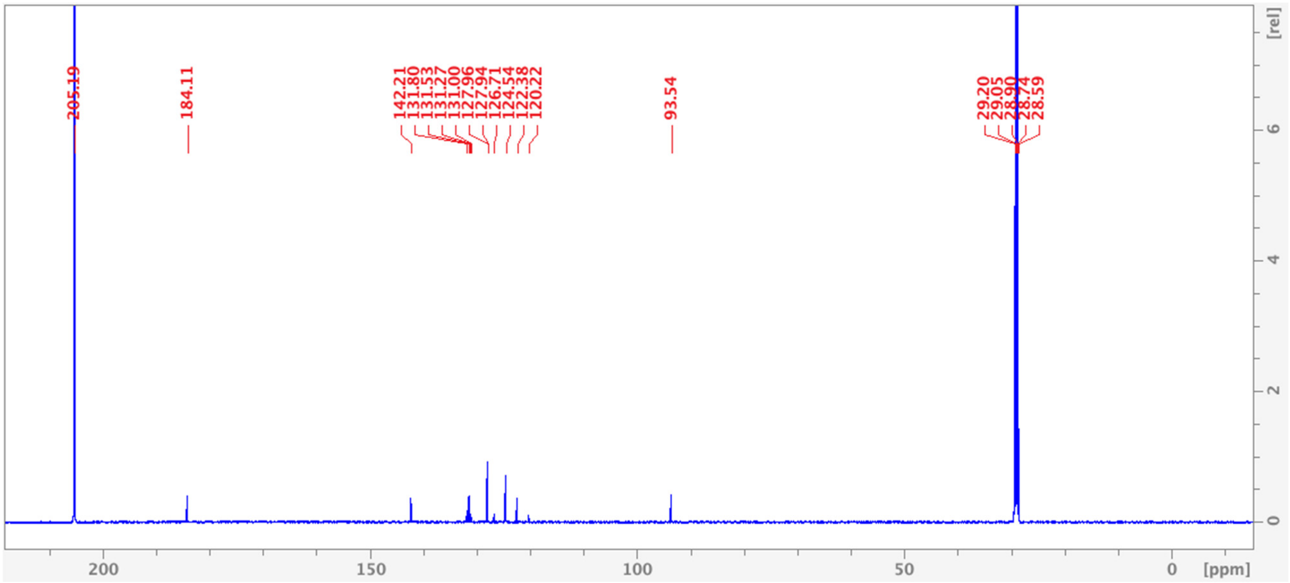

Figure S29. ^{13}C{^{1}H}-NMR spectrum of $[Zn(L^{CF3})_2]$ (**6**) in Acetone-d_6.

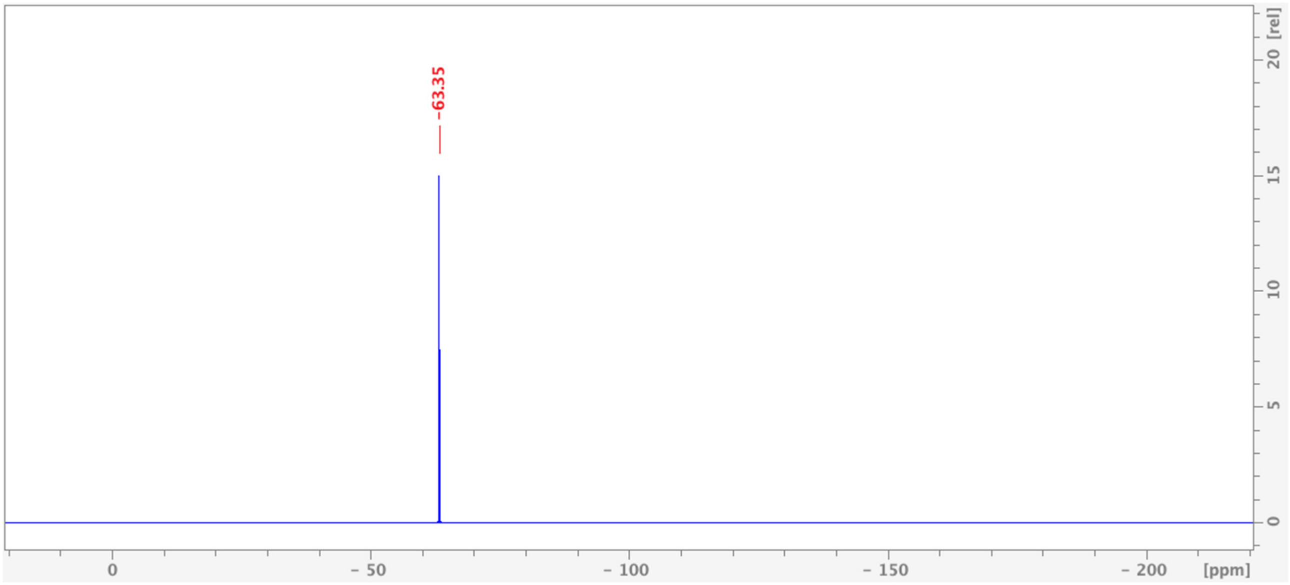

Figure S30. $^{19}F\{^1H\}$-NMR spectrum of $[Zn(L^{CF3})_2]$ (**6**) in Acetone-d_6.

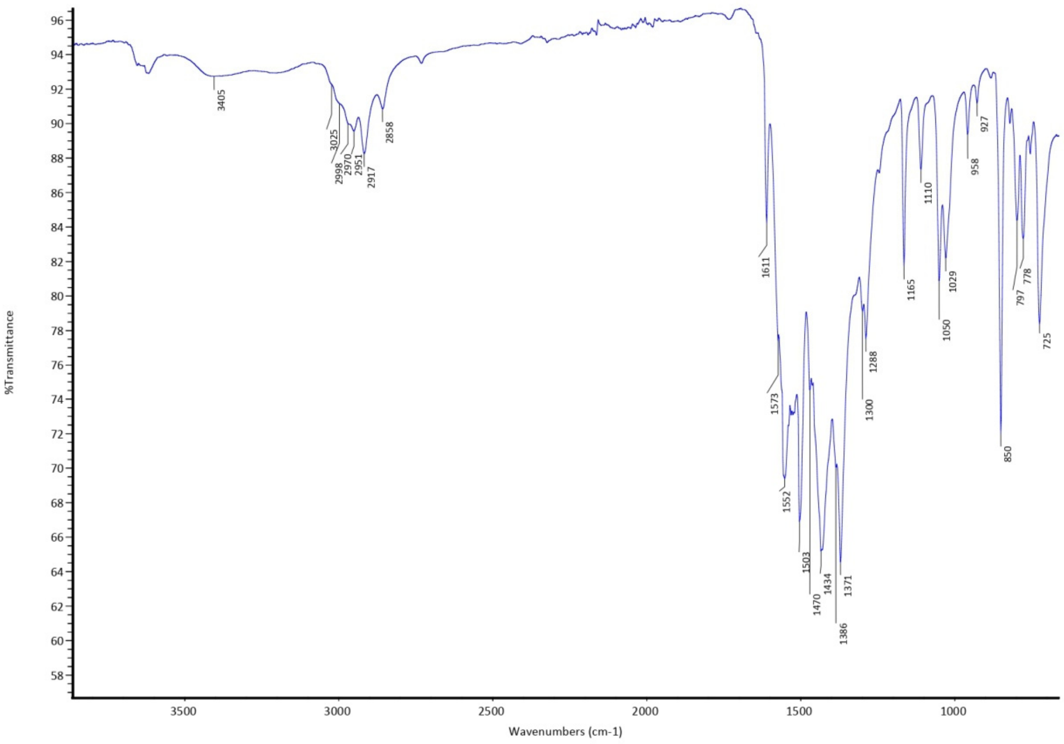

Figure S31. FT-IR spectrum of $[Mn(L^{Mes})_2(H_2O)_2]$ (**7**).

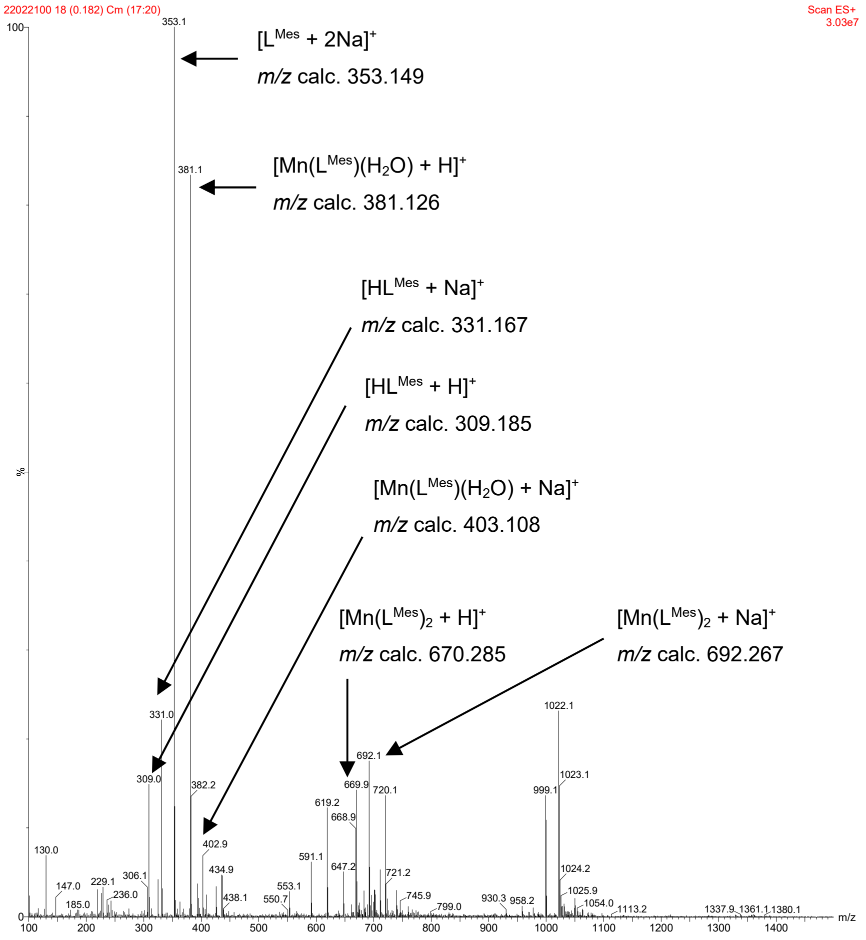

Figure S32. ESI-MS(+) spectrum of [Mn(L^{Mes})$_2$(H_2O)$_2$] (**7**) in CH_3OH.

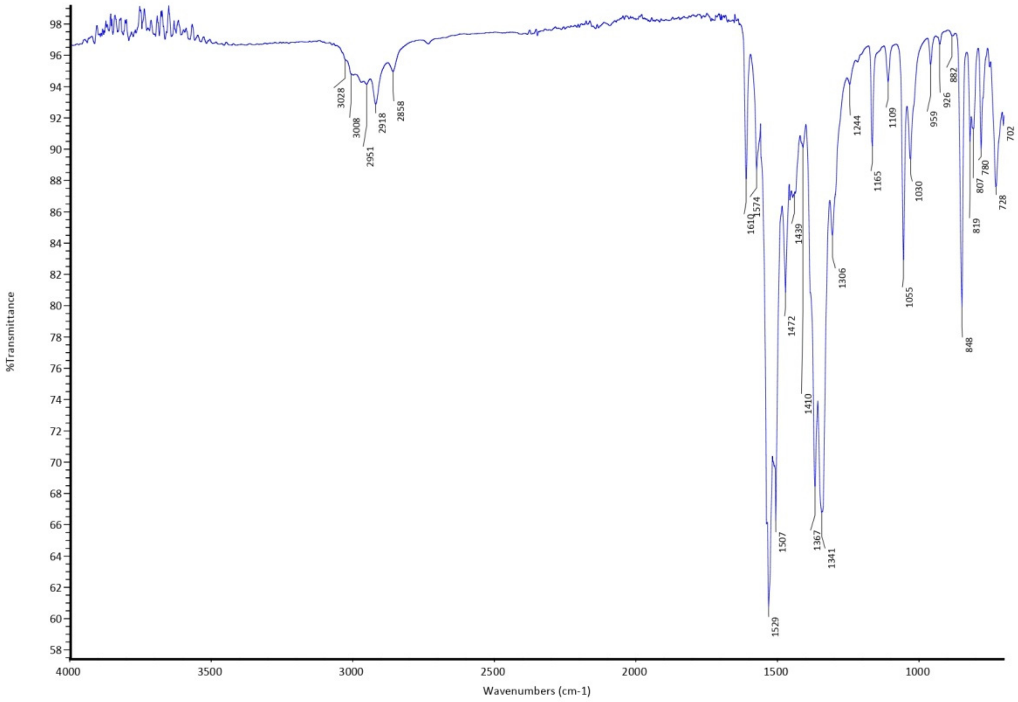

Figure S33. FT-IR spectrum of [Fe(L^{Mes})$_2$] (**8**).

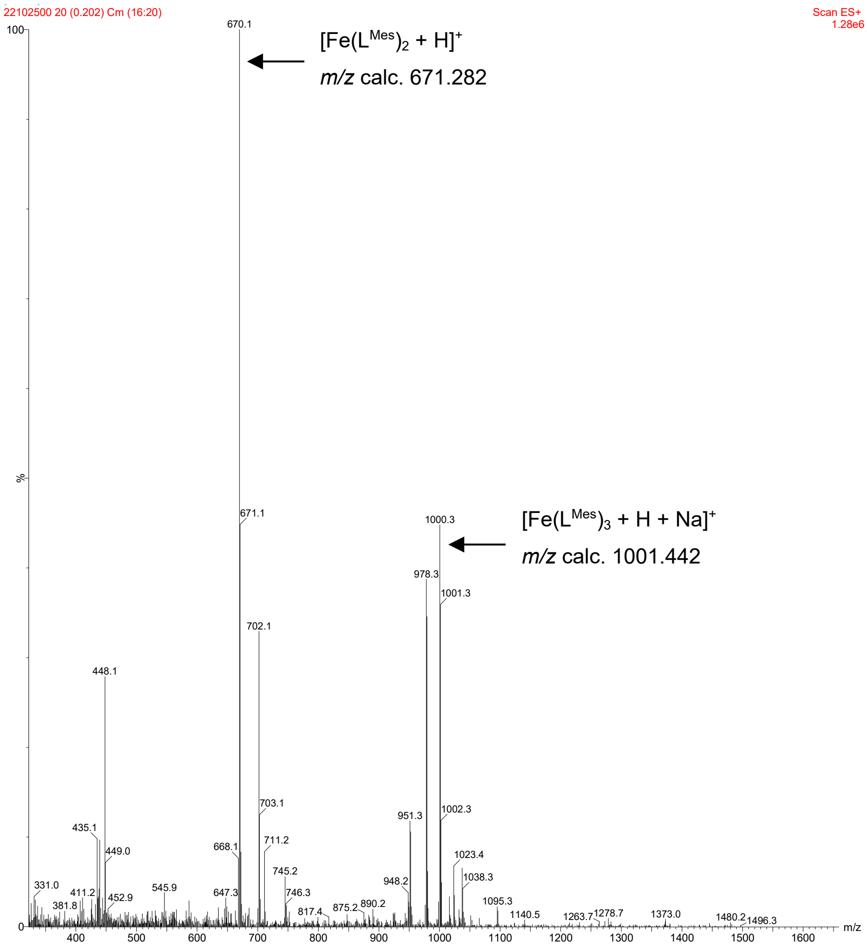

Figure S34. ESI-MS(+) spectrum of $[Fe(L^{Mes})_2]$ (**8**) in CH_3CN.

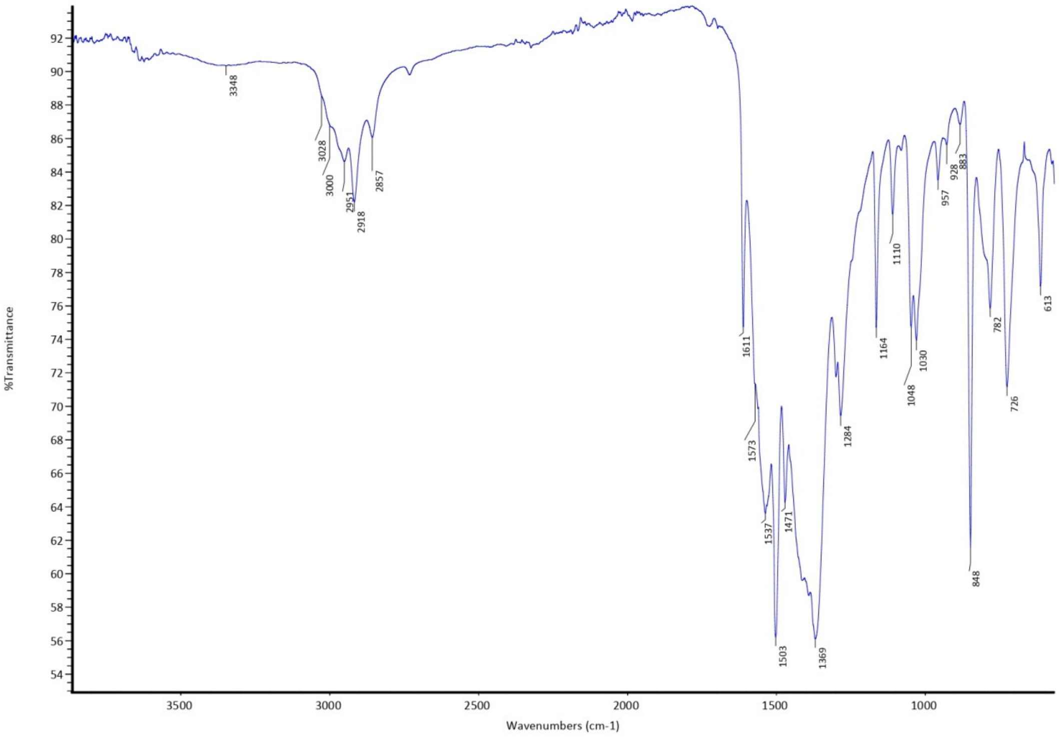

Figure S35. FT-IR spectrum of $[Co(L^{Mes})_2(H_2O)_2]$ (**9**).

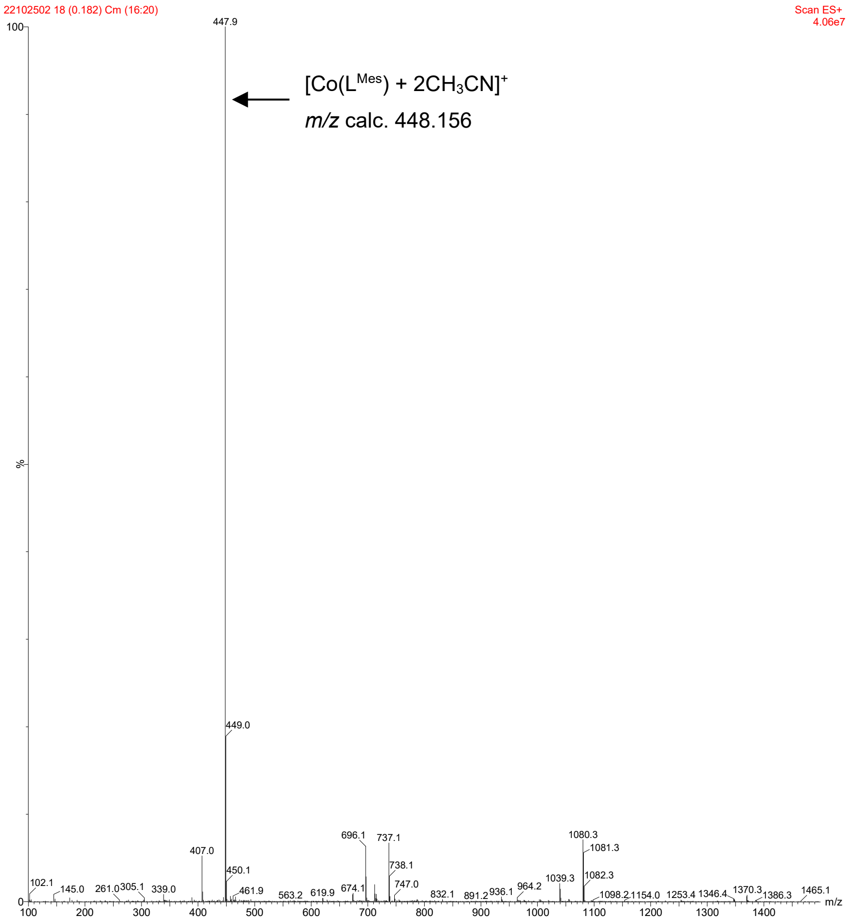

Figure S36. ESI-MS(+) spectrum of $[Co(L^{Mes})_2(H_2O)_2]$ (**9**) in CH_3OH/CH_3CN.

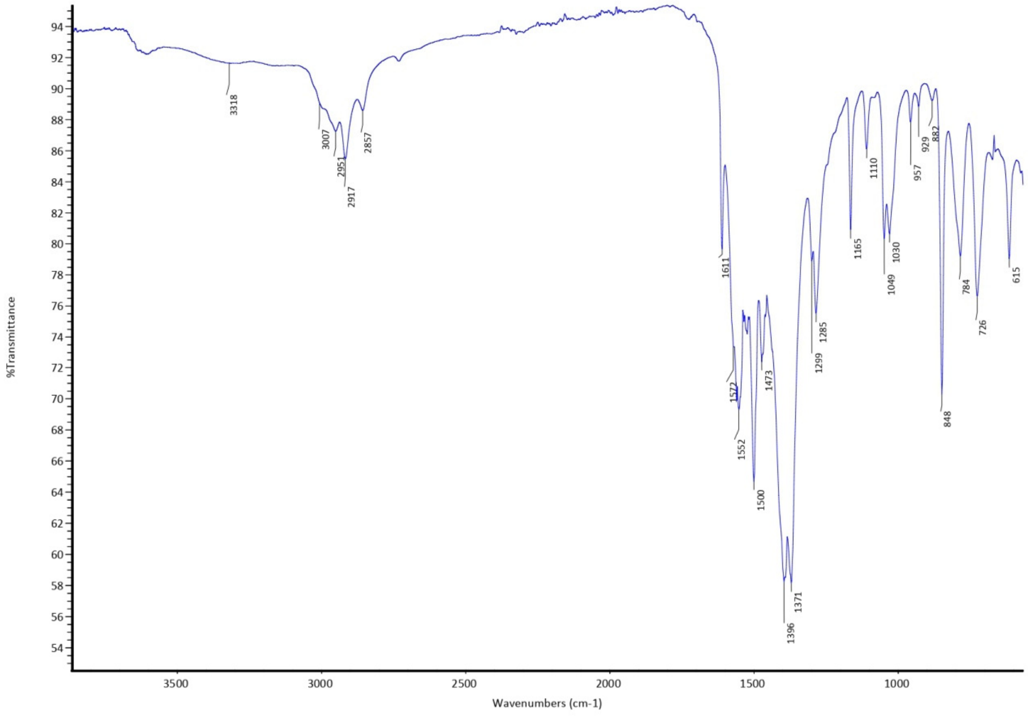

Figure S37. FT-IR spectrum of $[Ni(L^{Mes})_2(H_2O)_2]$ (**10**).

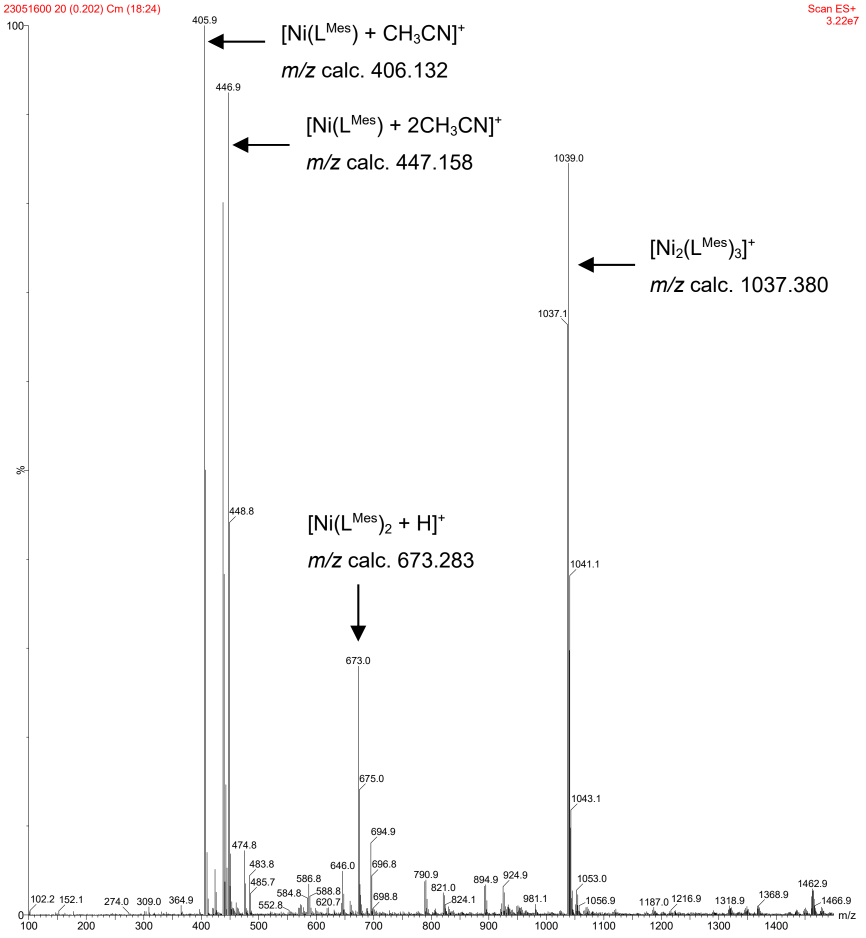

Figure S38. ESI-MS(+) spectrum of [Ni(L^{Mes})$_2$(H$_2$O)$_2$] (**10**) in CH$_3$CN.

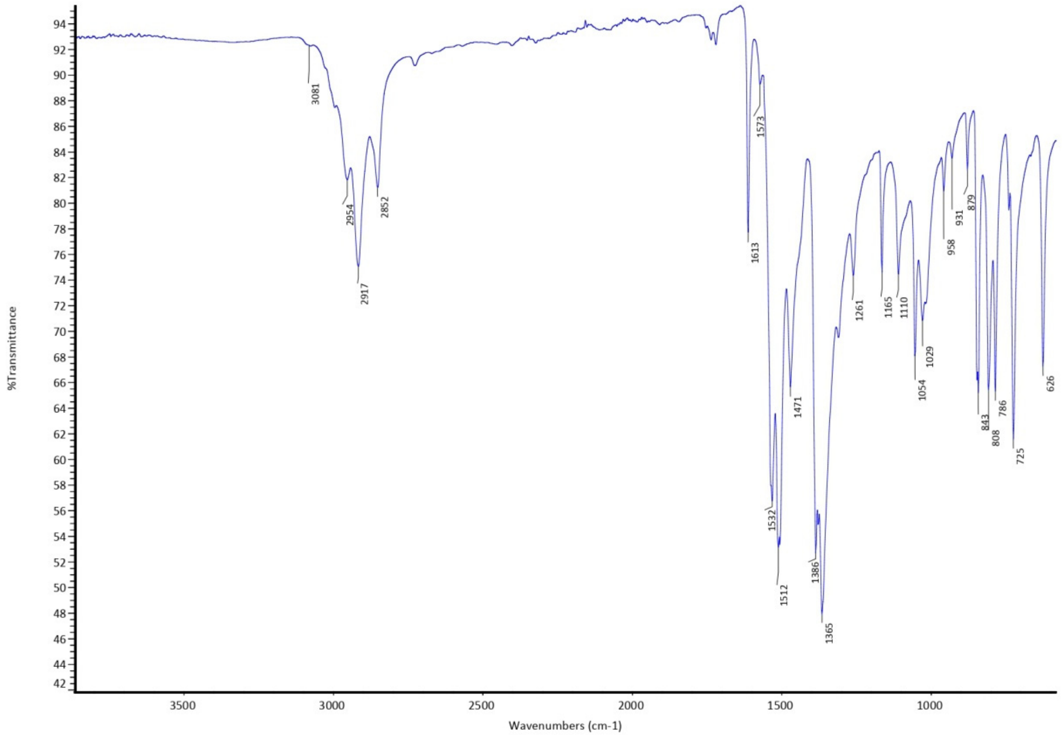

Figure S39. FT-IR spectrum of [Cu(L^{Mes})$_2$] (**11**).

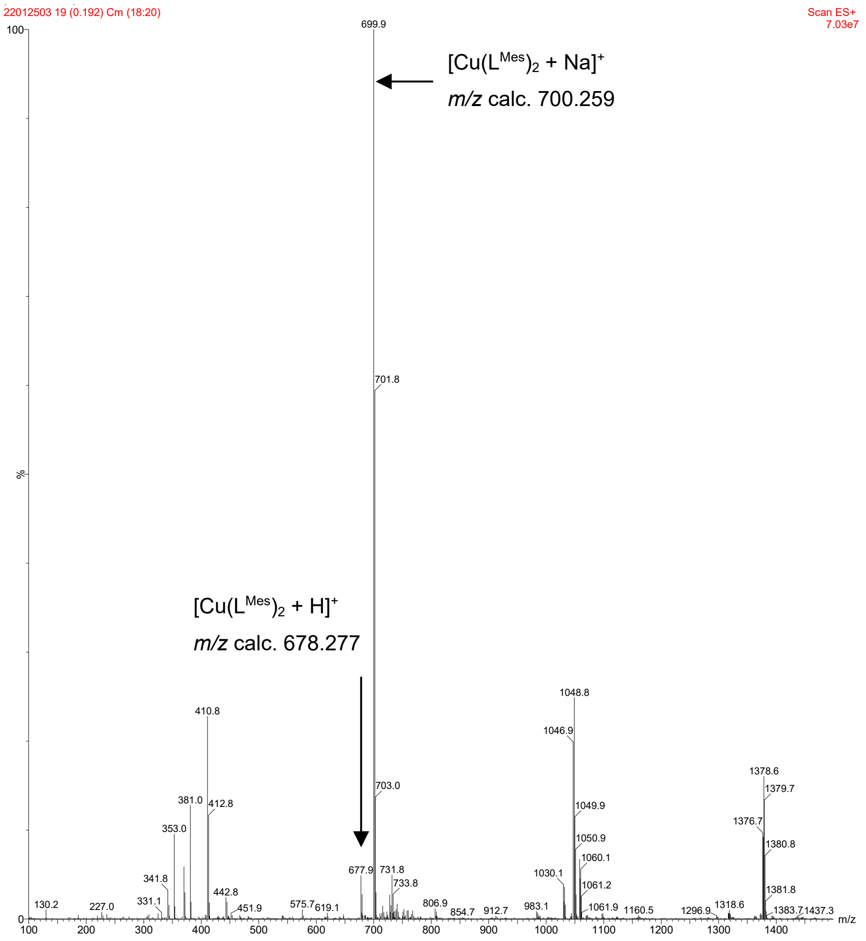

Figure S40. ESI-MS(+) spectrum of [Cu(L^{Mes})$_2$] (**11**) in CH_3OH.

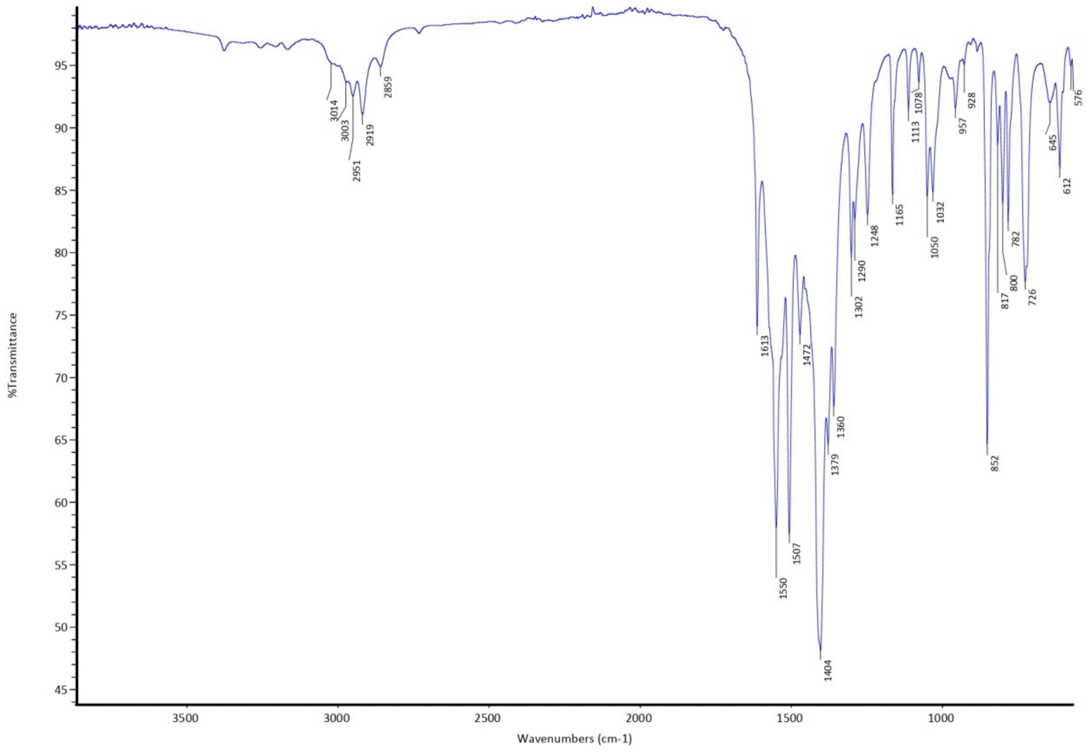

Figure S41. FT-IR spectrum of [Zn(L^{Mes})$_2$] (**12**).

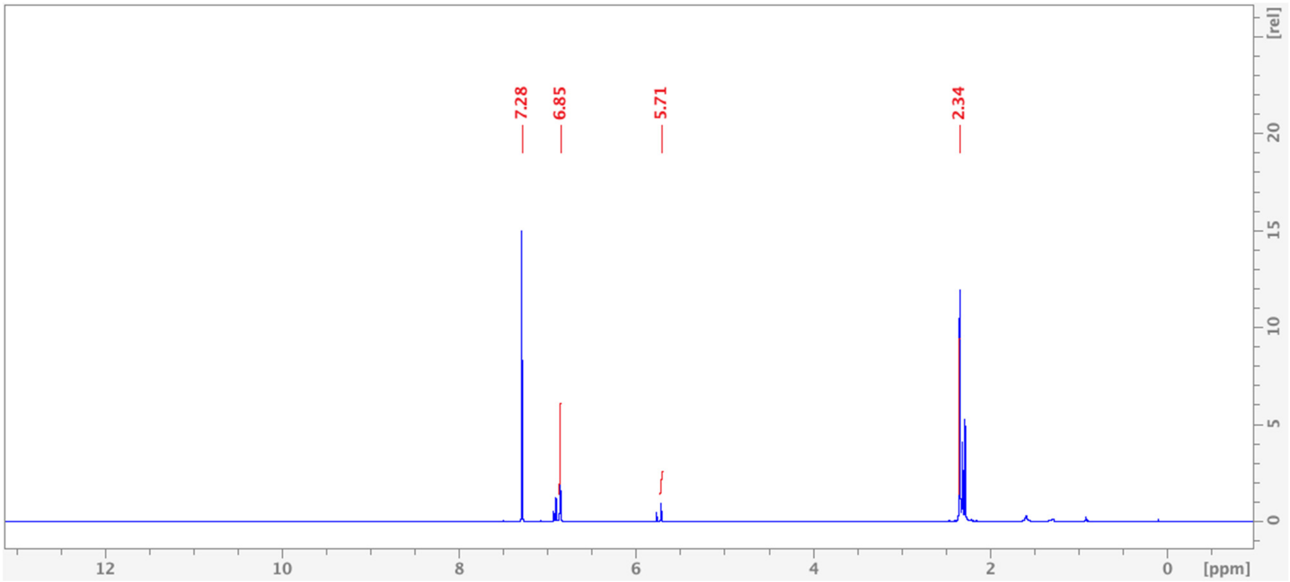

Figure S42. ^{1}H-NMR spectrum of $[Zn(L^{Mes})_2]$ (**12**) in $CDCl_3$.

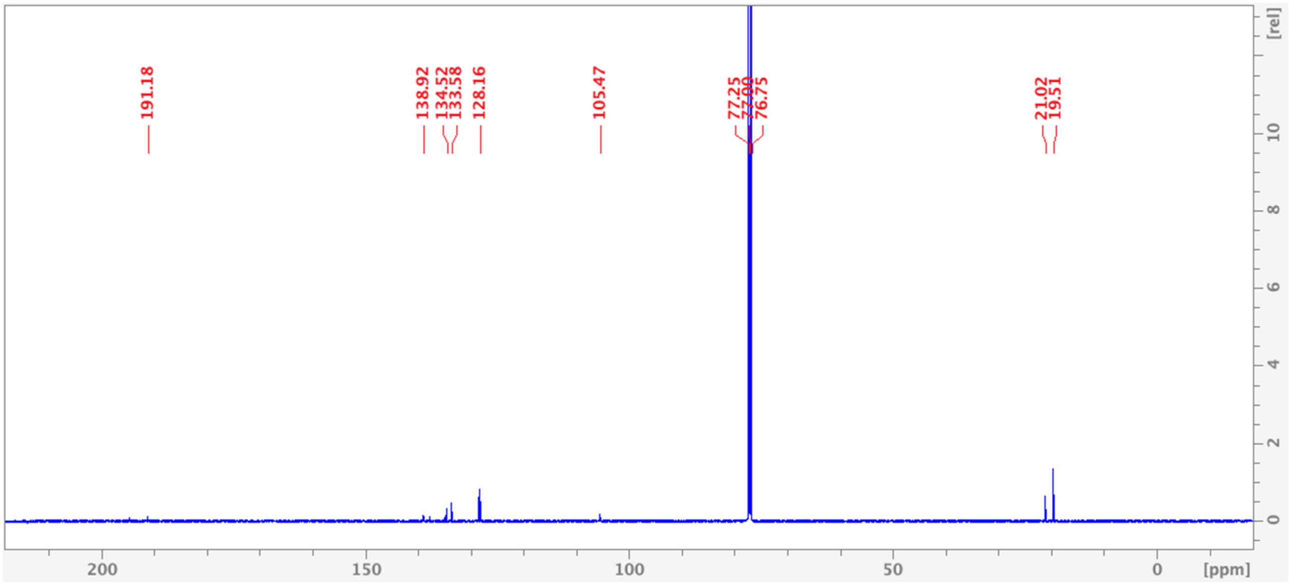

Figure S43. $^{13}C\{^{1}H\}$-NMR spectrum of $[Zn(L^{Mes})_2]$ (**12**) in in $CDCl_3$.

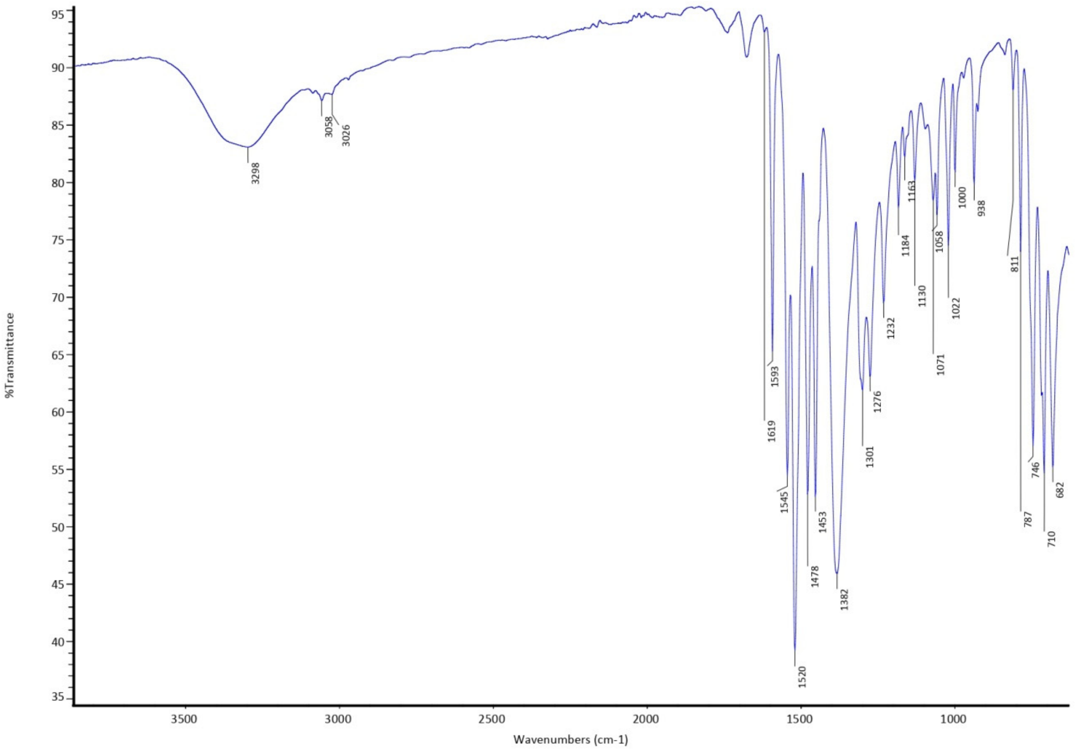

Figure S44. FT-IR spectrum of $[Mn(L^{Ph})_2(H_2O)_2]$ (**13**).

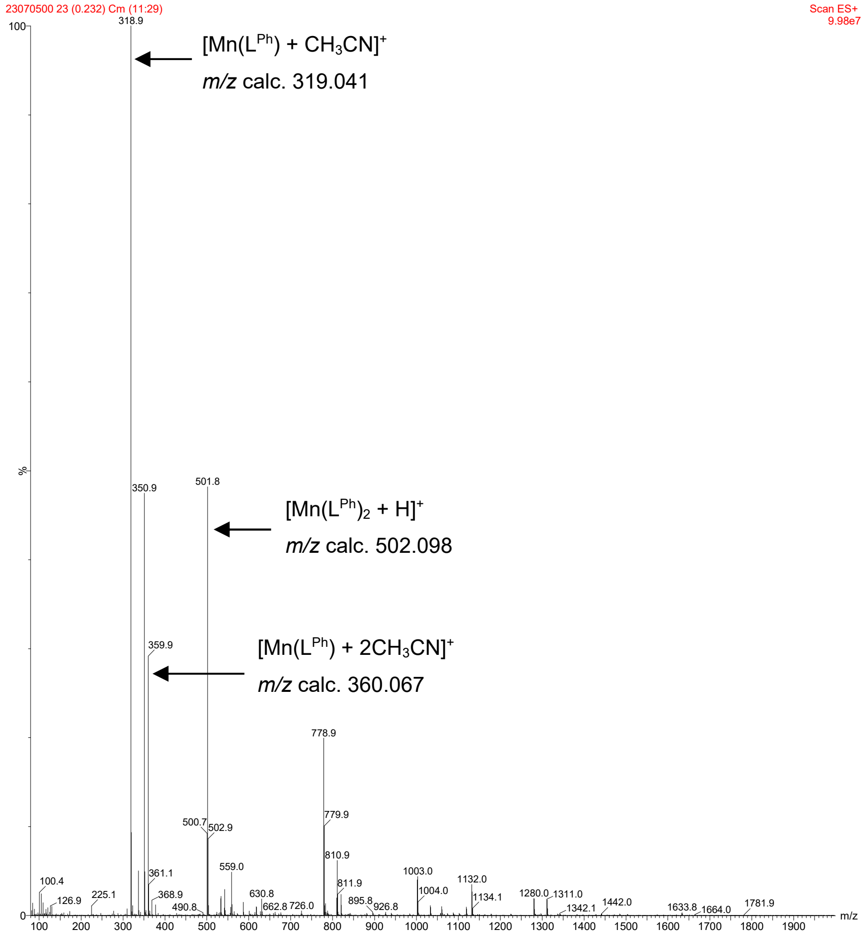

Figure S45. ESI-MS(+) spectrum of $[Mn(L^{Ph})_2(H_2O)_2]$ (**13**) in CH_3OH.

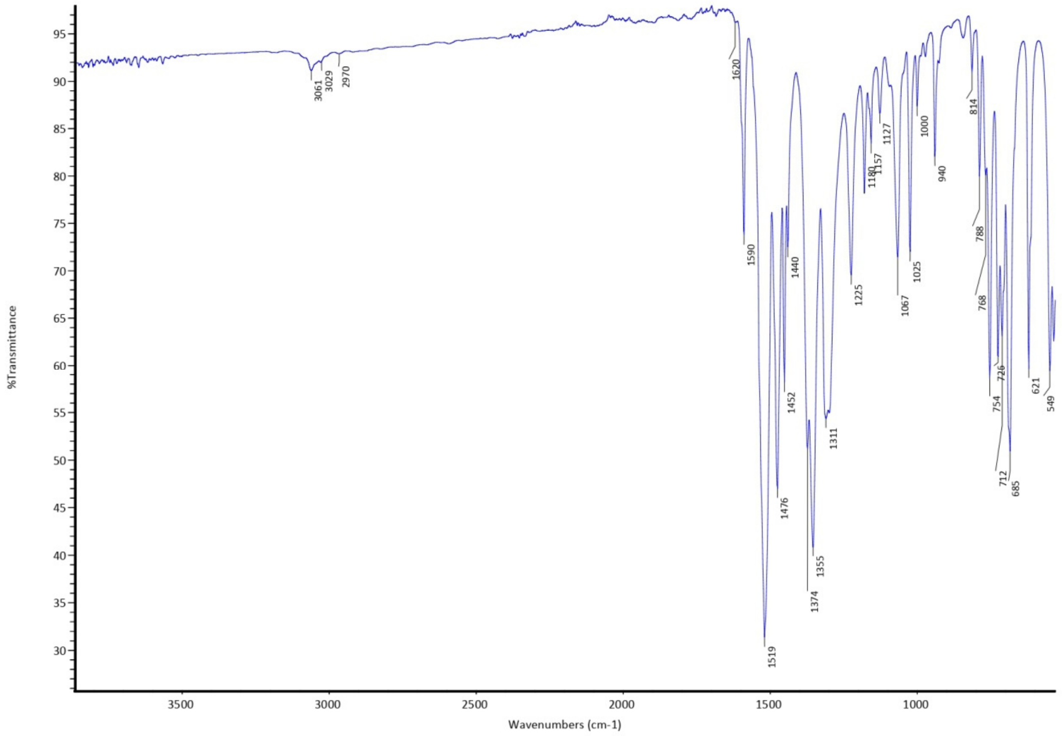

Figure S46. FT-IR spectrum of $[Fe(L^{Ph})_2]$ (**14**).

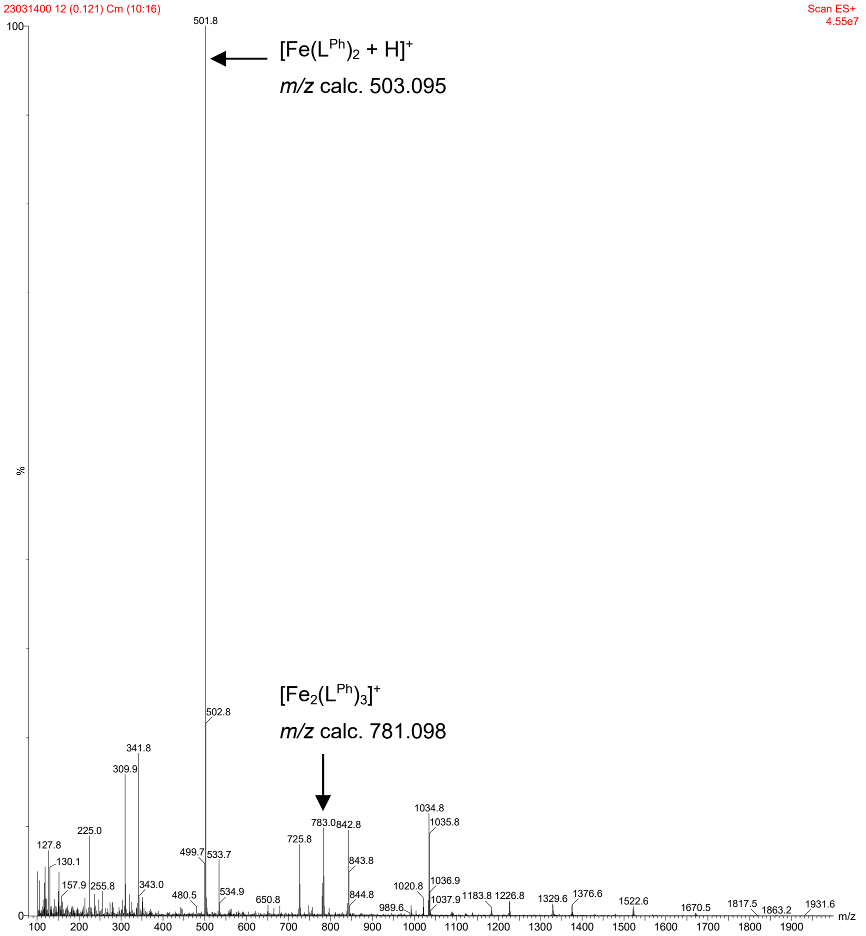

Figure S47. ESI-MS(+) spectrum of $[Fe(L^{Ph})_2]$ (**14**) in CH_3OH/CH_3CN.

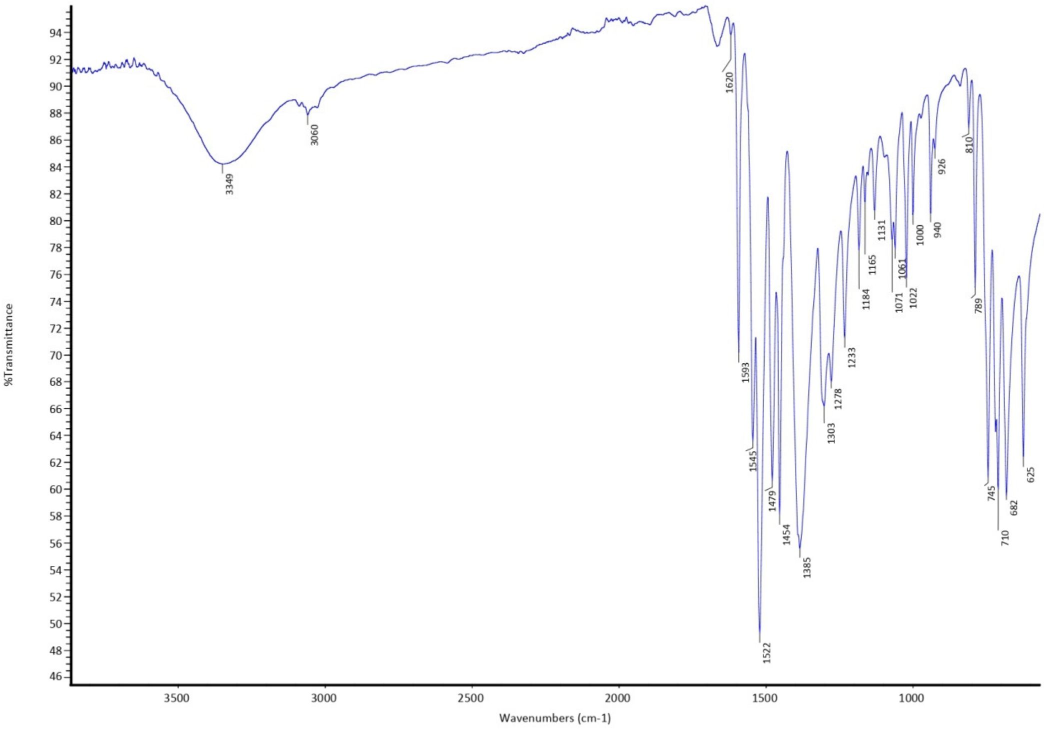

Figure S48. FT-IR spectrum of $[Co(L^{Ph})_2(H_2O)_2]$ (**15**).

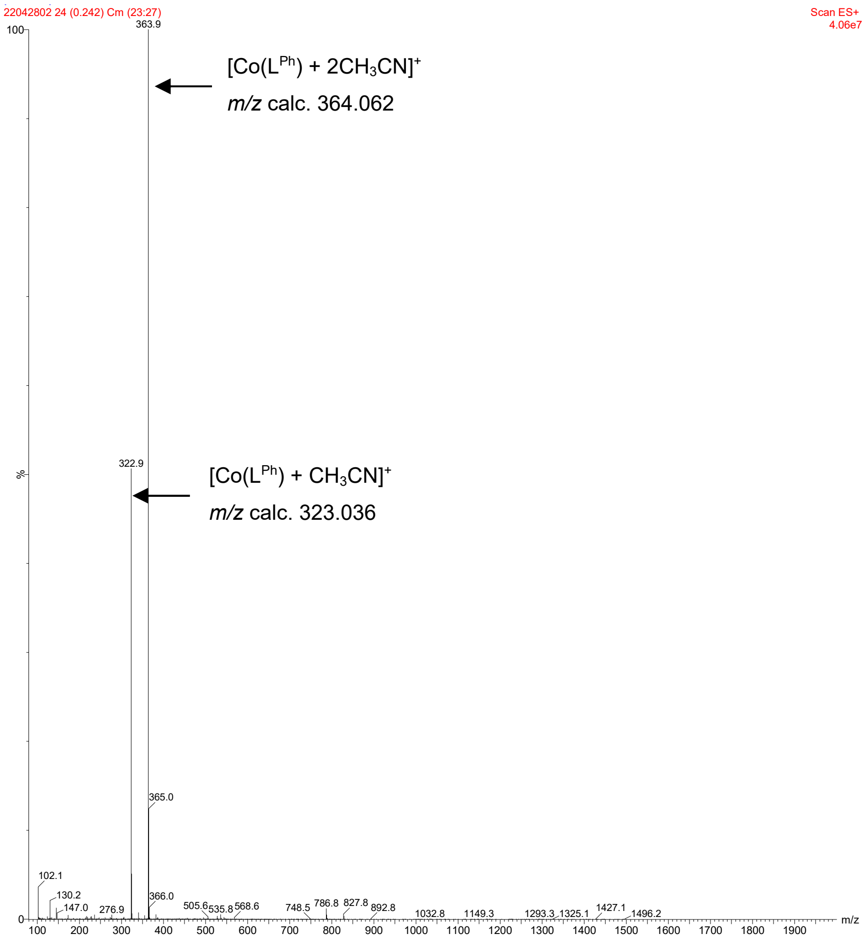

Figure S49. ESI-MS(+) spectrum of $[Co(L^{Ph})_2(H_2O)_2]$ (**15**) in CH_3CN.

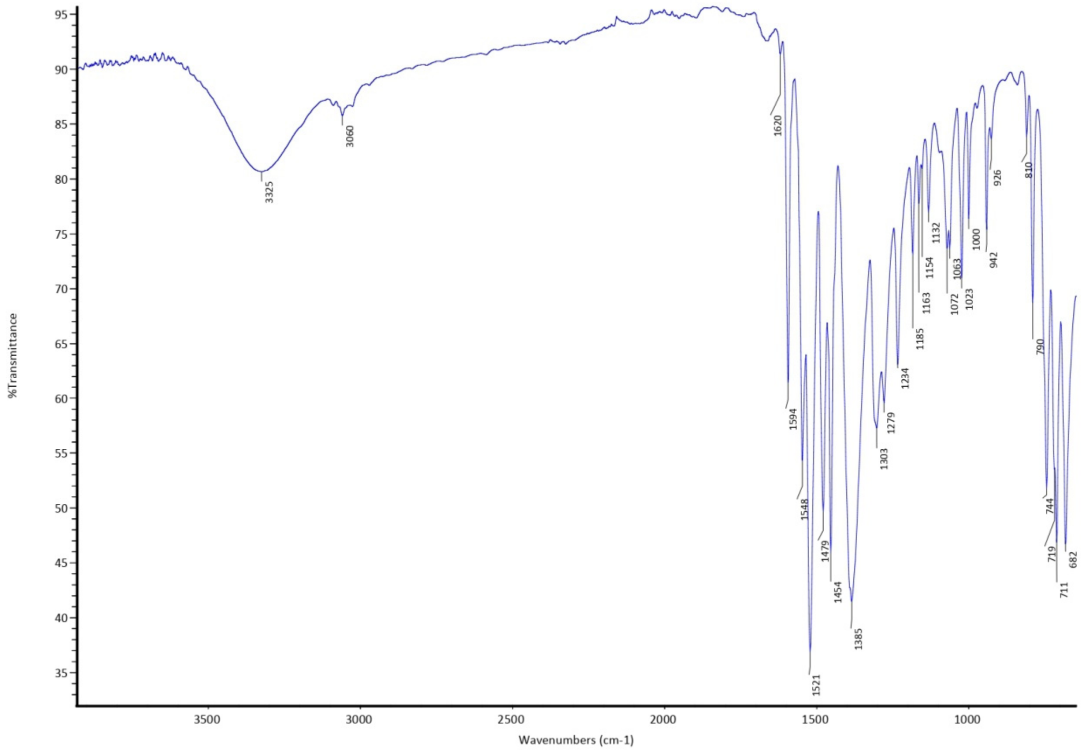

Figure S50. FT-IR spectrum of [Ni(L^{Ph})$_2$(H_2O)$_2$] (**16**).

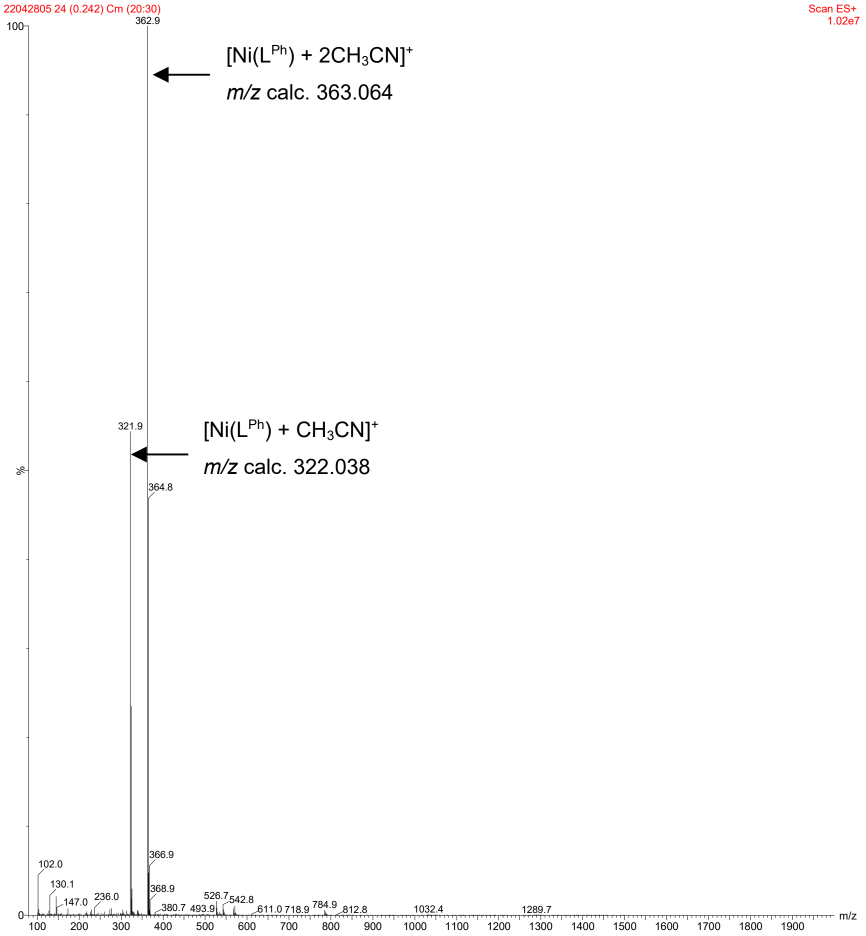

Figure S51. ESI-MS(+) spectrum of [Ni(L^{Ph})$_2$(H_2O)$_2$] (**16**) in CH_3CN.

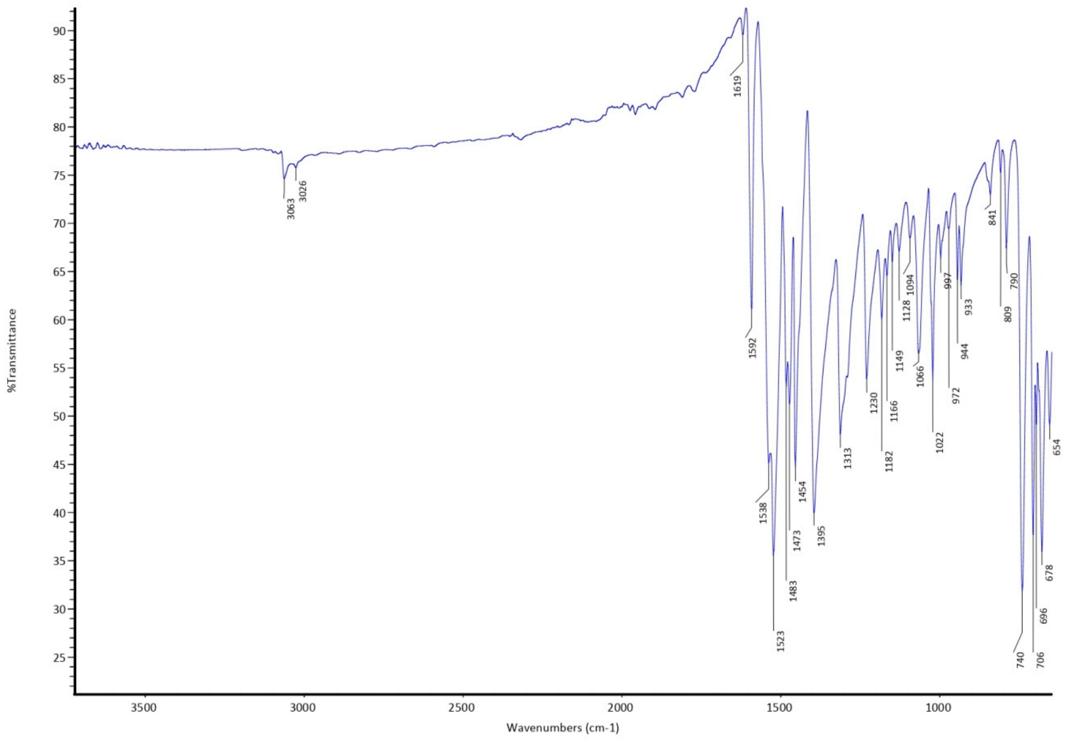

Figure S52. FT-IR spectrum of [Cu(L^{Ph})$_2$] (**17**).

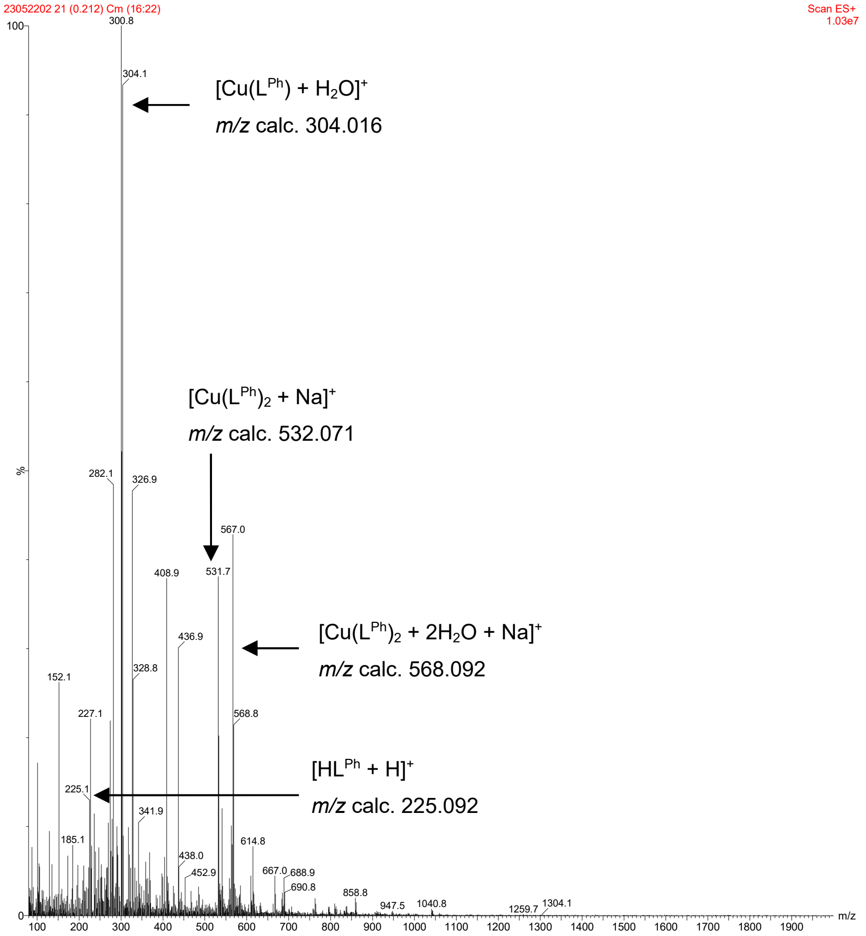

Figure S53 ESI-MS(+) spectrum of [Cu(L^{Ph})$_2$] (**17**) in CH_3OH.

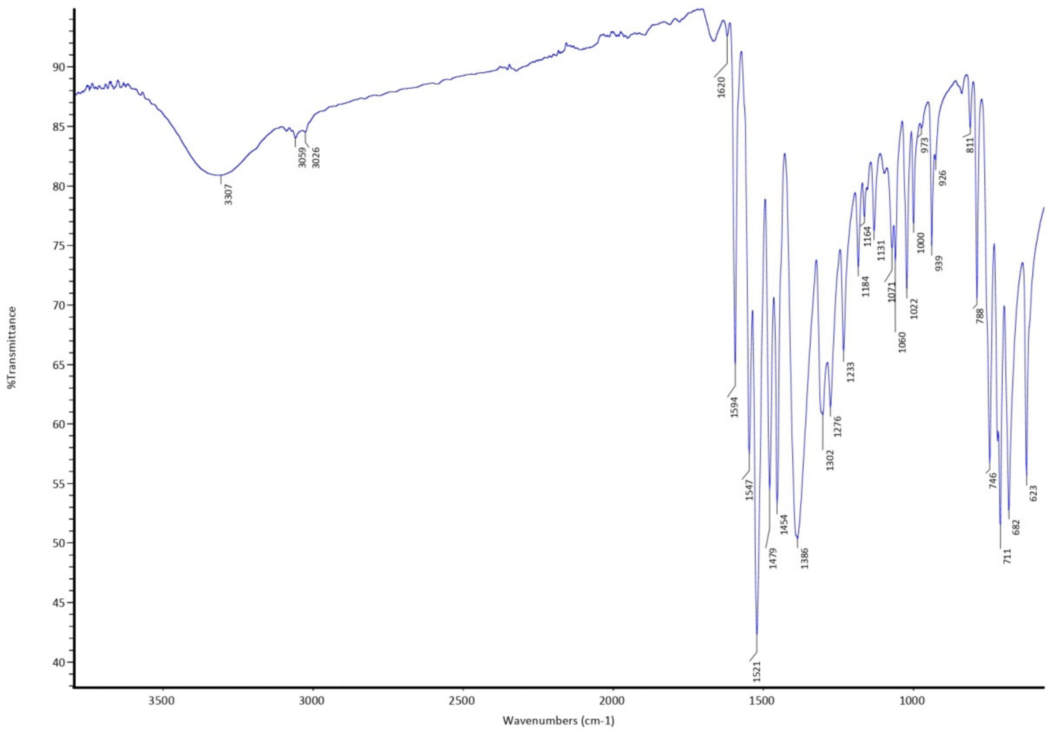

Figure S54. FT-IR spectrum of [Zn(L^{Ph})$_2$]·2H$_2$O (**18**).

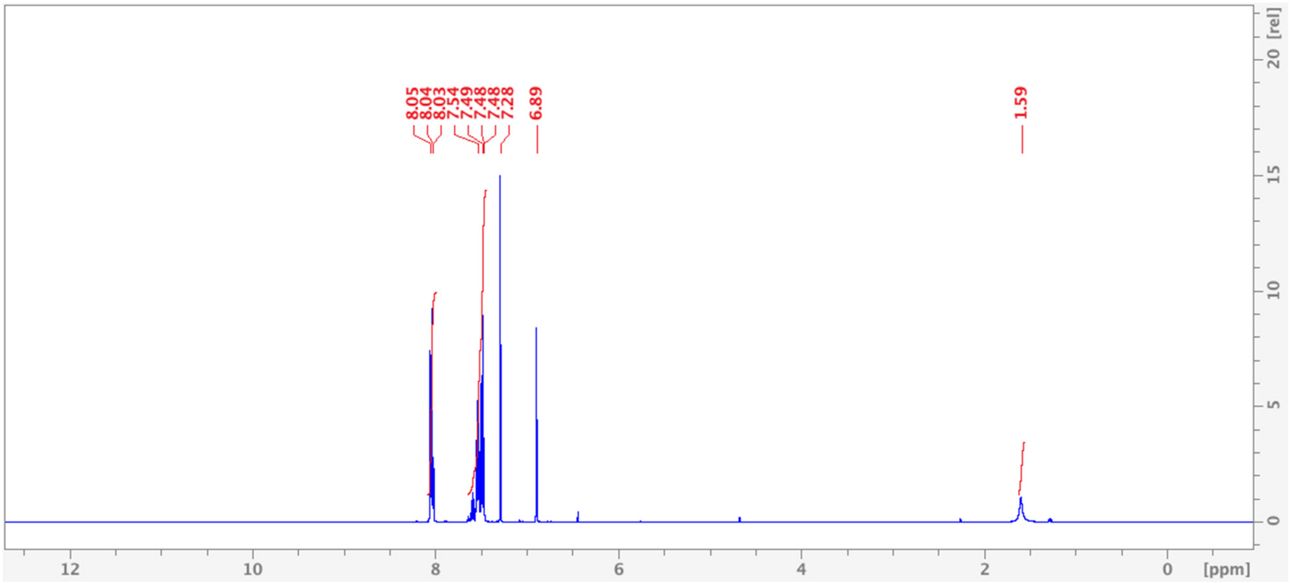

Figure S55. ^{1}H-NMR spectrum of [Zn(L^{Ph})$_2$]·2H$_2$O (**18**) in in CDCl$_3$.

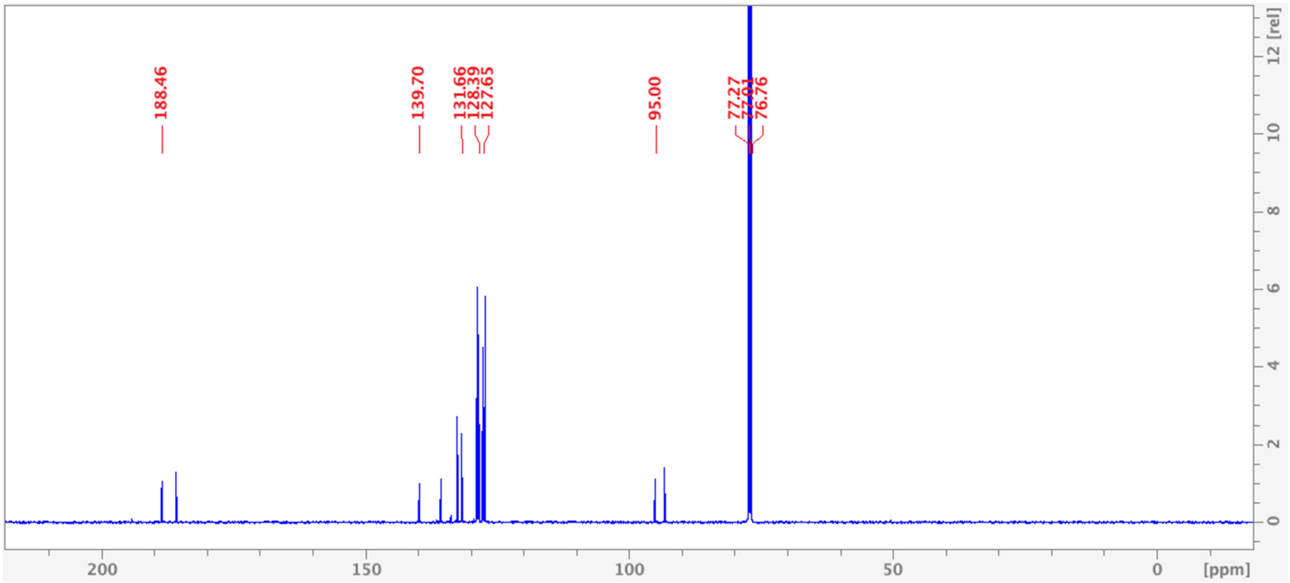

Figure S56. $^{13}C\{^{1}H\}$-NMR spectrum of $[Zn(L^{Ph})_2]\cdot 2H_2O$ (**18**) in $CDCl_3$.

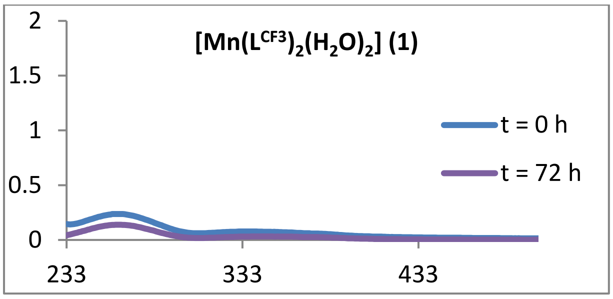
[Mn(L^CF3)2(H2O)2] (1)
t = 0 h
t = 72 h
2
1.5
1
0.5
0
233
333
433

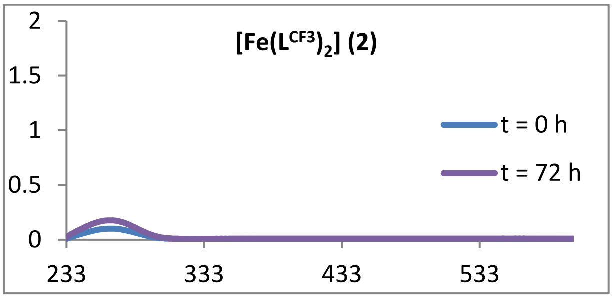
[Fe(L^CF3)2] (2)
t = 0 h
t = 72 h
2
1.5
1
0.5
0
233
333
433
533

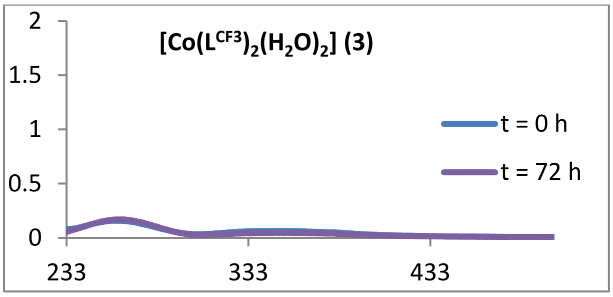
[Co(L^CF3)2(H2O)2] (3)
t = 0 h
t = 72 h
2
1.5
1
0.5
0
233
333
433

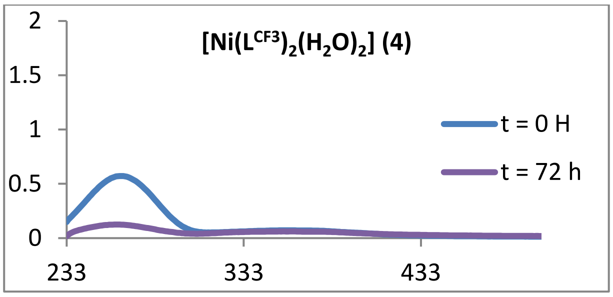
[Ni(L^CF3)2(H2O)2] (4)
t = 0 H
t = 72 h
2
1.5
1
0.5
0
233
333
433

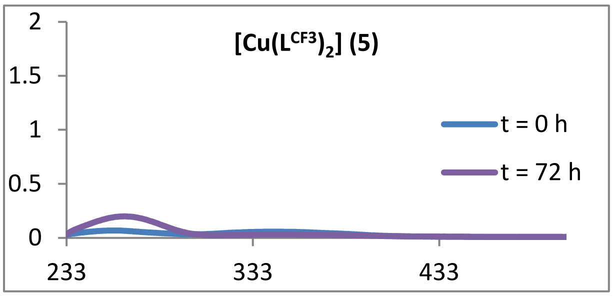
[Cu(L^CF3)2] (5)
t = 0 h
t = 72 h
2
1.5
1
0.5
0
233
333
433

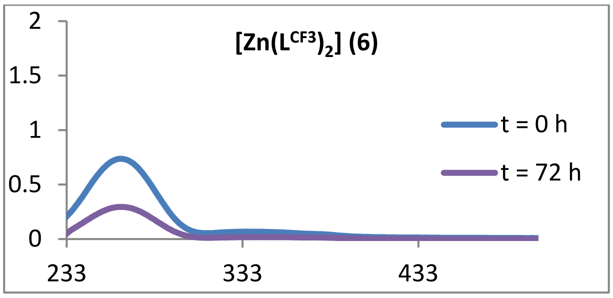
[Zn(L^CF3)2] (6)
t = 0 h
t = 72 h
2
1.5
1
0.5
0
233
333
433

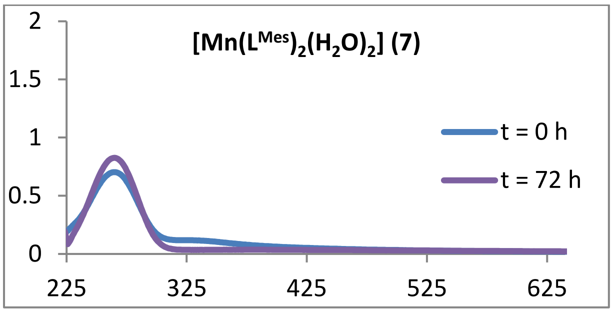
[Mn(L^Mes)2(H2O)2] (7)
t = 0 h
t = 72 h
2
1.5
1
0.5
0
225
325
425
525
625

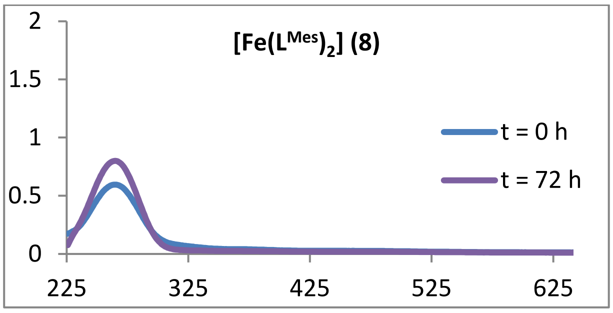
[Fe(L^Mes)2] (8)
t = 0 h
t = 72 h
2
1.5
1
0.5
0
225
325
425
525
625

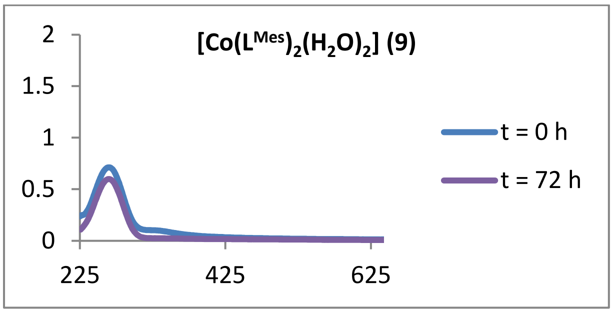
[Co(L^Mes)2(H2O)2] (9)
t = 0 h
t = 72 h
2
1.5
1
0.5
0
225
425
625

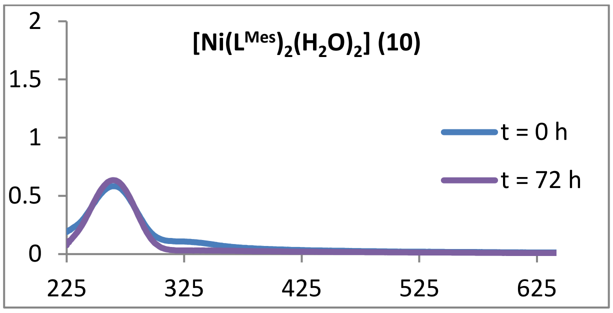
[Ni(L^Mes)2(H2O)2] (10)
t = 0 h
t = 72 h
2
1.5
1
0.5
0
225
325
425
525
625

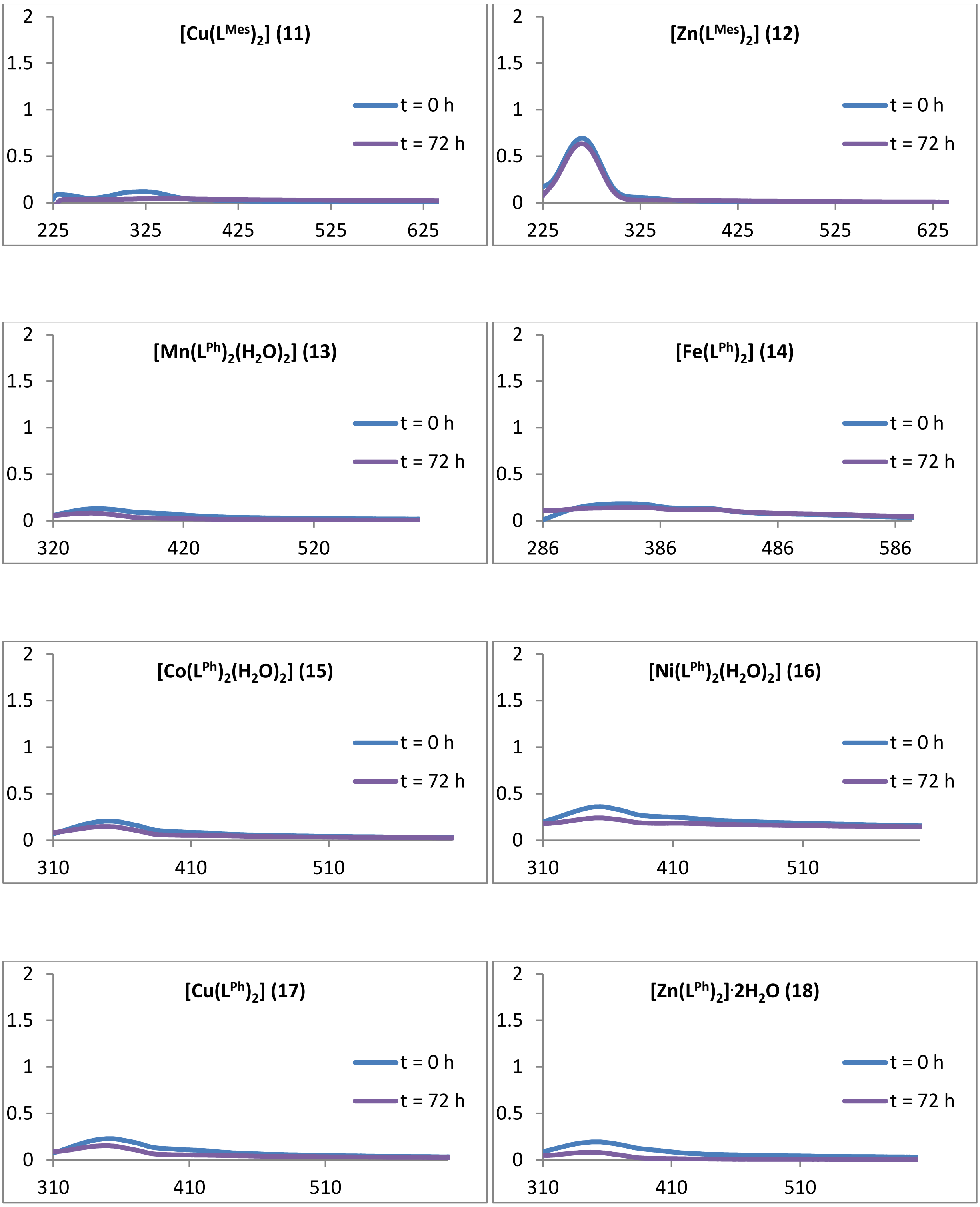

Figure S57. Stability studies. All complexes were dissolved at 50 μM in 0.5% DMSO/ physiological solution. UV-Visible spectra were recorded at t = 0 h and t = 72 h.

**Table S1**. Crystal data and structure refinement for $[Cu(L^{Mes})_2]$ (**11**).	
Identification code	dia39_0m_a
Empirical formula	$C_{42}H_{46}CuO_4$
Formula weight	678.33
Temperature/K	100.03
Crystal system	monoclinic
Space group	C2/c
a/Å	21.2012(19)
b/Å	10.8405(10)
c/Å	16.7071(15)
α/°	90
β/°	112.3000(10)
γ/°	90
Volume/Å^3	3552.6(6)
Z	4
ρ_{calc}g/cm^3	1.268
μ/mm^{-1}	0.655
F(000)	1436.0
Crystal size/mm^3	0.34 × 0.11 × 0.05
Radiation	Mo Kα (λ = 0.71073)
2Θ range for data collection/°	4.152 to 62.122
Index ranges	-30 ≤ h ≤ 29, -14 ≤ k ≤ 14, -23 ≤ l ≤ 23
Reflections collected	20246
Independent reflections	5227 [R_{int} = 0.0284, R_{sigma} = 0.0275]
Data/restraints/parameters	5227/0/220
Goodness-of-fit on F^2	1.048
Final R indexes [I>=2σ (I)]	R_1 = 0.0331, wR_2 = 0.0872
Final R indexes [all data]	R_1 = 0.0416, wR_2 = 0.0915
Largest diff. peak/hole / e Å^{-3}	0.52/-0.30

Table S2. Bond Lengths for $[Cu(L^{Mes})_2]$ (**11**).

**Atom**	**Atom**	**Length/Å**		**Atom**	**Atom**	**Length/Å**
Cu	O1^1	1.9166(9)		C6	C7	1.390(2)
Cu	O1	1.9166(9)		C7	C8	1.389(2)
Cu	O2^1	1.9070(9)		C7	C11	1.5080(19)
Cu	O2	1.9071(9)		C8	C9	1.3973(18)
O1	C1	1.2763(15)		C9	C12	1.5069(19)
O2	C3	1.2768(15)		C13	C14	1.4032(18)
C1	C2	1.3973(18)		C13	C18	1.3968(18)
C1	C4	1.5024(17)		C14	C15	1.3898(18)
C2	C3	1.3967(17)		C14	C19	1.5077(18)
C3	C13	1.4995(17)		C15	C16	1.3953(19)
C4	C5	1.4012(19)		C16	C17	1.3892(19)
C4	C9	1.3980(19)		C16	C20	1.5079(17)
C5	C6	1.3936(18)		C17	C18	1.3955(18)
C5	C10	1.5050(19)		C18	C21	1.5061(19)

^{1}1/2-X,3/2-Y,-Z

**Table S3**. Bond Angles for [Cu(L^{Mes})$_2$] (**11**).								
**Atom**	**Atom**	**Atom**	**Angle/˚**		**Atom**	**Atom**	**Atom**	**Angle/˚**
O1	Cu	O1[1]	180.0		C6	C7	C11	120.58(14)
O2[1]	Cu	O1	86.33(4)		C8	C7	C6	118.28(12)
O2	Cu	O1	93.67(4)		C8	C7	C11	121.13(14)
O2[1]	Cu	O1[1]	93.67(4)		C7	C8	C9	121.93(13)
O2	Cu	O1[1]	86.33(4)		C4	C9	C12	121.79(12)
O2[1]	Cu	O2	180.0		C8	C9	C4	118.47(12)
C1	O1	Cu	125.70(8)		C8	C9	C12	119.75(13)
C3	O2	Cu	125.98(8)		C14	C13	C3	119.03(11)
O1	C1	C2	125.33(12)		C18	C13	C3	120.07(11)
O1	C1	C4	115.61(11)		C18	C13	C14	120.88(11)
C2	C1	C4	119.05(11)		C13	C14	C19	120.81(12)
C3	C2	C1	123.88(12)		C15	C14	C13	118.76(12)
O2	C3	C2	125.28(12)		C15	C14	C19	120.43(12)
O2	C3	C13	116.75(11)		C14	C15	C16	121.61(12)
C2	C3	C13	117.97(11)		C15	C16	C20	120.29(12)
C5	C4	C1	118.48(12)		C17	C16	C15	118.24(12)
C9	C4	C1	120.67(12)		C17	C16	C20	121.47(12)
C9	C4	C5	120.85(11)		C16	C17	C18	121.99(12)
C4	C5	C10	121.12(12)		C13	C18	C21	120.97(12)
C6	C5	C4	118.65(12)		C17	C18	C13	118.42(12)
C6	C5	C10	120.22(13)		C17	C18	C21	120.61(12)
C7	C6	C5	121.80(13)					

[1]1/2-X,3/2-Y,-Z

**Table S4**. Crystal data and structure refinement for $[Zn(L^{Mes})_2]$ (**12**).	
Identification code	dia100_0m_a
Empirical formula	$C_{42}H_{46}O_4Zn$
Formula weight	680.16
Temperature/K	99.98
Crystal system	triclinic
Space group	P-1
a/Å	11.7040(14)
b/Å	12.0704(14)
c/Å	13.6623(16)
α/°	104.205(2)
β/°	99.304(2)
γ/°	101.855(2)
Volume/Å^3	1784.8(4)
Z	2
ρ_{calc}g/cm^3	1.266
μ/mm^{-1}	0.729
F(000)	720.0
Crystal size/mm^3	0.35 × 0.342 × 0.057
Radiation	Mo Kα (λ = 0.71073)
2Θ range for data collection/°	3.154 to 61.966
Index ranges	-16 ≤ h ≤ 16, -16 ≤ k ≤ 16, -19 ≤ l ≤ 19
Reflections collected	18950
Independent reflections	10255 [R_{int} = 0.0235, R_{sigma} = 0.0393]
Data/restraints/parameters	10255/0/436
Goodness-of-fit on F^2	1.037
Final R indexes [I>=2σ (I)]	R_1 = 0.0364, wR_2 = 0.0964
Final R indexes [all data]	R_1 = 0.0454, wR_2 = 0.1006
Largest diff. peak/hole / e Å^{-3}	0.48/-0.53

Table S5. Bond Lengths for [Zn(L^{Mes})$_2$] (**12**).

Atom	Atom	Length/Å		Atom	Atom	Length/Å
Zn	O1	1.9426(11)		C16	C17	1.393(2)
Zn	O2	1.9282(11)		C16	C20	1.508(2)
Zn	O3	1.9384(10)		C17	C18	1.395(2)
Zn	O4	1.9435(11)		C18	C21	1.510(2)
O1	C1	1.2764(17)		C22	C23	1.402(2)
O2	C3	1.2826(17)		C22	C25	1.4991(19)
O3	C22	1.2835(16)		C23	C24	1.401(2)
O4	C24	1.2807(17)		C24	C34	1.4994(19)
C1	C2	1.404(2)		C25	C26	1.407(2)
C1	C4	1.4952(19)		C25	C30	1.407(2)
C2	C3	1.397(2)		C26	C27	1.392(2)
C3	C13	1.4998(19)		C26	C31	1.510(2)
C4	C5	1.403(2)		C27	C28	1.392(2)
C4	C9	1.403(2)		C28	C29	1.388(2)
C5	C6	1.392(2)		C28	C32	1.508(2)
C5	C10	1.509(2)		C29	C30	1.399(2)
C6	C7	1.389(2)		C30	C33	1.515(2)
C7	C8	1.387(3)		C34	C35	1.401(2)
C7	C11	1.508(2)		C34	C39	1.402(2)
C8	C9	1.393(2)		C35	C36	1.396(2)
C9	C12	1.508(2)		C35	C40	1.509(2)
C13	C14	1.403(2)		C36	C37	1.393(2)
C13	C18	1.403(2)		C37	C38	1.387(2)
C14	C15	1.389(2)		C37	C41	1.506(2)
C14	C19	1.512(2)		C38	C39	1.397(2)
C15	C16	1.393(2)		C39	C42	1.503(2)

**Table S6**. Bond Angles for [Zn(L^{Mes})$_2$] (**12**).								
**Atom**	**Atom**	**Atom**	**Angle/˚**		**Atom**	**Atom**	**Atom**	**Angle/˚**
O1	Zn	O4	111.67(5)		C17	C16	C20	121.06(14)
O2	Zn	O1	95.78(4)		C16	C17	C18	121.51(14)
O2	Zn	O3	123.15(5)		C13	C18	C21	121.84(13)
O2	Zn	O4	117.02(5)		C17	C18	C13	118.76(14)
O3	Zn	O1	111.70(5)		C17	C18	C21	119.40(14)
O3	Zn	O4	98.06(4)		O3	C22	C23	125.50(13)
C1	O1	Zn	123.74(10)		O3	C22	C25	116.10(12)
C3	O2	Zn	123.03(10)		C23	C22	C25	118.39(12)
C22	O3	Zn	121.54(9)		C24	C23	C22	126.90(13)
C24	O4	Zn	120.57(9)		O4	C24	C23	126.52(13)
O1	C1	C2	125.06(13)		O4	C24	C34	116.91(12)
O1	C1	C4	114.77(13)		C23	C24	C34	116.53(12)
C2	C1	C4	120.17(12)		C26	C25	C22	119.12(13)
C3	C2	C1	125.68(13)		C26	C25	C30	119.94(13)
O2	C3	C2	126.28(13)		C30	C25	C22	120.94(13)
O2	C3	C13	114.54(12)		C25	C26	C31	122.19(13)
C2	C3	C13	119.16(12)		C27	C26	C25	118.89(13)
C5	C4	C1	118.14(13)		C27	C26	C31	118.92(13)
C9	C4	C1	121.07(13)		C28	C27	C26	122.19(13)
C9	C4	C5	120.72(14)		C27	C28	C32	120.62(14)
C4	C5	C10	121.97(14)		C29	C28	C27	118.07(13)
C6	C5	C4	118.92(14)		C29	C28	C32	121.30(14)
C6	C5	C10	119.10(15)		C28	C29	C30	121.86(14)
C7	C6	C5	121.48(15)		C25	C30	C33	123.18(13)
C6	C7	C11	120.48(17)		C29	C30	C25	118.98(13)
C8	C7	C6	118.45(15)		C29	C30	C33	117.82(13)
C8	C7	C11	121.05(17)		C35	C34	C24	118.33(13)
C7	C8	C9	122.28(15)		C35	C34	C39	121.03(13)
C4	C9	C12	122.64(14)		C39	C34	C24	120.59(13)
C8	C9	C4	118.14(15)		C34	C35	C40	121.12(14)
C8	C9	C12	119.20(15)		C36	C35	C34	118.54(14)
C14	C13	C3	118.37(13)		C36	C35	C40	120.34(14)
C18	C13	C3	120.93(13)		C37	C36	C35	121.61(15)
C18	C13	C14	120.66(13)		C36	C37	C41	120.76(16)
C13	C14	C19	121.12(13)		C38	C37	C36	118.53(14)
C15	C14	C13	118.70(14)		C38	C37	C41	120.70(15)
C15	C14	C19	120.16(14)		C37	C38	C39	121.90(14)
C14	C15	C16	121.90(14)		C34	C39	C42	121.33(14)
C15	C16	C17	118.43(14)		C38	C39	C34	118.31(14)
C15	C16	C20	120.50(14)		C38	C39	C42	120.33(14)

**Table S7**. Crystal data and structure refinement for $[Cu(L^{CF3})_2(THF)]\cdot 2(THF)$.	
Identification code	dia95_0m_a
Empirical formula	$C_{50}H_{38}CuF_{24}O_7$
Formula weight	1270.34
Temperature/K	200.07
Crystal system	triclinic
Space group	P-1
a/Å	12.980(4)
b/Å	13.172(4)
c/Å	17.416(5)
α/°	69.511(4)
β/°	75.588(4)
γ/°	75.482(5)
Volume/Å^3	2657.3(13)
Z	2
ρ_{calc}g/cm^3	1.588
μ/mm^{-1}	0.545
F(000)	1278.0
Crystal size/mm^3	0.305 × 0.145 × 0.139
Radiation	Mo Kα (λ = 0.71073)
2Θ range for data collection/°	2.536 to 58.298
Index ranges	-17 ≤ h ≤ 17, -18 ≤ k ≤ 17, -23 ≤ l ≤ 23
Reflections collected	29871
Independent reflections	14217 [R_{int} = 0.0192, R_{sigma} = 0.0318]
Data/restraints/parameters	14217/714/1077
Goodness-of-fit on F^2	1.045
Final R indexes [I>=2σ (I)]	R_1 = 0.0488, wR_2 = 0.1371
Final R indexes [all data]	R_1 = 0.0779, wR_2 = 0.1548
Largest diff. peak/hole / e Å^{-3}	0.43/-0.27

Table S8. Bond Lengths for [Cu(L^{CF3})$_2$(THF)]·2(THF).

Atom	Atom	Length/Å		Atom	Atom	Length/Å
Cu1	O1	1.9239(14)		C29	F13	1.305(7)
Cu1	O2	1.9221(14)		C29	F14	1.311(7)
Cu1	O3	1.9207(13)		C29	F15	1.293(6)
Cu1	O4	1.9200(13)		C29	F13A	1.285(11)
Cu1	O5	2.323(2)		C29	F14A	1.318(8)
F4	C11	1.328(3)		C29	F15A	1.280(8)
F5	C11	1.336(3)		C30	F16	1.526(6)
F6	C11	1.318(3)		C30	F17	1.259(6)
F7	C18	1.319(3)		C30	F18	1.242(6)
F8	C18	1.332(3)		C30	F16A	1.205(8)
F9	C18	1.320(3)		C30	F17A	1.249(7)
O1	C1	1.264(2)		C30	F18A	1.627(7)
O2	C3	1.272(2)		C31	C32	1.394(2)
O3	C20	1.265(2)		C31	C36	1.388(3)
O4	C22	1.272(2)		C32	C33	1.389(3)
O5	C51	1.456(9)		C33	C34	1.381(3)
O5	C54	1.466(8)		C33	C37	1.484(3)
O5	C39	1.375(8)		C34	C35	1.385(3)
O5	C42	1.377(9)		C35	C36	1.389(3)
C1	C2	1.403(3)		C35	C38	1.499(3)
C1	C4	1.501(3)		C37	F19	1.219(5)
C2	C3	1.401(3)		C37	F20	1.494(5)
C3	C12	1.502(3)		C37	F21	1.283(4)
C4	C5	1.394(3)		C37	F19A	1.577(7)
C4	C9	1.397(3)		C37	F20A	1.221(7)
C5	C6	1.388(3)		C37	F21A	1.255(8)
C6	C7	1.389(3)		C38	F22	1.324(5)
C6	C10	1.496(4)		C38	F23	1.296(5)
C7	C8	1.377(3)		C38	F24	1.258(6)
C8	C9	1.388(3)		C38	F22A	1.286(6)
C8	C11	1.498(3)		C38	F23A	1.259(8)
C10	F1	1.302(6)		C38	F24A	1.324(7)
C10	F2	1.238(6)		C51	C52	1.542(11)
C10	F3	1.417(5)		C52	C53	1.459(9)
C10	F1A	1.423(6)		C53	C54	1.523(11)
C10	F2A	1.285(6)		O6	C1I	1.456(13)
C10	F3A	1.179(6)		O6	C1L	1.425(14)
C12	C13	1.400(3)		C1I	C1J	1.519(11)
C12	C17	1.388(3)		C1J	C1K	1.505(12)
C13	C14	1.391(3)		C1K	C1L	1.519(12)
C14	C15	1.378(3)		O7	C1M	1.419(8)
C14	C18	1.509(3)		O7	C1P	1.440(11)
C15	C16	1.390(3)		C1M	C1N	1.496(10)
C16	C17	1.391(3)		C1N	C1O	1.554(10)
C16	C19	1.500(3)		C1O	C1P	1.523(11)
C19	F10	1.257(5)		O8	C1A	1.455(13)
C19	F11	1.323(5)		O8	C1D	1.423(13)
C19	F12	1.413(4)		O9	C1E	1.437(12)
C19	F10A	1.303(5)		O9	C1H	1.442(11)
C19	F11A	1.416(5)		C1A	C1B	1.569(13)
C19	F12A	1.194(5)		C1B	C1C	1.545(14)
C20	C21	1.398(3)		C1C	C1D	1.555(13)
C20	C23	1.507(2)		C1E	C1F	1.519(11)
C21	C22	1.392(3)		C1F	C1G	1.548(11)
C22	C31	1.503(2)		C1G	C1H	1.482(11)

C23	C24	1.386(3)		C39	C40	1.528(11)
C23	C28	1.391(3)		C40	C41	1.411(9)
C24	C25	1.392(3)		C41	C42	1.552(12)
C25	C26	1.382(3)		O10	C1Q	1.425(14)
C25	C29	1.502(3)		O10	C1T	1.407(14)
C26	C27	1.376(3)		C1Q	C1R	1.565(14)
C27	C28	1.391(3)		C1R	C1S	1.541(14)
C27	C30	1.496(3)		C1S	C1T	1.571(14)

Table S9. Bond Angles for [Cu(L^{CF3})$_2$(THF)]·2(THF).

Atom	Atom	Atom	Angle/˚		Atom	Atom	Atom	Angle/˚
O1	Cu1	O5	90.54(8)		C26	C25	C29	118.64(19)
O2	Cu1	O1	93.12(6)		C27	C26	C25	119.09(19)
O2	Cu1	O5	89.81(8)		C26	C27	C28	121.0(2)
O3	Cu1	O1	86.67(6)		C26	C27	C30	120.0(2)
O3	Cu1	O2	175.67(7)		C28	C27	C30	119.1(2)
O3	Cu1	O5	94.52(8)		C27	C28	C23	119.9(2)
O4	Cu1	O1	176.08(7)		F13	C29	C25	114.5(4)
O4	Cu1	O2	86.49(6)		F13	C29	F14	103.5(8)
O4	Cu1	O3	93.42(6)		F14	C29	C25	112.5(5)
O4	Cu1	O5	93.36(8)		F15	C29	C25	113.7(4)
C1	O1	Cu1	126.00(12)		F15	C29	F13	108.6(7)
C3	O2	Cu1	125.99(13)		F15	C29	F14	103.0(5)
C20	O3	Cu1	125.79(12)		F13A	C29	C25	113.4(7)
C22	O4	Cu1	125.73(12)		F13A	C29	F14A	108.2(10)
C51	O5	Cu1	122.7(4)		F14A	C29	C25	110.6(7)
C51	O5	C54	101.5(6)		F15A	C29	C25	111.9(6)
C54	O5	Cu1	121.1(3)		F15A	C29	F13A	109.3(11)
C39	O5	Cu1	127.3(4)		F15A	C29	F14A	103.0(9)
C39	O5	C42	104.4(6)		C27	C30	F16	105.9(3)
C42	O5	Cu1	127.3(4)		C27	C30	F18A	100.9(3)
O1	C1	C2	125.36(17)		F17	C30	C27	115.1(5)
O1	C1	C4	114.86(16)		F17	C30	F16	98.0(5)
C2	C1	C4	119.75(16)		F18	C30	C27	116.4(4)
C3	C2	C1	123.87(17)		F18	C30	F16	98.0(6)
O2	C3	C2	125.09(17)		F18	C30	F17	118.6(6)
O2	C3	C12	114.64(16)		F16A	C30	C27	119.7(5)
C2	C3	C12	120.26(16)		F16A	C30	F17A	122.4(7)
C5	C4	C1	117.76(17)		F16A	C30	F18A	91.7(6)
C5	C4	C9	118.86(18)		F17A	C30	C27	116.2(5)
C9	C4	C1	123.32(18)		F17A	C30	F18A	90.5(6)
C6	C5	C4	120.2(2)		C32	C31	C22	122.14(16)
C5	C6	C7	120.7(2)		C36	C31	C22	118.90(16)
C5	C6	C10	119.7(2)		C36	C31	C32	118.94(17)
C7	C6	C10	119.7(2)		C33	C32	C31	120.31(18)
C8	C7	C6	119.12(19)		C32	C33	C37	119.07(19)
C7	C8	C9	121.0(2)		C34	C33	C32	120.60(18)
C7	C8	C11	119.7(2)		C34	C33	C37	120.32(19)
C9	C8	C11	119.2(2)		C33	C34	C35	119.19(18)
C8	C9	C4	120.1(2)		C34	C35	C36	120.69(18)
F1	C10	C6	115.0(3)		C34	C35	C38	118.67(18)
F1	C10	F3	99.3(4)		C36	C35	C38	120.63(19)
F2	C10	C6	114.4(4)		C31	C36	C35	120.26(17)
F2	C10	F1	111.8(7)		C33	C37	F20	105.4(3)
F2	C10	F3	103.1(5)		C33	C37	F19A	102.3(3)
F3	C10	C6	111.5(3)		F19	C37	C33	117.7(3)
F1A	C10	C6	106.5(3)		F19	C37	F20	101.4(4)
F2A	C10	C6	113.4(5)		F19	C37	F21	116.0(5)
F2A	C10	F1A	95.5(5)		F21	C37	C33	114.5(3)
F3A	C10	C6	116.3(4)		F21	C37	F20	97.9(4)
F3A	C10	F1A	104.4(7)		F20A	C37	C33	119.3(5)
F3A	C10	F2A	117.3(6)		F20A	C37	F19A	92.2(7)
F4	C11	F5	105.7(2)		F20A	C37	F21A	117.7(8)
F4	C11	C8	113.0(2)		F21A	C37	C33	120.3(6)
F5	C11	C8	111.6(2)		F21A	C37	F19A	91.8(7)
F6	C11	F4	105.8(2)		F22	C38	C35	113.6(3)

F6	C11	F5	107.3(2)		F23	C38	C35	112.7(4)
F6	C11	C8	113.0(2)		F23	C38	F22	103.2(6)
C13	C12	C3	122.54(17)		F24	C38	C35	113.6(4)
C17	C12	C3	117.99(16)		F24	C38	F22	109.5(6)
C17	C12	C13	119.38(17)		F24	C38	F23	103.3(5)
C14	C13	C12	119.45(19)		F22A	C38	C35	111.8(4)
C13	C14	C18	118.6(2)		F22A	C38	F24A	98.5(7)
C15	C14	C13	121.44(19)		F23A	C38	C35	114.3(6)
C15	C14	C18	119.95(19)		F23A	C38	F22A	112.0(7)
C14	C15	C16	118.83(18)		F23A	C38	F24A	107.2(8)
C15	C16	C17	120.7(2)		F24A	C38	C35	111.9(5)
C15	C16	C19	119.52(19)		O5	C51	C52	99.7(7)
C17	C16	C19	119.8(2)		C53	C52	C51	105.5(7)
C12	C17	C16	120.20(19)		C52	C53	C54	104.8(7)
F7	C18	F8	105.8(2)		O5	C54	C53	101.1(7)
F7	C18	F9	107.0(2)		C1L	O6	C1I	109.8(14)
F7	C18	C14	112.93(19)		O6	C1I	C1J	102.0(11)
F8	C18	C14	111.3(2)		C1K	C1J	C1I	107.9(9)
F9	C18	F8	107.3(2)		C1J	C1K	C1L	103.2(10)
F9	C18	C14	112.2(2)		O6	C1L	C1K	102.0(12)
F10	C19	C16	115.3(3)		C1M	O7	C1P	109.9(9)
F10	C19	F11	111.3(5)		O7	C1M	C1N	106.5(8)
F10	C19	F12	102.8(4)		C1M	C1N	C1O	102.6(7)
F11	C19	C16	115.7(2)		C1P	C1O	C1N	98.8(8)
F11	C19	F12	98.4(4)		O7	C1P	C1O	104.9(8)
F12	C19	C16	111.3(3)		C1D	O8	C1A	104.6(12)
F10A	C19	C16	111.4(3)		C1E	O9	C1H	106.5(11)
F10A	C19	F11A	99.3(4)		O8	C1A	C1B	103.2(12)
F11A	C19	C16	108.0(3)		C1C	C1B	C1A	89.9(13)
F12A	C19	C16	114.0(3)		C1B	C1C	C1D	92.3(12)
F12A	C19	F10A	116.4(5)		O8	C1D	C1C	102.5(11)
F12A	C19	F11A	106.0(5)		O9	C1E	C1F	106.1(11)
O3	C20	C21	125.18(17)		C1E	C1F	C1G	98.3(9)
O3	C20	C23	114.87(16)		C1H	C1G	C1F	105.9(9)
C21	C20	C23	119.95(16)		O9	C1H	C1G	107.5(10)
C22	C21	C20	124.43(17)		O5	C39	C40	103.5(7)
O4	C22	C21	125.09(17)		C41	C40	C39	104.1(8)
O4	C22	C31	114.59(15)		C40	C41	C42	104.5(8)
C21	C22	C31	120.32(16)		O5	C42	C41	101.3(8)
C24	C23	C20	118.39(16)		C1T	O10	C1Q	116.3(19)
C24	C23	C28	119.19(17)		O10	C1Q	C1R	98.4(13)
C28	C23	C20	122.41(17)		C1S	C1R	C1Q	100.4(12)
C23	C24	C25	120.14(19)		C1R	C1S	C1T	96.8(14)
C24	C25	C29	120.7(2)		O10	C1T	C1S	91.6(14)
C26	C25	C24	120.7(2)					
